# Study on the Intramolecular H-Migration Kinetics of Strained Polycyclic Hydrocarbons with Distinct *Cis* and *Trans* Configurations

**DOI:** 10.3390/molecules31132302

**Published:** 2026-07-01

**Authors:** Xiaoxia Yao, Ying Xuan, Junjiang Guo, Mingxia Liu, Zerong Li, Zhian Li

**Affiliations:** 1 Aviation Maintenance Industry College, Chengdu Aeronautic Polytechnic University, Chengdu 610100, China; yaoxxkgd@163.com (X.Y.);; 2 School of Chemical Engineering, Guizhou Institute of Technology, Guiyang 550003, China; 3 School of Physics and New Energy, Chongqing University of Technology, Chongqing 401135, China; 4 College of Chemistry, Sichuan University, Chengdu 610064, China

**Keywords:** strained polycyclic hydrocarbo, intramolecular H-migration reaction, *cis* and *trans* configuration, reaction barrier, high-pressure-limit rate constant

## Abstract

High-energy-density fuels (HEDFs) have garnered considerable interest in aerospace fields, primarily due to their superior density and volumetric net heat of combustion (NHOC) compared with traditional petroleum-based fuels. Strained polycyclic hydrocarbons are regarded as one of the most crucial categories of HEDF. As an isomer (C_10_H_16_) of JP-10, the target compound is composed of two cyclopropyl rings and one cyclobutyl ring connected in a linear manner. Notably, intramolecular H-migration reactions of peroxyl radicals derived from strained polycyclic hydrocarbons (C_10_H_15_OO•) are of great significance for establishing the reaction mechanism of high-energy-density fuels over a broad temperature range. In this work, the intramolecular H-migration kinetics of C_10_H_15_OO• with distinct *cis* and *trans* configurations are investigated by quantum chemical calculations. Geometry optimization and frequency calculations are carried out for all species using the M06-2X/6-311++G(d,p) level of theory, while single-point energy calculations are performed at the CBS-QB3 level. Our calculated results demonstrate that different types of intramolecular H-migration reactions exhibit significant differences in barrier heights. Based on the ring structures where the reaction centers are located, these reactions can be classified into three categories: the lowest barriers correspond to H-migration reactions occurring between the central cyclopropyl ring and the terminal cyclobutyl ring; the highest barriers correspond to H-migration reactions confined entirely within the terminal cyclobutyl ring; and the barriers for H-migration reactions occurring between the terminal cyclopropyl ring and the central cyclopropyl ring lie between the above two. High-pressure-limit rate constants for 33 elementary reactions are determined in the temperature range of 500 to 2500 K based on the conventional transition-state theory (TST) and expressed in the modified Arrhenius form.

## 1. Introduction

High-energy-density fuels (HEDFs) are indispensable for volume-constrained aerospace vehicles, as their enhanced density and volumetric net heat of combustion (NHOC) allow them to deliver superior propulsion performance in comparison to traditional petroleum-derived fuels [1]. Among various HEDF candidates, strained polycyclic hydrocarbons have attracted extensive attention due to their unique polycyclic structures that endow them with high-energy density [1,2]. The application advantages of such polycyclic hydrocarbon fuels stem from the extra strain energy accumulated in their strained skeletons [2]. As the ring size decreases, the distortion of molecular bond angles intensifies, accompanied by a remarkable increase in strain energy. Meanwhile, the compact cyclic structure contributes to denser molecular packing, thereby achieving a higher fuel density and net heat of combustion. Previous studies have demonstrated [3,4] that despite the large intramolecular strain energy of such derivatives, most of them exhibit excellent thermal stability, and their synthesis is far less challenging than previously anticipated. At present, numerous molecular assemblies containing strained cycloalkanes have been successfully synthesized [5,6].

Polycyclic hydrocarbon molecules are mainly divided into two classes: one is saturated cage-type molecules formed by the fusion of cycloalkanes (i.e., two rings sharing two adjacent carbon atoms and the chemical bond between them), with typical representatives being Exo-tetrahydrodicyclopentadiene (JP-10), cubane, and its derivatives. JP-10 is a typical polycyclic hydrocarbon and the most widely used HEDFs in aerospace propulsion. Composed of three five-membered rings, this molecule exhibits significantly higher density and net heat of combustion than traditional aviation kerosene. A large number of studies regarding the synthetic pathways [7,8,9,10], reaction kinetics [11,12], thermal cracking (pyrolysis), and combustion kinetic models [13,14,15,16] of JP-10 have been published publicly. The other class is the linear aggregates of cycloalkanes connected by carbon–carbon single bonds. However, there have been relatively few reports on the kinetic studies of linearly aggregated polycyclic hydrocarbon fuels. Yue et al. [17] explored the decomposition mechanism of dicyclohexyl (a compound consisting of two cyclohexyl radicals linked via a single C-C bond) by means of quantum chemical calculations at the M06-2X/6-31+G(d) level of theory. Their findings indicated that the C-C bond bridging the two cyclohexyl rings in the dicyclohexyl molecule is liable to cleavage. Meanwhile, Lele et al. [18] examined the initial pyrolysis processes of several bicyclic compounds that contain cycloalkane moieties connected by single C-C bonds. According to molecular dynamics simulations based on the ReaxFF reactive force field, the initial pyrolysis of these bicyclic compounds proceeds via two primary reaction pathways: bridging bond cleavage and ring-opening reactions. Chen et al. [19] conducted a systematic investigation on the thermodynamics and initial pyrolysis kinetics of five strained polycyclic hydrocarbons-JP-10 isomers composed of two cyclopropyl rings and one cyclobutyl ring by applying high-level quantum chemical calculations at the M06-2X/6-311++G(d,p) and CASPT2 levels. It was found that the initial pyrolysis is dominated by ring bond breaking rather than bridge bond breaking, following diradical and concerted isomerization pathways. These compounds show a higher net heat of combustion and specific impulse than JP-10 and decompose more easily.

Collectively, these studies demonstrate that the high-temperature reaction mechanism of polycyclic hydrocarbons with strained ring structures is vital for predicting reaction products. Nevertheless, systematic research on the reaction mechanisms of linearly aggregated polycyclic hydrocarbon fuels across a broad temperature range remains insufficient. Notably, intramolecular H-migration reactions of polycycloalkylperoxy radicals, a key type of reaction for strained polycyclic hydrocarbons, play a crucial role in regulating the negative temperature coefficient (NTC) behavior during the low-temperature oxidation of strained polycyclic hydrocarbons—this behavior is a core characteristic of fuel ignition chemistry and directly affects the ignition performance and oxidation mechanism of strained polycyclic hydrocarbons in aerospace engine operating conditions. As revealed by Min et al. [16], intramolecular H-migration reactions of JP-10-derived peroxyl radicals (C_10_H_15_OO•) are essential for constructing accurate low-temperature oxidation mechanisms of strained polycyclic hydrocarbons, as their unique steric cyclic structure and ring strain-induced deviations from conventional H-migration rules (e.g., 1,4 H- and 1,5 H-migration energy barrier order) make them indispensable for describing NTC behavior and ignition characteristics. Therefore, in-depth study of intramolecular H-migration reactions of strained polycyclic hydrocarbons is not only necessary to fill the gap in low-temperature reaction kinetics but also critical for optimizing the oxidation mechanisms of strained polycyclic hydrocarbons, ensuring their reliable application in high-performance aerospace engines. Furthermore, strained polycyclic hydrocarbons are key components of soot precursors at high combustion temperatures, and their intramolecular H-migration governs the formation, growth, and oxidation of polycyclic aromatic hydrocarbons (PAHs) and soot precursors. The low-temperature oxidation and high-temperature pyrolysis of such strained ring systems are strongly coupled, and the kinetic parameters from H-migration reactions provide important data for understanding the high-temperature response of soot precursors.

The JP-10 isomer molecule studied in this work consists of two cyclopropyl rings and one cyclobutyl ring, with one cyclopropyl ring and the cyclobutyl ring located at the two ends of the molecule, respectively, and the other cyclopropane ring in the middle. The strained polycyclic hydrocarbons are selected for two main reasons. First, cyclopropane and cyclobutane have prominent ring strain energy, which endows these molecules with high volumetric energy density and specific impulse. Such compact cyclic architectures are widely recognized as ideal candidates of strained polycyclic HEDF model compounds for aerospace propulsion. The investigated JP-10 isomers in this work are classic model compounds for exploring the pyrolysis and reaction kinetics of strained small-ring polycyclic HEDF model compounds, which have been reported in previous computational and experimental studies [19]. Second, the structural feasibility of these two stereoisomers has been fully verified. Geometry optimization and vibrational frequency calculations confirm that all studied structures are stable minima on the potential energy surface without imaginary frequencies. In addition, abundant synthetic studies have proved that polycyclic hydrocarbons containing fused cyclopropane and cyclobutane moieties are synthetically accessible and thermally stable under typical combustion conditions [20]. High-level quantum chemical methods were employed to calculate the energy barriers and rate constants for intramolecular H-migration reactions of the studied fuels in the gas phase.

## 2. Results and Discussion

### 2.1. Cis and Trans Configurations

**Figure 1:**
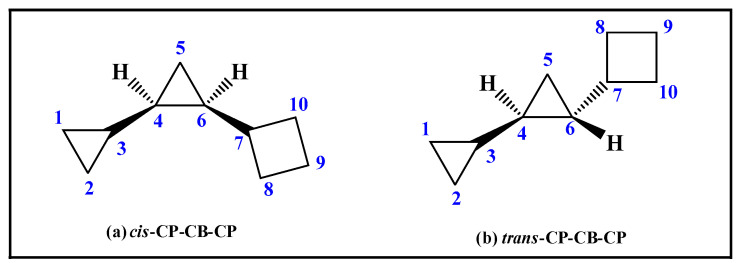
Molecular configurations of 1-cyclopropyl-3-cyclobutylcyclopropane, where the ten distinct carbon atoms of *cis*-CP-CB-CP and *trans*-CP-CB-CP are sequentially numbered 1–10.

In this work, 1-cyclopropyl-3-cyclobutylcyclopropane is selected as the target molecule, whose cyclic framework is arranged from left to right as the terminal cyclopropyl group, central cyclopropyl parent group, and terminal cyclobutyl group. Owing to the distinct spatial arrangements of substituents on both sides of the central cyclopropyl ring, this molecule can exist as two stereoisomers, namely the cis and trans configurations. We investigated two important high-energy-density fuels: *cis*-1-cyclopropyl-3-cyclobutylcyclopropane and *trans*-1-cyclopropyl-3-cyclobutylcyclopropane. For brevity, *cis*-CP-CB-CP and *trans*-CP-CB-CP are used hereafter to denote *cis*-1-cyclopropyl-3-cyclobutylcyclopropane and *trans*-1-cyclopropyl-3-cyclobutylcyclopropane, respectively. The molecular configurations of *cis*-CP-CB-CP and *trans*-CP-CB-CP are shown in Figure 1.

**Figure 2:**
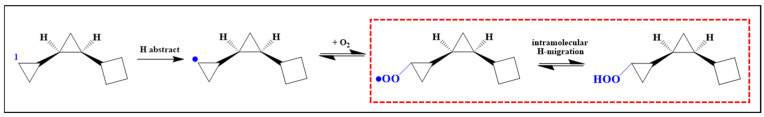
Simplified low-temperature oxidation reaction sequence of *cis*-CP-CB-CP. Label 1 corresponds to carbon site 1 defined in Figure 1; the red dashed frame highlights the core reaction process investigated in this work.

Both *cis*-CP-CB-CP and *trans*-CP-CB-CP consist of 10 unique carbon atoms, which are sequentially labeled as site *N* (*N* = 1, 2, 3, …, 10) in Figure 1. At low temperatures and high temperatures, hydrogen atoms attached to these 10 carbon sites can be abstracted by diverse active radicals (including H, O(^3^P), CH_3_, OH, HO_2_, C_2_H_5_, and CH_3_O) [21,22,23], generating the corresponding alkyl radicals as illustrated in Figure 2. These alkyl radicals subsequently undergo addition with O_2_ to form alkylperoxy radicals (ROO•) [24], and intramolecular H-migration of ROO• radicals can further yield hydroperoxyalkyl radicals (•QOOH). Accordingly, the present study focuses on investigating the kinetic properties of intramolecular H-migration reactions of peroxy radicals derived from *cis*-CP-CB-CP and *trans*-CP-CB-CP, which are highlighted in the dashed box in Figure 2.

To clarify the stability difference between peroxyl radical structures with OO• located on three-membered versus four-membered rings, we selected representative reactants and compared their electronic energies, as summarized in Table S1 of the Supporting Information. According to the HF electronic energy data summarized in Table S1, it can be seen that the structures with the peroxyl radical (OO•) attached to three-membered cyclopropane rings (*cis*-R1, *cis*-R2, *cis*-R3, *cis*-R6) have electronic energies ranging from −539.347644 to −539.34352 Hartree. These values are less negative, corresponding to higher electronic energy and weaker thermodynamic stability. Structures with OO• bound to four-membered cyclobutane rings (*cis*-R10, *cis*-R12, *cis*-R14, *cis*-R15) show electronic energies from −539.353544 to −539.350298 Hartree. These more negative energies indicate lower electronic energy and superior thermodynamic stability. In short, the peroxyl radical species with OO• attached to four-membered cyclobutane rings are more stable than those with OO• anchored on three-membered cyclopropane rings.

Previous studies [25,26,27] have demonstrated that among intramolecular H-migration reactions of hydrocarbons under low-temperature conditions, the 1,5 H-migration generally possesses the lowest energy barrier. This indicates that 1,5 H-migration is the most favorable pathway for ROO• radicals to produce •QOOH radicals via intramolecular H-migration. Accordingly, only the 1,5 H-migration reactions of peroxy radicals of *cis*-CP-CB-CP and *trans*-CP-CB-CP are considered in the present study.

**Figure 3:**
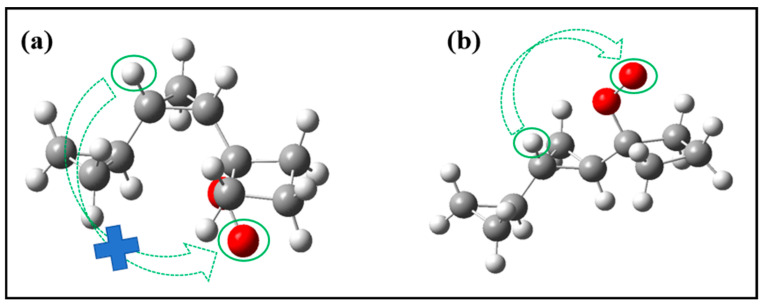
Schematic diagram of 1,5 H-migration occurring with the peroxy group attached to the C7 carbon atom. (**a**) The blue cross symbol signifies that the 1,5 H-migration pathway marked by the green dashed arrow is chemically inaccessible; green circles enclose the migrating hydrogen atom and terminal oxygen atom of the peroxy group. (**b**) The green dashed arrow illustrates the feasible intramolecular 1,5 H-migration process, where the hydrogen atom circled in green transfers to the terminal oxygen atom of the -OO group circled in green.

Notably, 1,5 H-migration reaction cannot occur at all active sites. For *cis*-CP-CB-CP, when the peroxy group (-OO•) is attached to the C7 carbon atom, the peroxy group and the migrating hydrogen atom are located on opposite sides of the central cyclopropyl parent ring. Restricted by steric hindrance, 1,5 H-migration is prohibited at this site, as illustrated in Figure 3a. In contrast, for *trans*-CP-CB-CP with the peroxy group also bound to the C7 carbon atom, the peroxy group and the migrating hydrogen atom lie on the same side of the central cyclopropyl parent ring. In this case, no obvious steric hindrance is observed, and thus the 1,5 H-migration reaction can proceed favorably.

**Figure 4:**
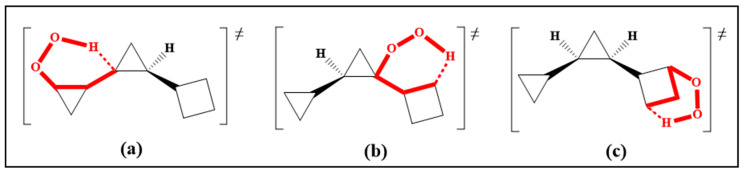
Transition states of hydrogen migration reactions. The symbol ≠ denotes the transition state; red highlighted regions mark the reactive centers in each transition state structure. (**a**) The reactive center spans the terminal cyclopropyl ring and the central cyclopropyl ring; (**b**) The reactive center spans the central cyclopropyl ring and the terminal cyclobutyl ring; (**c**) The reactive center is fully confined within the terminal cyclobutyl ring.

Previous studies on H-migration reactions of peroxy radicals of alkanes and cycloalkanes have confirmed that the transition state of 1,5 H-migration adopts a six-membered ring structure [24,27]. Given that the high density fuel molecule investigated in this work is linearly aggregated by three cyclic structural units, the reaction center (corresponding to the position of the transition state) can be located in three distinct fashions: spanning the terminal cyclopropyl and the central cyclopropyl ring, as illustrated in Figure 4a; spanning the central cyclopropyl ring and the terminal cyclobutyl ring, as depicted in Figure 4b; being entirely confined within the terminal cyclobutyl ring, as shown in Figure 4c.

### 2.2. Reaction Energy Barriers

In this study, all possible 1,5 H-migration reactions corresponding to the 10 carbon sites of both cis and trans configurations as shown in Figure 1 are investigated. Detailed information of these reactions is summarized in Table 1. Among them, 16 reactions belong to the cis configuration, and 17 reactions correspond to the trans configuration. It can be seen from Table 1 that when the peroxy group is attached to the C1 carbon atom, one type of 1,5 H-migration reaction is available for both the cis and trans configurations. For the C2 site, one reaction type is observed for each configuration. Similarly, one 1,5 H-migration type exists at the C3 carbon for both isomers. When the peroxy group binds to C4, two reaction types are found for both cis and trans configurations. Two reaction types are also available at the C5 and C6 carbon atoms for both configurations. At the C7 site, two 1,5 H-migration reaction types are identified for the cis configuration, among which one reaction is inhibited by steric hindrance (as discussed in Section 2.1). By contrast, three reaction types are accessible for the trans configuration at this position. Two reaction types are observed at the C8 carbon for both configurations. One reaction type occurs at the C9 carbon for both isomers, while two reaction types are available at the C10 carbon atom.

The energy barriers of the 33 H-migration reactions in Table 1 are calculated, and the corresponding results are summarized in Table 2. It can be observed from Table 2 that for H-migration reactions at the same site, the energy barriers of the *cis* and *trans* configurations are generally close, with no significant difference. However, there are individual reactions where the energy barriers difference between the two configurations are relatively obvious. For instance, the energy barrier of *cis*-R6 is 25.90 kcal mol^−1^, and that of *trans*-R6 is 24.34 kcal mol^−1^; *cis*-R7 is 20.09 kcal mol^−1^, while that of *trans*-R7 is 18.25 kcal mol^−1^. This indicates that, under certain special circumstances, steric hindrance directly restricts the feasibility of H-migration reactions. The fuel molecule investigated in this study is composed of three rigid cyclic structures connected linearly, without any additional side-chain substituents. In the cis configuration, the cyclic skeleton exhibits local spatial folding, where adjacent cyclic rings are close to each other, resulting in stronger spatial squeezing interactions between the rings and higher local spatial crowding. In contrast, the cyclic skeleton of the trans configuration is more stretched, with a low degree of spatial stacking between each rigid cyclic ring and weaker intramolecular spatial repulsion.

It also can be observed from Table 2 that for reactions within the same molecular configuration, the reaction centers of hydrogen migration exert a significant impact on the reaction energy barrier. According to the data, the following conclusions can be drawn: (1) When the reaction centers are located on the two cyclopropyl rings among the three cyclic structures of the fuel molecule, the corresponding reactions possess similar energy barriers. (2) When the reaction centers span the central cyclopropyl ring and the terminal cyclobutyl ring, the energy barriers of these reactions are generally close. (3) When the reaction centers are merely confined within the terminal cyclobutyl ring alone, the reaction energy barriers remain analogous.

The overall trend of barrier heights for the above three categories is as follows: reaction centers spanning the central cyclopropyl ring and the terminal cyclobutyl ring (e.g., *cis*-R8, 20.18 kcal mol^−1^) < reaction centers located on the two cyclopropyl rings of the fuel molecule (e.g., *cis*-R1, 22.16 kcal mol^−1^) < reactions confined to the terminal cyclobutyl ring (e.g., *cis*-R16, 30.69 kcal mol^−1^). This trend can be explained well with structural and electronic effects. As for *cis*-R8 on the four-membered ring, the peroxyl radical and migrating hydrogen are spatially well separated. There is negligible 1,3-strain or steric repulsion during the reaction. The hydrogen migration only induces slight geometric deformation, and the resulting carbon-centered radical is stable in the local environment, leading to the lowest barrier of 20.18 kcal mol^−1^. *Cis*-R1 is located on the three-membered ring. Despite the absence of severe steric hindrance, cyclopropane possesses larger intrinsic angle strain than cyclobutane. This inherent ring tension raises its barrier to 22.16 kcal mol^−1^, which is moderately higher than that of *cis*-R8. In contrast, *cis*-R16 exhibits an extremely high barrier of 30.69 kcal mol^−1^. On one hand, the peroxyl radical (-OO•) and the migrating hydrogen atom occupy sterically crowded 1,3-positions. The rigid four-membered ring cannot relieve spatial repulsion through rotation, thus generating intense 1,3-strain and causing severe distortion of the transition state. On the other hand, long-range hydrogen migration requires substantial conformational rearrangement of the molecule, which further increases the energy. The resulting carbon-centered radical resides in a highly strained region and is greatly destabilized by adjacent electron-withdrawing oxygen-containing groups. The combination of these unfavorable factors leads to a much higher reaction barrier than that of *cis*-R8 on the same four-membered ring and *cis*-R1 on the three-membered ring. The relative order of these energy barriers is fully consistent with fundamental chemical principles and chemical expectations.

The differences in energy barriers among various hydrogen migration pathways reflect the oxidation susceptibility of distinct molecular sites within the fuel molecule. From the perspective of developing new strained polycyclic HEDF model compounds, this structure-activity relationship provides clear guidance for molecular skeleton optimization. By introducing substituent groups to sterically shield reactive sites with low energy barriers, the thermal oxidation stability of target strained polycyclic HEDF model compounds can be significantly enhanced, thereby effectively extending the long-term storage service life of aviation fuels.

### 2.3. Rate Constants

High-pressure-limit rate coefficients of the 33 H-migration reactions of peroxy radicals of *cis*-CP-CB-CP and *trans*-CP-CB-CP which are shown in Table 2 are calculated. These rate coefficients are fitted to the three-parameter (*A*, *n*, *Ea*) form using the modified Arrhenius equation (*k = AT^n^exp*(*−Ea*/*RT*)). High-pressure-limit rate coefficients for reactions over the temperature range of 500–2500 K are listed in Table 3. The obtained Arrhenius kinetic parameters of key elementary reactions can be supplemented into the detailed combustion kinetic mechanism of strained polycyclic HEDF model compounds, which helps to accurately predict the ignition performance, combustion efficiency and heat release characteristics of newly designed fuels in aero-engine working environments, and provides theoretical data support for the screening and performance evaluation of candidate strained polycyclic HEDF model compounds.

**Figure 5:**
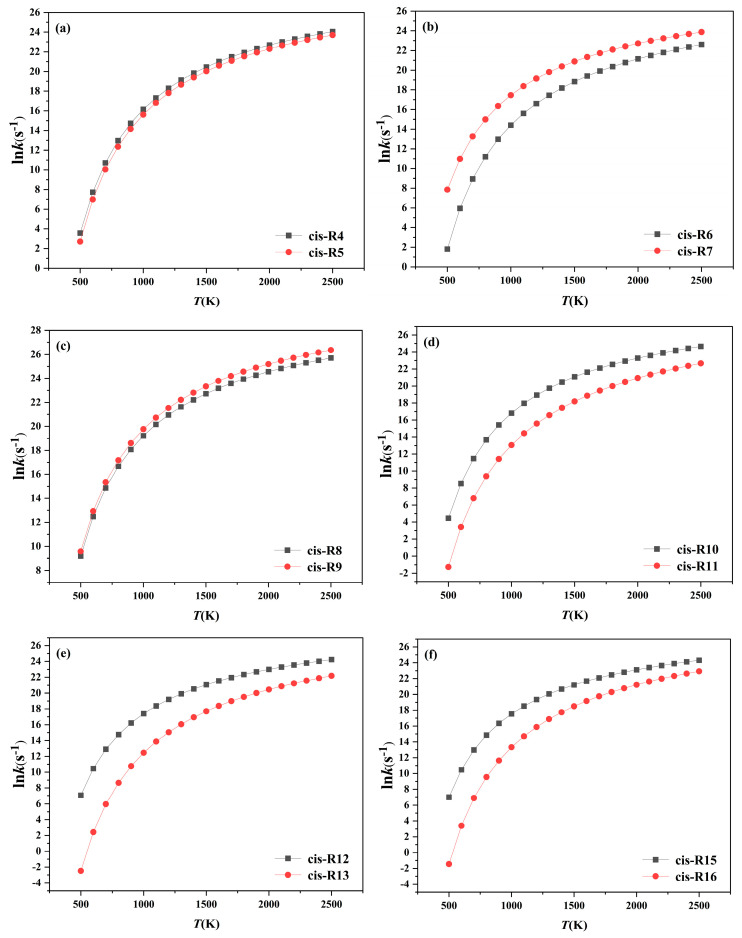
Comparison of high-pressure-limit rate coefficients for *cis*-conformation reactions with different reactive centers, where the -OO group of the reactant is attached to the identical carbon atom. (**a**) The -OO group is attached to carbon site 4 defined in Figure 1; (**b**) The -OO group is attached to carbon site 5 defined in Figure 1; (**c**) The -OO group is attached to carbon site 6 defined in Figure 1; (**d**) The -OO group is attached to carbon site 7 defined in Figure 1; (**e**) The -OO group is attached to carbon site 8 defined in Figure 1; (**f**) The -OO group is attached to carbon site 10 defined in Figure 1.

Figure 5 illustrates the comparisons of high-pressure-limit rate coefficients for *cis* configuration reactions with different reaction centers when the peroxy group (-OO•) is bonded to the same carbon atom. These figures illustrate the effects of reaction center positions on the rate coefficients for intramolecular 1,5 H-migration reactions of the *cis*-CP-CB-CP peroxy radicals under identical peroxyl substitution sites. As shown in Figure 5, even when the peroxy groups are attached to the same carbon sites of the *cis*-CP-CB-CP skeleton, the high-pressure-limit rate coefficients of intramolecular 1,5 H-migration reactions exhibit significant discrepancies, which are directly determined by the spatial distribution of the reaction centers. Specifically, reactions with reaction centers spanning the central cyclopropyl rings and the terminal cyclobutyl rings show the highest rate coefficients over the entire temperature range of 500–2500 K. In contrast, reactions with reaction centers spanning the two cyclopropyl rings have relatively intermediate rate coefficients, while those with reaction centers completely confined within the terminal cyclobutyl ring display the lowest rate coefficients. These trends are strictly consistent with the energy barrier characteristics and the typical reaction examples. Reactions with lower energy barriers tend to have larger rate coefficients, conforming to the basic principle of chemical kinetics that the energy barrier is a dominant factor affecting reaction rate.

**Figure 6:**
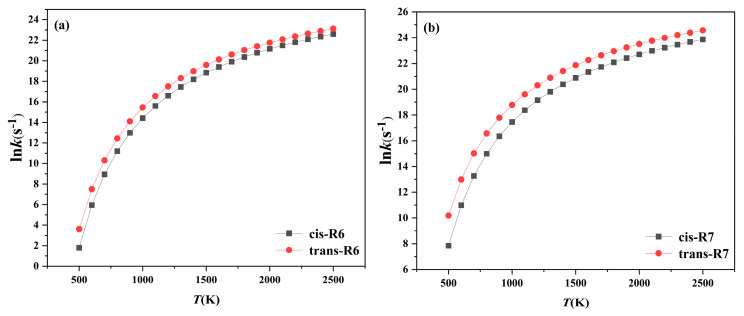
Comparison of high-pressure-limit rate coefficients for reactions with identical reaction centers in *cis* and *trans* configurations. (**a**) Reactions of *cis*-R6 versus *trans*-R6; (**b**) Reactions of *cis*-R7 versus *trans*-R7.

Figure 6 presents the comparisons of high-pressure-limit rate coefficients for reactions with identical reaction centers in *cis* and *trans* configurations, focusing on two pairs of typical reactions (*cis*-R6 vs. *trans*-R6; *cis*-R7 vs. *trans*-R7). This figure aims to clarify the influence of *cis/trans* configuration differences on the intramolecular 1,5 H-migration kinetics of peroxy radicals when the reaction centers are the same. It can be clearly observed from Figure 6 that for reactions with identical reaction centers, the high-pressure-limit rate coefficients of *trans*-CP-CB-CP peroxy radicals are generally slightly higher than those of *cis*-CP-CB-CP peroxy radicals over the entire temperature range of 500–2500 K, which is consistent with the energy barrier differences between the two configurations. As shown by the reaction energy barriers in Table 2, *cis*-R6 has an energy barrier of 25.90 kcal mol^−1^, while *trans*-R6 has an energy barrier of 24.34 kcal mol^−1^; *cis*-R7 has an energy barrier of 20.06 kcal mol^−1^, and *trans*-R7 has an energy barrier of 18.25 kcal mol^−1^. The lower energy barrier of *trans* configuration reactions leads to higher rate coefficients, which is consistent with the kinetic principle that lower energy barriers correspond to faster reaction rates.

The rate coefficient differences between cis and trans configurations in Figure 6 essentially originate from the steric hindrance differences between the two configurations. As discussed in Section 2.1, the cis configuration skeleton has local spatial folding, resulting in stronger spatial squeezing between adjacent rings and higher steric hindrance, which increases the energy barrier and reduces the rate coefficient. In contrast, the trans isomer exhibits a more extended skeleton with weaker intramolecular spatial repulsion and lower steric hindrance, resulting in lower energy barriers and higher rate coefficients. Notably, the rate coefficient gap between the two configurations is not extremely large, indicating that the configuration effect is secondary to the reaction center effect in regulating the intramolecular H-migration kinetics of peroxy radicals, which is consistent with the conclusion that reaction centers have a more significant impact on energy barriers.

**Figure 7:**
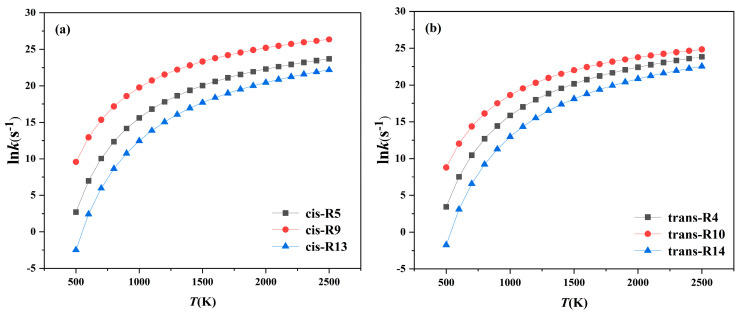
High-pressure-limit rate coefficients of the reactions as a function of temperature. (**a**) Three *cis* configuration reactions (*cis*-R5, *cis*-R9, and *cis*-R13); (**b**) Three *trans* configuration reactions (*trans*-R4, *trans*-R10, and *trans*-R14).

Figure 7 shows the high-pressure-limit rate coefficients of selected reactions as a function of temperature. As illustrated in Figure 7a, the three *cis* configuration reactions (*cis*-R5, *cis*-R9, and *cis*-R13) belong to different reaction centers and exhibit distinct temperature-dependent trends of rate coefficients. Among them, *cis*-R9 (reaction center spanning the central cyclopropyl and terminal cyclobutyl rings) has the highest rate coefficients over the entire temperature range, which is consistent with the lowest energy barrier (20.36 kcal mol^−1^) among the three reactions. *cis*-R5 (reaction center spanning the two cyclopropyl rings) has intermediate rate coefficients, corresponding to its moderate energy barrier (24.86 kcal mol^−1^). *cis*-R13 (reaction center confined within the terminal cyclobutyl ring) has the lowest rate coefficients, which is consistent with its higher energy barrier (31.13 kcal mol^−1^). Figure 7b presents three trans configuration reactions (*trans*-R4, *trans*-R10, and *trans*-R14), which also belong to different reaction centers and show similar temperature-dependent characteristics to the cis configuration reactions. The reaction of *trans*-R10 (reaction center spanning the central cyclopropyl and terminal cyclobutyl rings) has the highest rate coefficients, corresponding to its lower energy barrier (18.85 kcal mol^−1^). The reaction of *trans*-R4 (reaction center spanning the two cyclopropyl rings) has intermediate rate coefficients, with an energy barrier of 25.07 kcal mol^−1^. The reaction of *trans*-R14 (reaction center confined within the terminal cyclobutyl ring) has the lowest rate coefficients, consistent with its higher energy barrier (30.77 kcal mol^−1^).

Figure 7 reveals that the rate constants of all studied reactions increase as temperature goes up. The underlying mechanism behind this temperature effect is explained based on chemical kinetics. Higher temperature accelerates molecular thermal motion and increases effective collisions among reactive species. A greater number of reactant molecules can surmount the energy barrier to form transition states, thereby speeding up the reactions. For cyclic hydrocarbon reactants, high temperature reduces the rigidity of cyclopropyl and cyclobutyl rings, alleviates 1,3-strain and steric hindrance in the hydrogen migration process, and decreases the energy cost of molecular deformation. Collectively, these effects result in monotonically increasing reaction rates with temperature.

We have investigated the temperature dependence of reaction rates via density functional theory calculations combined with the Arrhenius equation. This strategy has been widely validated in previous studies. Alecu et al. [28] adopted the same computational procedure to calculate temperature-dependent rate constants for radical reactions, and their theoretical results showed good agreement with experimental kinetic data measured by laser photolysis. Chen et al. [29] also applied a similar theoretical framework to explore reaction kinetics in combustion systems, and the calculated kinetic parameters were well supported by experimental observations. These literature cases demonstrate the reliability of the method used in this work.

It should be noted that the present approach has certain limitations. First, the reaction rate is described based on the classic Arrhenius law, which assumes a constant activation energy within the investigated temperature range. In practical reactions, activation energy varies slightly with temperature, which may lead to deviations under extremely high- or low-temperature conditions. Second, this work mainly focuses on gas-phase homogeneous reactions under ideal conditions, while the effects of pressure, complex intermediates and intermolecular interactions are not fully considered. Therefore, the predicted rate constants may differ from the actual reaction behaviors under complex combustion environments. Third, this method performs well for typical radical hydrogen migration reactions, whereas its accuracy may decrease for reactions accompanied by strong steric hindrance or phase changes.

## 3. Methods

All calculations about electronic structures of reactants, transition states, and products are carried out using the Gaussian 16 Revision C.01 quantum chemistry package (Gaussian, Inc., Wallingford, CT, USA) [30]. The density functional theory method M06-2X with 6-311++g(d,p) basis set (scaled by a factor 0.97) [31] is adapted to calculate the geometries and vibrational frequencies of reactants, products and transition states involved in the studied systems. This DFT method is found to combine the advantages of low computational cost and high accuracy [16,19,32,33]. Zhao and Truhlar [33] systematically benchmarked the M06 suite of density functionals and demonstrated that M06-2X provides excellent performance for main-group thermochemistry, thermochemical kinetics, and barrier height predictions. Alecu et al. [31] further confirmed that M06-2X/6-311++G(d,p) with a scaling factor of 0.97 accurately predicts vibrational frequencies and zero-point energies for hydrocarbon systems. All stable molecules are confirmed to have no imaginary frequencies, while transition states are confirmed to have exactly one imaginary frequency. Intrinsic reaction coordinate (IRC) calculations [34] are performed at the same theoretical level, M06-2X/6-311++G(d,p), to verify that the transition states correctly connect the two corresponding stationary points. Cartesian coordinates of all species are provided in the Supplemental Material. High-level ab initio methods (such as CCSD(T)) are difficult to apply to the calculation of macromolecular systems [26]. Therefore, in this study, the CBS-QB3 composite method is employed for single-point energy calculations, as this method has relatively low computational cost. The CBS-QB3 method has been successfully applied to calculate the thermochemistry of JP-10 (C_10_H_16_), a strained polycyclic hydrocarbon, and the calculated formation enthalpies showed excellent agreement with experimental measurements [35]. Li et al. [36] investigated the kinetics of intramolecular H-migration reactions of methyl-ester peroxy radicals using the identical CBS-QB3//M06-2X/6-311++G(d,p) method, and their calculated energy barriers and rate constants showed good agreement with high-level literature values. Min et al. [16] specifically investigated intramolecular H-migration reactions of JP-10-derived peroxyl radicals (C_10_H_15_OO•) using the CBS-QB3 level of theory, demonstrating that this method effectively captures the ring strain effects and steric features of such strained polycyclic systems.

Due to the lack of well-established experimental values for some of prominent species in this study, in order to validate the calculated energy barriers by using the CBS-QB3 method for the system in this study, the couple cluster method CCSD(T)/cc-pVTZ//M06-2X/6-311++G(d,p) is used to compare the calculated energy barriers.

Table 4 summarizes the comparison of reaction barrier heights derived from CBS-QB3 and the high-level CCSD(T)/cc-pVTZ benchmark. The energy discrepancies between CBS-QB3 and the coupled-cluster reference reach only −1.84 and −1.80 kcal mol^−1^. Deviations of approximately 1.80 kcal mol^−1^ are reasonable and excellent for CBS-QB3 when applied to highly strained tricyclic systems. Accordingly, the CBS-QB3 composite method is sufficiently reliable for predicting electronic energies and activation barriers of the target species investigated in the present study.

The high-pressure-limit rate coefficients are calculated using transition-state theory (TST) [37], which has been widely used to calculate rate coefficients for complex macromolecular systems containing multiple rings [38,39]. The ChemRate program version 1.5.8 (National Institute of Standards and Technology: Gaithersburg, MD, USA, 2009) developed by Mokrushin and Tsang [40] is utilized in this work to compute the high-pressure-limit rate constants for all investigated reactions over a wide temperature range of 500–2500 K. The Eckart method [41] is used to account for the quantum mechanical tunneling effect.

Two representative intramolecular H-migration pathways (*cis*-R1 and *trans*-R1) are selected to systematically calculate temperature-dependent tunneling correction factors spanning 500–2500 K, and all full numerical datasets are tabulated in Supplementary Tables S2 and S3 to enable quantitative evaluation of quantum tunneling contributions. As summarized in these two tables, the tunneling correction factor κ of *cis*-R1 and *trans*-R1 follows consistent temperature-dependent trends: κ peaks at 3.00 for *cis*-R1 and 2.85 for *trans*-R1 at 500 K, demonstrating that quantum tunneling drastically accelerates hydrogen migration under low-temperature oxidation conditions (500–800 K). In the medium-temperature regime of 800–1500 K, κ gradually declines to 1.13–1.15 and only produces mild quantitative adjustments to absolute rate constants. At combustion temperatures ranging from 1500 K to 2500 K, κ converges to approximately 1.05 for both reactions, where thermal excitation dominates reaction dynamics and quantum tunneling effects become nearly negligible.

The Eckart model only describes tunneling along a single reaction coordinate and neglects multidimensional tunneling effects such as reaction-path curvature and corner-cutting tunneling, which are known to modify hydrogen-transfer kinetics. Nevertheless, the rigid fused tricyclic backbone of our JP-10-derived peroxyl radicals imposes severe geometric restrictions on migrating hydrogen atoms, suppressing large-amplitude off-pathway corner-cutting motions and partially mitigating deviations originating from omitted multidimensional effects.

Variational transition-state theory (VTST) [42] is not implemented in this work, as VTST optimizes the transition-state dividing surface along the reaction coordinate to minimize rate coefficients: a correction that is only meaningful for loose, weakly constrained transition states. For our compact six-membered cyclic H-migration transition states locked by cyclopropyl and cyclobutyl ring strain, variational displacements of the dividing surface are negligible over the 500–2500 K range. Meanwhile, comprehensive multidimensional kinetic treatments integrating VTST and small-curvature tunneling (SCT) via PolyRate/GaussRate are computationally infeasible for these large tricyclic species. Such calculations require dense Hessian grids sampled across the full intrinsic reaction coordinate, which would incur prohibitive computational costs beyond our available computing resources.

The high-pressure-limit rate constants at each temperature point within the range of 500–2500 K are fitted to the three-parameter (*A*, *n*, *Ea*) form using the modified Arrhenius equation (*k = ATnexp*(*−Ea/RT*)). The above kinetic expressions can be directly applied to the combustion reaction kinetic mechanisms of fuels, and these mechanisms can then be directly used as input files for practical combustion simulation software. Chemkin-Pro 15092 (Reaction Design: San Diego, CA, USA, 2009) [43] is employed in this study for combustion simulation calculations.

## 4. Conclusions

The intramolecular H-migration kinetics of C_10_H_15_OO• with distinct cis and trans configurations are investigated with the high-level quantum chemical calculation over a temperature range of 500–2500 K. Species involved in these reactions are optimized by M06-2X/6-311++g(d,p) and the single-point energies are computed with CBS-QB3.

All investigated reactions are classified into three categories according to the location of reaction centers. The H-migration reactions spanning the central cyclopropyl ring and terminal cyclobutyl ring exhibit the lowest energy barriers, with typical values ranging from 18.25 to 24.30 kcal mol^−1^. The H-migration reactions occurring between two cyclopropyl rings show moderate barriers of 22.16–25.90 kcal mol^−1^. By contrast, the H-migration reactions entirely confined within the terminal cyclobutyl ring possess the highest barriers up to 31.13 kcal mol^−1^. For identical reactive sites, the energy barriers between *cis* and *trans* configurations are generally close, while obvious differences are observed at individual positions: for example, *cis*-R6 (25.90 kcal mol^−1^) is 1.56 kcal mol^−1^ higher than *trans*-R6 (24.34 kcal mol^−1^), and *cis*-R7 (20.06 kcal mol^−1^) is 1.81 kcal mol^−1^ higher than *trans*-R7 (18.25 kcal mol^−1^). Such discrepancies originate from stronger steric hindrance and spatial compression in the folded skeleton of the cis isomer.

The high-pressure-limit rate coefficients of all 33 reactions are obtained and fitted to the modified Arrhenius equation within 500–2500 K. Consistent with the barrier trend, reactions with lower energy barriers yield larger rate constants across the whole temperature range. Reactions across the central cyclopropyl and terminal cyclobutyl rings present the highest rate coefficients, while those located inside the terminal cyclobutyl ring show the lowest values. Under the same reaction center, *trans* configuration radicals generally have slightly higher rate coefficients than *cis* configuration radicals, and the configuration effect is weaker than the effect of reaction center position on kinetic behaviors. Additionally, the rate constants of all reactions increase monotonically with rising temperature from 500 K to 2500 K, which can be explained well by kinetic collision theory and structural relaxation of strained rings at elevated temperatures.

The present study quantitatively reveals the relationships among molecular configuration, ring strain, steric hindrance, reaction barrier and kinetic parameters for intramolecular H-migration of strained polycyclic hydrocarbon peroxyl radicals. The obtained barrier data, Arrhenius parameters and rate coefficients provide reliable fundamental data for constructing the wide-range temperature oxidation and combustion kinetic mechanisms of such high-energy-density fuels. The established structure–activity relationships also offer clear theoretical guidance for the molecular design and performance optimization of novel strained polycyclic HEDF model compounds.

## Figures and Tables

**Table 1:** 1,5 H-migration reactions of peroxy radicals of *cis*-CP-CB-CP and *trans*-CP-CB-CP.

Attachment Position of −OO•	Reaction of Peroxy Radicals of *Cis*-CP-CB-CP ^b^	Reaction of Peroxy Radicals of *Trans*-CP-CB-CP
Site. 1 ^a^	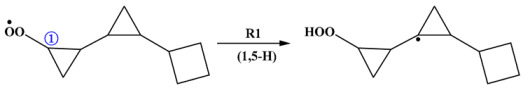	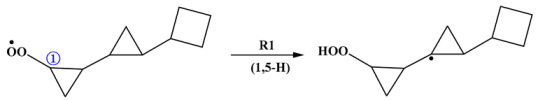
Site. 2	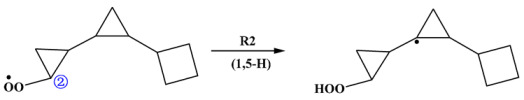	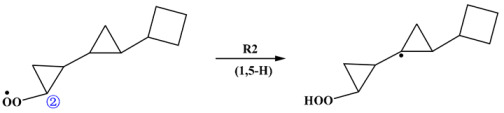
Site. 3	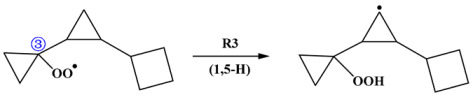	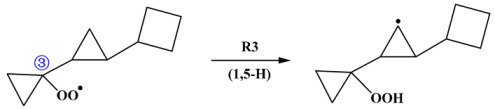
Site. 4	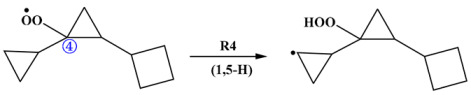	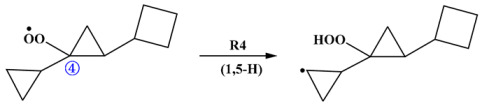
Site. 4	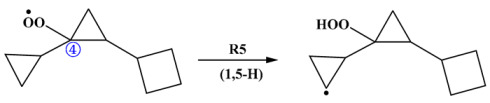	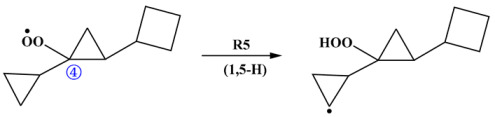
Site. 5	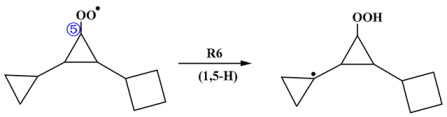	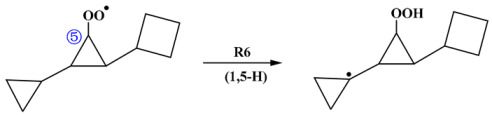
Site. 5	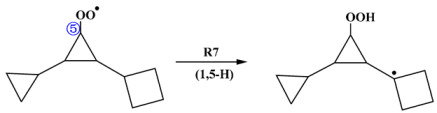	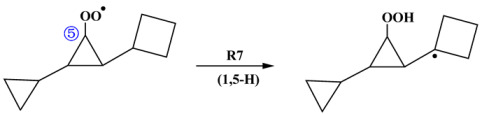
Site. 6	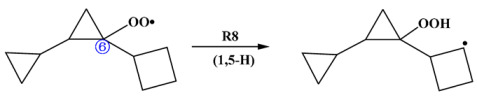	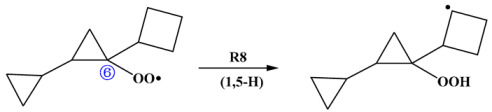
Site. 6	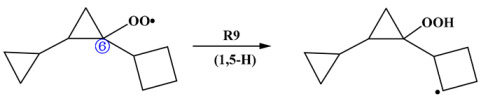	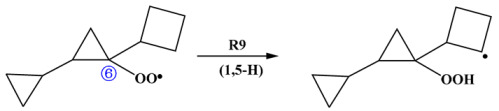
Site. 7	No 1,5 H-migration	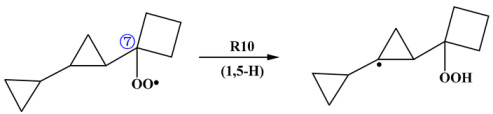
Site. 7	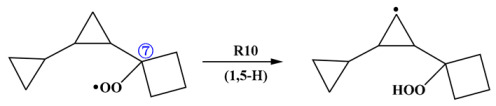	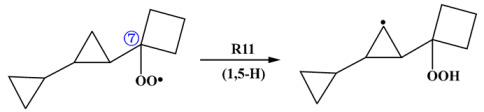
Site. 7	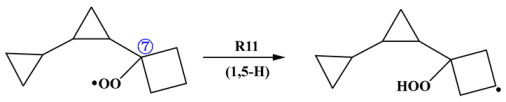	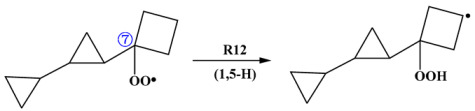
Site. 8	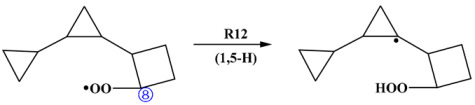	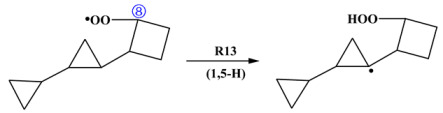
Site. 8	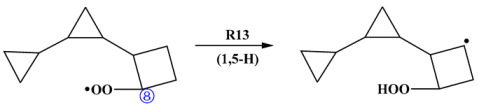	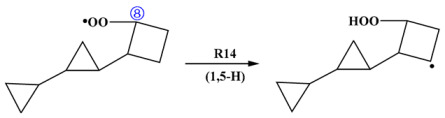
Site. 9	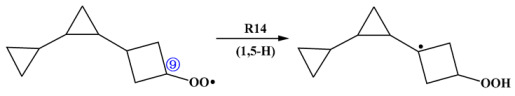	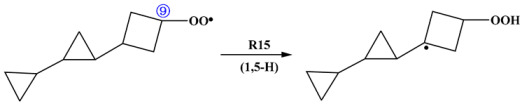
Site. 10	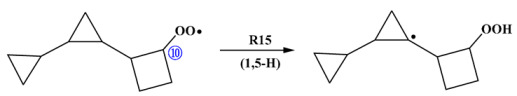	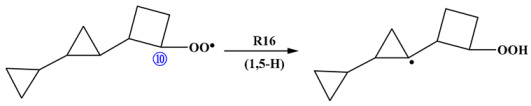
Site. 10	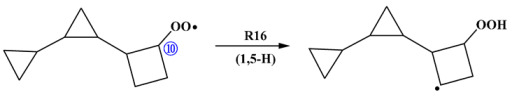	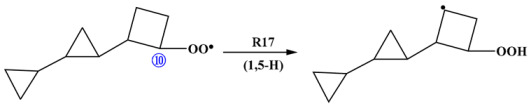

^a^ Sites 1–10 denote the carbon sites defined in Figure 1. ^b^ All blue symbols mark the carbon atom positions defined in Figure 1.

**Table 2:** Energy barriers for 1,5 H-migration of peroxy radicals of *cis*-CP-CB-CP and *trans*-CP-CB-CP (unit: kcal mol^−1^).

Reaction Name	Reaction Process ^a^	Energy Barrier
*cis*-R1	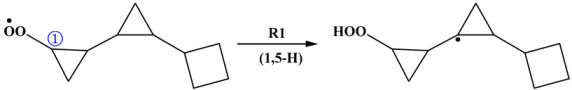	22.16
*cis*-R2	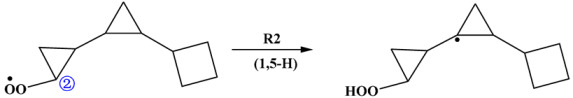	22.99
*cis*-R3	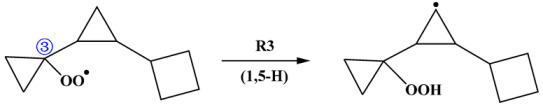	25.10
*cis*-R4	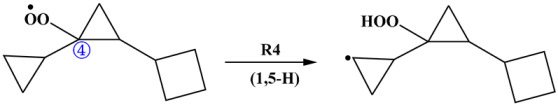	24.19
*cis*-R5	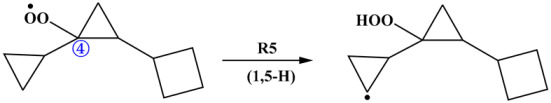	24.86
*cis*-R6	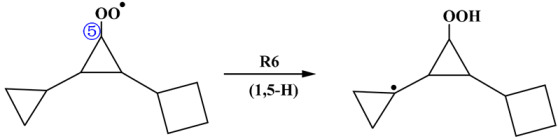	25.90
*cis*-R7	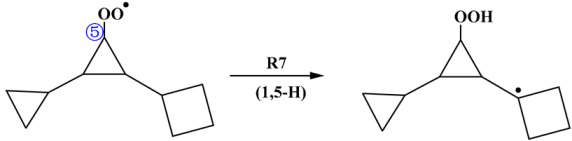	20.06
*cis*-R8	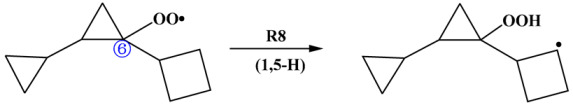	20.18
*cis*-R9	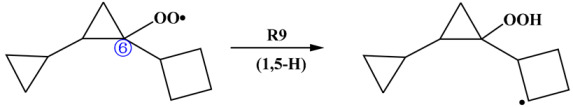	20.36
*cis*-R10	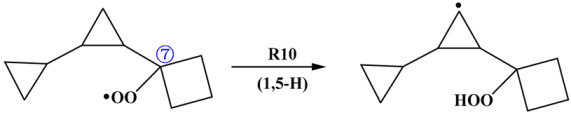	24.30
*cis*-R11	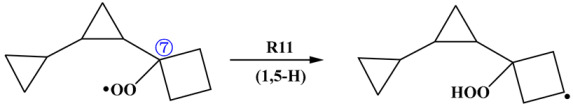	31.04
*cis*-R12	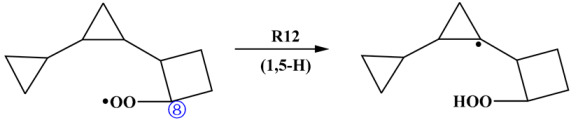	21.46
*cis*-R13	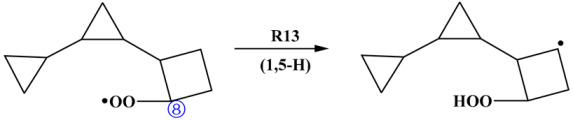	31.13
*cis*-R14	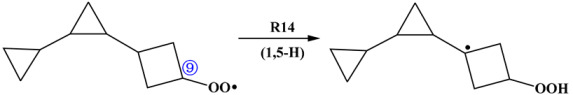	29.37
*cis*-R15	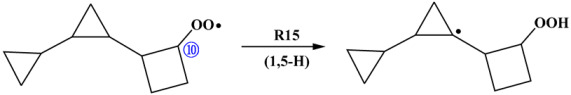	21.05
*cis*-R16	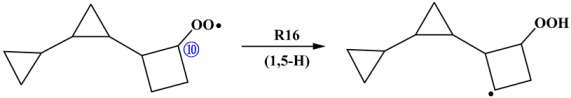	30.69
*trans*-R1	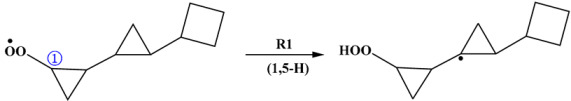	22.94
*trans*-R2	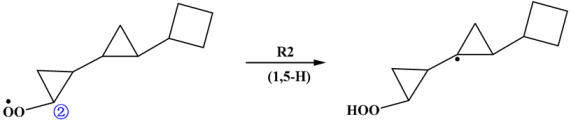	23.59
*trans*-R3	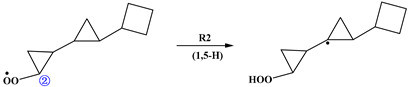	24.50
*trans*-R4	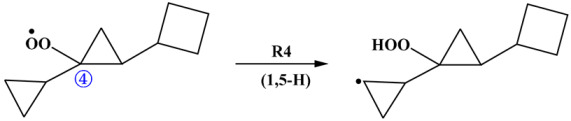	25.07
*trans*-R5	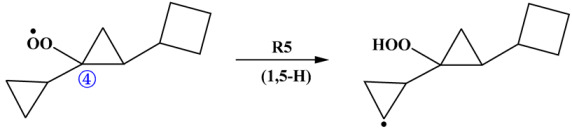	24.25
*trans*-R6	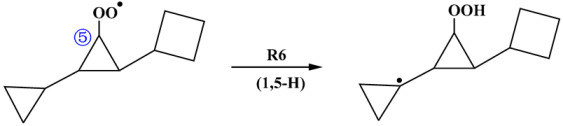	24.34
*trans*-R7	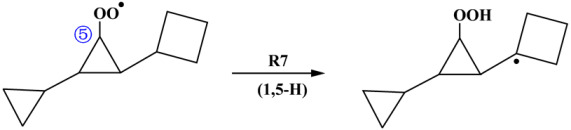	18.25
*trans*-R8	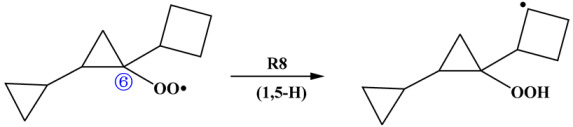	19.92
*trans*-R9	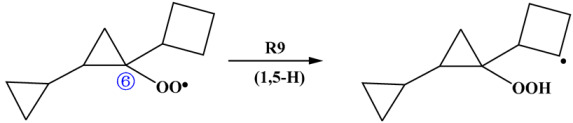	19.48
*trans*-R10	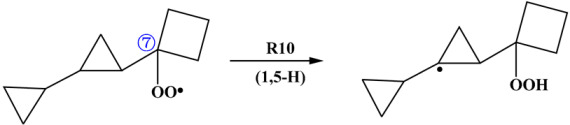	18.85
*trans*-R11	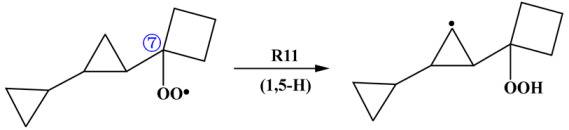	22.53
*trans*-R12	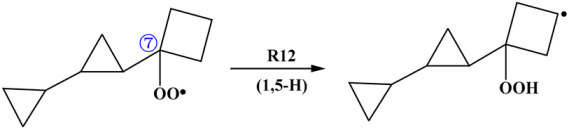	30.99
*trans*-R13	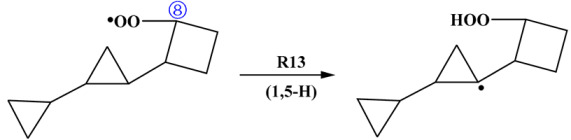	22.12
*trans*-R14	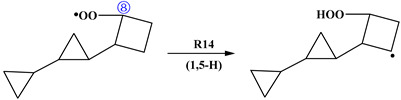	30.77
*trans*-R15	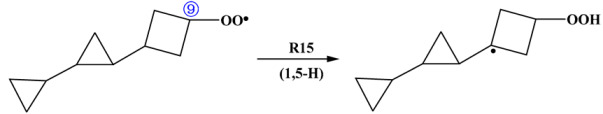	29.55
*trans*-R16	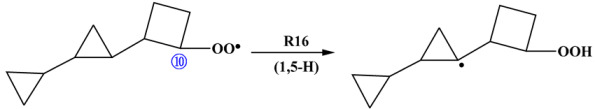	21.99
*trans*-R17	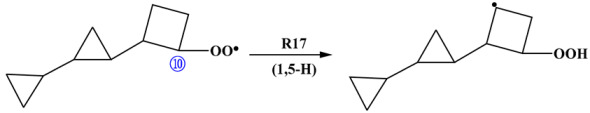	30.92

^a^ All blue symbols mark the carbon atom positions defined in Figure 1.

**Table 3:** Modified Arrhenius parameters for 1,5 H-migration reactions of peroxy radicals of *cis*-CP-CB-CP and *trans*-CP-CB-CP over the temperature range of 500–2500 K.

	Modified Arrhenius Parameters		
Reaction	*A* (s^−1^)	*n*	*E* (kcal mol^−1^)
*cis*-R1	8.19 × 10^7^	1.14	19.85
*cis*-R2	2.37 × 10^8^	1.10	20.87
*cis*-R3	3.00 × 10^9^	0.78	25.00
*cis*-R4	1.45 × 10^10^	0.70	24.03
*cis*-R5	1.17 × 10^10^	0.70	24.66
*cis*-R6	4.40 × 10^7^	1.24	23.35
*cis*-R7	2.93 × 10^7^	1.30	17.31
*cis*-R8	4.14 × 10^9^	0.93	18.64
*cis*-R9	7.91 × 10^9^	0.94	18.95
*cis*-R10	8.31 × 10^9^	0.83	23.42
*cis*-R11	1.53 × 10^5^	2.03	25.66
*cis*-R12	7.72 × 10^7^	1.26	18.78
*cis*-R13	8.60 × 10^6^	1.50	27.62
*cis*-R14	5.53 × 10^6^	1.56	25.75
*cis*-R15	1.60 × 10^9^	0.90	19.68
*cis*-R16	1.78 × 10^7^	1.50	27.28
*trans*-R1	2.31 × 10^8^	1.10	20.79
*trans*-R2	1.42 × 10^8^	1.15	21.26
*trans*-R3	2.99 × 10^17^	−3.92	14.23
*trans*-R4	7.18 × 10^8^	1.04	23.25
*trans*-R5	1.02 × 10^4^	2.33	15.99
*trans*-R6	1.20 × 10^8^	1.14	21.94
*trans*-R7	5.20 × 10^7^	1.26	15.34
*trans*-R8	1.21 × 10^9^	1.33	18.02
*trans*-R9	3.20 × 10^9^	1.00	18.02
*trans*-R10	2.22 × 10^10^	0.61	18.70
*trans*-R11	1.34 × 10^10^	0.67	22.33
*trans*-R12	1.34 × 10^10^	0.67	22.33
*trans*-R13	9.58 × 10^6^	1.54	27.20
*trans*-R14	1.05 × 10^7^	1.51	27.13
*trans*-R15	2.02 × 10^7^	1.53	25.93
*trans*-R16	9.93 × 10^9^	0.88	20.89
*trans*-R17	4.47 × 10^8^	1.24	28.66

**Table 4:** Comparison of energy barriers at 0 K (kcal mol^−1^) by CBS-QB3 method and CCSD(T)/cc-pVTZ method.

No.	Reaction Equation ^a^	*E*_1_	*E*_2_	$∆(E_{1}-E_{2})$
		CBS-QB3	CCSD(T)/cc-pVTZ	
*cis*-R5	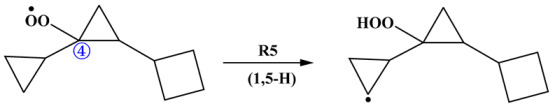	24.86	26.70	−1.84
*trans*-R5	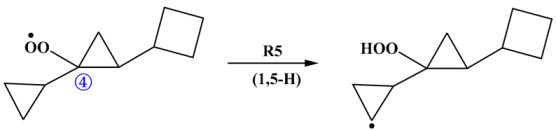	24.25	26.05	−1.80

^a^ Blue symbols mark the carbon atom positions defined in Figure 1.

## Data Availability

Data are contained within the article and Supplementary Materials.

## Supplementary Materials

The following supporting information can be downloaded at: https://www.mdpi.com/article/10.3390/molecules31132302/s1.
