# Supplementary material for: Study on the Intramolecular H-Migration Kinetics of Strained Polycyclic Hydrocarbons with Distinct Cis and Trans Configurations
Source: Molecules. 2026 Jul 1;31(13):2302. doi: 10.3390/molecules31132302 (PMC13362817; doi:10.3390/molecules31132302)
Supplement: Supplementary file 1 [file molecules-31-02302-s001.zip › molecules-4352670-supplementary.pdf]

# Study on the Intramolecular H-Migration Kinetics of Strained Polycyclic Hydrocarbons with Distinct *cis* and *trans* Configurations

Xiaoxia Yao[1], Ying Xuan[1], Junjiang Guo[2], Mingxia Liu[3], Zerong Li[4*], Zhian Li[1*]

[1]*College of Aviation Maintenance Industry, Chengdu Aeronautic Polytechnic University, Chengdu 610100, PR China*

[2]*School of Chemical Engineering, Guizhou Institute of Technology, Guiyang 550003, PR China*

[3]*School of Physics and New Energy, Chongqing University of Technology, Chongqing 401135, PR China*

[4]*College of Chemistry, Sichuan University, Chengdu 610064, PR China*

## 1. Energies of the reactants structures

**Table S1.** Energies of reactant structures bearing peroxyl radical (OO•) moieties attached to three-membered and four-membered rings (Units: Hartree, HF).

| Reactant of reaction | Molecular structure | Electronic energy |
|---|---|---|
| *cis*-R1 | 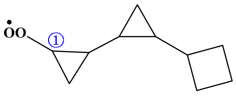 | -539.34352 |
| *cis*-R2 | 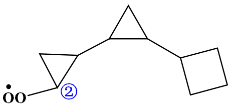 | -539.344107 |
| *cis*-R3 | 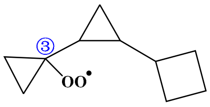 | -539.347644 |
| *cis*-R6 | 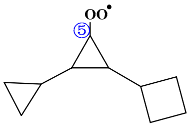 | -539.344195 |
| *cis*-R10 | 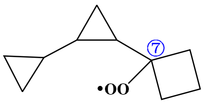 | -539.353544 |
| *cis*-R12 | 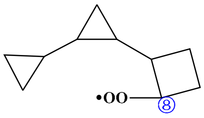 | -539.350973 |
| *cis*-R14 | 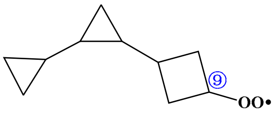 | -539.352435 |

| | | |
|---|---|---|
| *cis*-R15 | 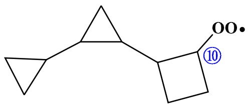 | -539.350298 |

## 2. Tunneling correction factors of reactions over the temperature range of 500-2500 K

Table S2. Tunneling correction factors of reaction *cis*-R1 over the temperature range of 500-2500 K. $k_{total} = \kappa \cdot k_{TST}$, where $k_{total}$ denotes the total high-pressure-limit rate constant including quantum tunneling corrections; $k_{TST}$ represents the rate constant predicted by conventional transition-state theory; and $\kappa$ is the tunneling correction factor.

| ***T*** **(K)** | $k_{total}$ | $k_{\mathrm{TST}}$ | $\kappa$ |
|---|---|---|---|
| 500 | 2.15E+02 | 2.15E+02 | 3.00 |
| 600 | 6.90E+03 | 6.90E+03 | 2.16 |
| 700 | 8.74E+04 | 8.74E+04 | 1.77 |
| 800 | 6.08E+05 | 6.08E+05 | 1.56 |
| 900 | 2.81E+06 | 2.81E+06 | 1.43 |
| 1000 | 9.73E+06 | 9.73E+06 | 1.34 |
| 1100 | 2.72E+07 | 2.72E+07 | 1.28 |
| 1200 | 6.46E+07 | 6.46E+07 | 1.23 |
| 1300 | 1.35E+08 | 1.35E+08 | 1.21 |
| 1400 | 2.55E+08 | 2.55E+08 | 1.17 |
| 1500 | 4.46E+08 | 4.46E+08 | 1.15 |
| 1600 | 7.28E+08 | 7.28E+08 | 1.13 |
| 1700 | 1.13E+09 | 1.13E+09 | 1.13 |
| 1800 | 1.66E+09 | 1.66E+09 | 1.11 |
| 1900 | 2.35E+09 | 2.35E+09 | 1.09 |
| 2000 | 3.22E+09 | 3.22E+09 | 1.08 |
| 2100 | 4.30E+09 | 4.30E+09 | 1.08 |
| 2200 | 5.58E+09 | 5.58E+09 | 1.07 |
| 2300 | 7.10E+09 | 7.10E+09 | 1.07 |
| 2400 | 8.85E+09 | 8.85E+09 | 1.05 |
| 2500 | 1.12E+10 | 1.08E+10 | 1.05 |

Table S3. Tunneling correction factors of reaction *trans*-R1 over the temperature range of 500-2500 K. $k_{total} = \kappa \cdot k_{TST}$, where $k_{total}$ denotes the total high-pressure-limit rate constant including quantum tunneling corrections; $k_{TST}$ represents the rate constant predicted by conventional transition-state theory; and $\kappa$ is the tunneling correction factor.

| ***T*** **(K)** | $k_{total}$ | $k_{\mathrm{TST}}$ | $\kappa$ |
|---|---|---|---|
| 500 | 1.84E+02 | 6.46E+01 | 2.85 |
| 600 | 6.90E+03 | 3.29E+03 | 2.10 |
| 700 | 9.71E+04 | 5.58E+04 | 1.74 |
| 800 | 7.30E+05 | 4.74E+05 | 1.54 |
| 900 | 3.58E+06 | 2.50E+06 | 1.43 |
| 1000 | 1.30E+07 | 9.80E+06 | 1.33 |
| 1100 | 3.70E+07 | 2.97E+07 | 1.25 |

| | | | |
|---|---|---|---|
| 1200 | 9.20E+07 | 7.54E+07 | 1.22 |
| 1300 | 2.30E+08 | 1.90E+08 | 1.21 |
| 1400 | 3.80E+08 | 3.29E+08 | 1.16 |
| 1500 | 6.80E+08 | 5.97E+08 | 1.14 |
| 1600 | 1.13E+09 | 1.00E+09 | 1.13 |
| 1700 | 1.78E+09 | 1.60E+09 | 1.11 |
| 1800 | 2.66E+09 | 2.40E+09 | 1.11 |
| 1900 | 3.80E+09 | 3.50E+09 | 1.09 |
| 2000 | 5.30E+09 | 4.89E+09 | 1.08 |
| 2100 | 7.10E+09 | 6.62E+09 | 1.07 |
| 2200 | 9.35E+09 | 8.70E+09 | 1.07 |
| 2300 | 1.19E+10 | 1.11E+10 | 1.07 |
| 2400 | 1.50E+10 | 1.40E+10 | 1.07 |
| 2500 | 1.85E+10 | 1.76E+10 | 1.05 |

**3. Cartesian coordinates for all reactants, transition states and products**

cis-R1-R

| | | | |
|---|---|---|---|
| C | 0.46324500 | 0.91520700 | -0.26980400 |
| C | -1.02196500 | 1.06270300 | -0.48511100 |
| C | -0.26371100 | 2.10644700 | 0.29268700 |
| H | 1.08681200 | 1.10382200 | -1.13564800 |
| H | -1.31303600 | 1.33971200 | -1.49457400 |
| H | -0.08996400 | 3.07514200 | -0.15634900 |
| H | -0.42171900 | 2.12479100 | 1.36541100 |
| C | 0.98732800 | -0.14810500 | 0.64513700 |
| C | 1.48603400 | -1.45145800 | 0.06296300 |
| C | 2.44421300 | -0.47443200 | 0.66351300 |
| H | 0.50762500 | -0.20674700 | 1.61538000 |
| H | 1.49176300 | -1.51916500 | -1.01842800 |
| H | 1.31194300 | -2.37976600 | 0.59031800 |
| H | 2.97124900 | -0.69490400 | 1.58228800 |
| C | -1.99574900 | 0.14405800 | 0.19508700 |
| C | -3.51126200 | 0.44615500 | 0.08922600 |
| C | -2.28434600 | -1.23631000 | -0.44478800 |
| H | -1.73097100 | 0.00996700 | 1.24830500 |
| C | -3.75879000 | -1.07756700 | -0.00693000 |
| H | -3.73372100 | 0.93967300 | -0.86056900 |
| H | -3.98427900 | 1.00159800 | 0.89946400 |
| H | -2.18744500 | -1.17533900 | -1.53226500 |
| H | -1.71968200 | -2.09715700 | -0.08438700 |
| H | -4.53581700 | -1.42798700 | -0.68577000 |
| H | -3.92606000 | -1.51257900 | 0.98031200 |
| O | 3.24575600 | 0.27644600 | -0.23899500 |
| O | 4.37965800 | -0.31410200 | -0.48167600 |

cis-R1-TS

| | | | |
|---|---|---|---|
| C | -0.61907100 | 0.81476400 | -0.08182800 |
| C | 0.82701500 | 0.85318600 | 0.25920900 |
| C | 0.14082400 | 2.03961100 | -0.41343100 |
| H | -1.45833800 | 0.70391500 | 1.00605100 |
| H | 1.08619100 | 1.01315200 | 1.30402200 |
| H | -0.00411600 | 2.93093400 | 0.18576000 |
| H | 0.36204200 | 2.22616600 | -1.46090100 |
| C | -1.29967700 | -0.08720300 | -1.02439700 |
| C | -1.58470000 | -1.50771700 | -0.56716000 |
| C | -2.70332900 | -0.53187300 | -0.60688600 |
| H | -1.19155300 | 0.11844400 | -2.08484400 |
| H | -1.19355000 | -1.76811800 | 0.40767400 |
| H | -1.59436100 | -2.29913200 | -1.30505800 |
| H | -3.47239200 | -0.61429900 | -1.36292100 |
| O | -3.26226800 | 0.05893500 | 0.52375000 |
| O | -2.32956800 | 0.14993900 | 1.54204400 |
| C | 1.79310500 | -0.00983700 | -0.51002300 |
| C | 3.27360100 | 0.41239000 | -0.31159600 |
| C | 2.12701100 | -1.33961000 | 0.21785900 |
| H | 1.45285400 | -0.10295300 | -1.54459800 |
| C | 3.39824800 | -0.66736900 | 0.79002600 |
| H | 3.45474900 | 1.45184900 | -0.03271100 |
| H | 3.87899700 | 0.16614400 | -1.18569000 |
| H | 1.38497600 | -1.70986400 | 0.92721100 |
| H | 2.37382500 | -2.13313800 | -0.48957000 |
| H | 3.22390100 | -0.25358800 | 1.78445500 |
| H | 4.31329700 | -1.25855400 | 0.81412300 |

cis-R1-P

| | | | |
|---|---|---|---|
| C | 0.37273200 | 1.32274900 | 0.23888100 |
| C | -0.59070500 | 0.45063200 | -0.43732000 |
| C | -0.11493000 | 1.76272600 | -1.07437300 |
| H | -0.20743400 | -0.46671500 | -0.88081400 |
| H | 0.56851900 | 1.70287200 | -1.91713400 |
| H | -0.84335600 | 2.56692600 | -1.13222400 |
| C | 1.69755000 | 1.14737900 | 0.83062000 |
| C | 2.02780100 | -0.12828500 | 1.56376600 |
| C | 2.69512600 | 0.15221100 | 0.25886500 |
| H | 2.16701200 | 2.05888300 | 1.18539900 |
| H | 1.25703900 | -0.88934100 | 1.53747100 |
| H | 2.62716300 | -0.08713400 | 2.46309300 |
| H | 3.74591700 | 0.42591200 | 0.24662500 |
| C | -2.00720900 | 0.36079200 | 0.06573700 |
| C | -3.10695400 | -0.17489600 | -0.88293600 |

| | | | |
|---|---|---|---|
| C | -2.37157700 | -0.78187800 | 1.04477500 |
| H | -2.31177500 | 1.32288800 | 0.49048400 |
| C | -3.74207400 | -0.88489200 | 0.33557600 |
| H | -2.68231600 | -0.89962000 | -1.58277000 |
| H | -3.70752800 | 0.54755000 | -1.43652700 |
| H | -1.75840700 | -1.66438500 | 0.84142500 |
| H | -2.35056800 | -0.56879900 | 2.11402700 |
| H | -4.16755300 | -1.87705700 | 0.18555000 |
| H | -4.48485300 | -0.24798400 | 0.81978600 |
| O | 2.26255800 | -0.52725000 | -0.87219900 |
| O | 2.70257100 | -1.88764500 | -0.76005700 |
| H | 3.26854200 | -1.96405700 | -1.53789600 |

cis-R2-R

| | | | |
|---|---|---|---|
| C | 0.54051900 | 1.43793300 | -0.48766400 |
| C | -0.90128200 | 1.07672100 | -0.73477800 |
| C | -0.53449000 | 2.48052300 | -0.33371400 |
| H | 1.16184900 | 1.51470200 | -1.37238500 |
| H | -1.16534800 | 0.93092400 | -1.77833100 |
| H | -0.59506300 | 3.26967800 | -1.07125400 |
| H | -0.78501700 | 2.78346600 | 0.67698000 |
| C | 1.25015300 | 0.91053900 | 0.72368600 |
| C | 1.88909600 | -0.43885100 | 0.66860200 |
| C | 2.75457900 | 0.77915600 | 0.71034900 |
| H | 0.79992500 | 1.15947900 | 1.67775100 |
| H | 1.85254200 | -1.13733600 | 1.49416100 |
| H | 3.31619600 | 0.96748100 | 1.61527600 |
| H | 3.25507200 | 1.03911900 | -0.21447600 |
| C | -1.62932400 | 0.16496600 | 0.21059600 |
| C | -3.15813800 | -0.01754800 | 0.04347400 |
| C | -1.48312200 | -1.36620200 | 0.02509000 |
| H | -1.40848400 | 0.43084000 | 1.24929900 |
| C | -2.97274000 | -1.50747900 | 0.41418000 |
| H | -3.43989500 | 0.09258100 | -1.00709100 |
| H | -3.82052800 | 0.59455700 | 0.65643800 |
| H | -1.31988100 | -1.60656000 | -1.02852300 |
| H | -0.73555800 | -1.88835300 | 0.62353200 |
| H | -3.57302200 | -2.24507800 | -0.11786800 |
| H | -3.08775100 | -1.66365600 | 1.48864400 |
| O | 1.76917200 | -1.09087500 | -0.58897500 |
| O | 2.60751000 | -2.07917500 | -0.70240900 |

cis-R2-TS

| | | | |
|---|---|---|---|
| C | 0.55038000 | 0.62492800 | -0.11640600 |
| C | -0.89279400 | 0.73869300 | -0.40576900 |

| | | | |
|---|---|---|---|
| C | -0.09025900 | 1.96094200 | 0.03245300 |
| H | 1.51302500 | 0.58014200 | -1.10256000 |
| H | -1.18117200 | 0.73811500 | -1.45547800 |
| H | 0.10168100 | 2.72726100 | -0.70931000 |
| H | -0.24089600 | 2.32914200 | 1.04323100 |
| C | 1.19725900 | -0.20681300 | 0.91074000 |
| C | 2.42605900 | -0.98751300 | 0.43889400 |
| C | 2.59989800 | 0.17143400 | 1.35154700 |
| H | 0.55387500 | -0.75566700 | 1.59194100 |
| H | 2.57718000 | -1.99503000 | 0.80227300 |
| H | 2.90202300 | -0.04354600 | 2.36812300 |
| H | 2.98898300 | 1.08488300 | 0.92054500 |
| O | 2.72937200 | -0.96259500 | -0.92055400 |
| O | 2.59709000 | 0.31757300 | -1.42904700 |
| C | -1.90006800 | 0.11416300 | 0.52695000 |
| C | -3.36411800 | 0.48530600 | 0.17385200 |
| C | -2.23447100 | -1.35542300 | 0.15463000 |
| H | -1.59753800 | 0.29246200 | 1.56234000 |
| C | -3.46935200 | -0.83789200 | -0.62215300 |
| H | -3.51762900 | 1.42179000 | -0.36503500 |
| H | -4.00332900 | 0.47276200 | 1.05825600 |
| H | -1.47347400 | -1.90435500 | -0.40319500 |
| H | -2.52700800 | -1.93558400 | 1.03187300 |
| H | -3.24779400 | -0.69290400 | -1.68058500 |
| H | -4.39482600 | -1.40624200 | -0.53404200 |

cis-R2-P

| | | | |
|---|---|---|---|
| C | 0.64266500 | 1.58812700 | -0.36879200 |
| C | -0.71608400 | 1.11946300 | -0.67761900 |
| C | -0.46297600 | 2.51320100 | -0.09533900 |
| H | -1.00578600 | 1.11578500 | -1.72833100 |
| H | -0.61706200 | 3.36905700 | -0.74526100 |
| H | -0.75202600 | 2.67651000 | 0.94164900 |
| C | 1.76698800 | 1.14550500 | 0.45447000 |
| C | 2.09399200 | -0.31053000 | 0.68042600 |
| C | 2.97241900 | 0.49026200 | -0.21600200 |
| H | 2.00780500 | 1.79215900 | 1.29146300 |
| H | 2.45869500 | -0.61308700 | 1.65641600 |
| H | 3.96372900 | 0.75839100 | 0.12320400 |
| H | 2.86217300 | 0.31716400 | -1.27955100 |
| C | -1.44970900 | 0.10356300 | 0.16046700 |
| C | -2.97507900 | 0.31194500 | 0.34500900 |
| C | -1.76860300 | -1.29268800 | -0.42657500 |
| H | -0.96082300 | -0.00730400 | 1.13406800 |
| C | -3.14712500 | -1.22219900 | 0.26995900 |

| | | | |
|---|---|---|---|
| H | -3.39798200 | 0.79975100 | -0.53777000 |
| H | -3.31775100 | 0.83413300 | 1.23929200 |
| H | -1.87488400 | -1.23121500 | -1.51368600 |
| H | -1.09708100 | -2.11098400 | -0.17406500 |
| H | -4.01353600 | -1.61238500 | -0.26417300 |
| H | -3.10890400 | -1.67325600 | 1.26333700 |
| O | 1.20442400 | -1.20625200 | 0.09996000 |
| O | 1.92590000 | -2.39480200 | -0.22374400 |
| H | 2.07191000 | -2.28617800 | -1.17234900 |

cis-R3-R

| | | | |
|---|---|---|---|
| C | -0.57129800 | 0.76247900 | 1.06720500 |
| C | 0.90399000 | 0.79543500 | 0.72235700 |
| C | 0.04488500 | 1.98090900 | 0.41896900 |
| H | -0.81025700 | 0.87591300 | 2.11806100 |
| H | 1.56578900 | 0.88689800 | 1.57959900 |
| H | 0.13064500 | 2.87379800 | 1.02319700 |
| H | -0.19995800 | 2.15413400 | -0.62158000 |
| C | -1.55195800 | -0.07929100 | 0.31458400 |
| C | -1.33382200 | -1.52205900 | -0.01219400 |
| C | -2.37254200 | -1.12119100 | 1.00200400 |
| H | -0.40710400 | -1.96121600 | 0.33132700 |
| H | -1.70603500 | -1.85823500 | -0.97151200 |
| H | -3.40536900 | -1.19671600 | 0.68759400 |
| H | -2.17220400 | -1.28509800 | 2.05302200 |
| C | 1.46444100 | -0.01290700 | -0.41834000 |
| C | 2.84653200 | 0.51040600 | -0.89413500 |
| C | 2.17220900 | -1.31790900 | 0.03824300 |
| H | 0.70860500 | -0.12429800 | -1.19904500 |
| C | 3.52454500 | -0.56734500 | -0.01539900 |
| H | 3.07179200 | 1.55687200 | -0.68185200 |
| H | 3.00222600 | 0.31994000 | -1.95722300 |
| H | 1.86692400 | -1.73232000 | 1.00172800 |
| H | 2.11003400 | -2.09857600 | -0.72249200 |
| H | 3.80849600 | -0.17478200 | 0.96231600 |
| H | 4.37667000 | -1.09850900 | -0.43855300 |
| O | -2.23124000 | 0.71713100 | -0.68290800 |
| O | -3.10652800 | 0.06924900 | -1.38013600 |

cis-R3-TS

| | | | |
|---|---|---|---|
| C | 0.78625400 | -0.29588900 | 1.20373900 |
| C | -0.69473000 | -0.54943300 | 0.90244600 |
| C | 0.28383600 | -1.65556800 | 0.87686400 |
| H | 1.07483600 | -0.11233500 | 2.23401300 |
| H | -1.34866000 | -0.53191400 | 1.77427800 |

| | | | |
|---|---|---|---|
| H | 0.32456900 | -2.52855500 | 1.51546300 |
| H | 0.87003000 | -1.82569300 | -0.38306600 |
| C | 1.69932600 | 0.32968400 | 0.17168400 |
| C | 1.36953100 | 1.57549400 | -0.58717500 |
| C | 2.48323500 | 1.56839900 | 0.43319300 |
| H | 0.44975000 | 2.08718800 | -0.33601500 |
| H | 1.65105700 | 1.58680300 | -1.63213200 |
| H | 3.50038700 | 1.57975000 | 0.06475500 |
| H | 2.31963600 | 2.06694100 | 1.37983800 |
| C | -1.35992200 | -0.04055400 | -0.35533700 |
| C | -2.73374300 | -0.72393300 | -0.59396300 |
| C | -2.11824500 | 1.29588500 | -0.13628100 |
| H | -0.66161100 | -0.07418500 | -1.19037700 |
| C | -3.42273800 | 0.48965800 | 0.07413300 |
| H | -2.87918400 | -1.70598000 | -0.14064000 |
| H | -2.96569500 | -0.78620400 | -1.65823600 |
| H | -1.77743300 | 1.92642300 | 0.68815400 |
| H | -2.15208300 | 1.89162200 | -1.05054100 |
| H | -3.62549500 | 0.31300600 | 1.13172900 |
| H | -4.32922000 | 0.87273600 | -0.39366200 |
| O | 2.43677300 | -0.65018500 | -0.51565800 |
| O | 1.53726400 | -1.44007200 | -1.22676300 |

cis-R3-P

| | | | |
|---|---|---|---|
| C | -1.47450200 | -1.00124000 | 1.17455900 |
| C | -1.53552500 | 0.15486200 | 0.23489700 |
| C | -2.72472900 | -0.13986100 | 1.08740800 |
| H | -1.54836900 | -1.99610300 | 0.75702800 |
| H | -0.83019600 | -0.89240000 | 2.03819400 |
| H | -2.94883600 | 0.54396600 | 1.89632800 |
| H | -3.58169800 | -0.59854800 | 0.61321500 |
| C | -0.70371200 | 1.37988000 | 0.50758400 |
| C | 0.81644400 | 1.34030700 | 0.31290300 |
| C | -0.04734700 | 2.11578300 | -0.58337100 |
| H | -1.07241300 | 1.96340700 | 1.34764100 |
| H | 1.40092200 | 1.89914000 | 1.04480600 |
| H | -0.08011100 | 3.15116900 | -0.88342900 |
| C | 1.48657400 | 0.07507300 | -0.16054400 |
| C | 2.92658800 | 0.18450400 | -0.71861000 |
| C | 2.02778000 | -0.92314700 | 0.89229900 |
| H | 0.84443800 | -0.44204200 | -0.87738200 |
| C | 3.24090400 | -1.16658000 | -0.03543500 |
| H | 3.46478800 | 1.00340100 | -0.23301400 |
| H | 3.05112600 | 0.26508700 | -1.79859100 |
| H | 2.32686800 | -0.39732500 | 1.80387100 |

| | | | |
|---|---|---|---|
| H | 1.40164800 | -1.77542500 | 1.15796100 |
| H | 4.22090800 | -1.29132500 | 0.42502400 |
| H | 3.05552000 | -2.00252700 | -0.71222600 |
| O | -1.58328600 | -0.05417400 | -1.16803000 |
| O | -2.26295400 | -1.27057600 | -1.44284100 |
| H | -3.00952300 | -0.94995600 | -1.96259700 |

cis-R4-R

| | | | |
|---|---|---|---|
| C | -0.97014600 | -0.40270300 | 0.26286600 |
| C | 0.43176200 | -0.91880600 | 0.15987500 |
| C | -0.47152600 | -1.36751900 | 1.28761500 |
| H | 0.55916900 | -1.65722300 | -0.62735700 |
| H | -0.86374800 | -2.37477200 | 1.23331200 |
| H | -0.28993600 | -0.97165200 | 2.28007700 |
| C | -1.28416300 | 1.02442300 | 0.57153400 |
| C | -1.69054000 | 1.96707200 | -0.52556700 |
| C | -2.69615900 | 1.53478000 | 0.50912200 |
| H | -0.66057100 | 1.43365200 | 1.35652700 |
| H | -1.79739100 | 1.54993900 | -1.51867200 |
| H | -1.31507300 | 2.98096200 | -0.48756500 |
| H | -3.00215100 | 2.25328500 | 1.25752800 |
| H | -3.46359300 | 0.84188600 | 0.19168400 |
| C | 1.59825800 | 0.00022700 | 0.38062000 |
| C | 3.02962700 | -0.59166200 | 0.35221600 |
| C | 2.02442600 | 0.94203900 | -0.77151400 |
| H | 1.46646000 | 0.57873600 | 1.30002400 |
| C | 3.49513400 | 0.70033800 | -0.35940100 |
| H | 3.07256200 | -1.44887500 | -0.32441300 |
| H | 3.49393900 | -0.86113900 | 1.30096900 |
| H | 1.80361100 | 0.48044400 | -1.73758600 |
| H | 1.63721800 | 1.96148500 | -0.76694300 |
| H | 4.23949800 | 0.60780200 | -1.14956700 |
| H | 3.83140200 | 1.44831400 | 0.36113000 |
| O | -1.91970900 | -0.88881200 | -0.70122700 |
| O | -1.76921900 | -2.13018400 | -1.03294200 |

cis-R4-TS

| | | | |
|---|---|---|---|
| C | -0.84400100 | -0.51266900 | 0.06426800 |
| C | 0.55877400 | -0.97507600 | -0.14595900 |
| C | -0.34291500 | -1.68786300 | 0.84292700 |
| H | 0.71081300 | -1.49183700 | -1.08958800 |
| H | -0.74501900 | -2.65323400 | 0.56590700 |
| H | -0.14467700 | -1.54225100 | 1.89857100 |
| C | -1.21296400 | 0.78882700 | 0.73407600 |
| C | -1.65752300 | 1.91427200 | -0.18449500 |

| | | | |
|---|---|---|---|
| C | -2.63873200 | 1.16578700 | 0.63272400 |
| H | -0.67928900 | 1.00964200 | 1.65156100 |
| H | -1.59815300 | 1.68901100 | -1.24498800 |
| H | -1.44694300 | 2.94320100 | 0.08388600 |
| H | -3.26610900 | 1.55799100 | 1.42206500 |
| H | -3.16064400 | 0.07292100 | -0.05395200 |
| O | -1.68162000 | -0.77481100 | -1.02870900 |
| O | -2.99088300 | -0.93385700 | -0.57106100 |
| C | 1.71252500 | -0.12870000 | 0.30808400 |
| C | 3.14627100 | -0.70695600 | 0.21943700 |
| C | 2.18218600 | 1.03029800 | -0.60406700 |
| H | 1.53646400 | 0.24864100 | 1.32026600 |
| C | 3.63527500 | 0.70271400 | -0.18830800 |
| H | 3.22239400 | -1.40547300 | -0.61783100 |
| H | 3.57145800 | -1.16929600 | 1.11043400 |
| H | 2.00564000 | 0.78050000 | -1.65362700 |
| H | 1.79101300 | 2.02853600 | -0.40427000 |
| H | 4.41319600 | 0.77352200 | -0.94784700 |
| H | 3.93649600 | 1.28366200 | 0.68545400 |

cis-R4-P

| | | | |
|---|---|---|---|
| C | -1.30677400 | 2.19549800 | -0.37642200 |
| C | -1.24007800 | 1.11677900 | 0.70341400 |
| C | -2.49004700 | 1.83204400 | 0.41465300 |
| H | -0.79114600 | 3.12664700 | -0.16358700 |
| H | -1.26880800 | 1.87493300 | -1.41369700 |
| H | -0.71080800 | 1.40046100 | 1.60831900 |
| H | -3.44611800 | 1.45206300 | 0.09259600 |
| C | -1.05271800 | -0.32381800 | 0.32704800 |
| C | 0.32030900 | -0.88763600 | 0.15001000 |
| C | -0.55224100 | -1.32022900 | 1.32158800 |
| H | 0.40187900 | -1.63079000 | -0.63675100 |
| H | -0.95287600 | -2.32454500 | 1.28413700 |
| H | -0.30080300 | -0.95450200 | 2.31074000 |
| C | 1.54069500 | -0.03503500 | 0.34263900 |
| C | 2.92846000 | -0.72194000 | 0.39160400 |
| C | 2.05800600 | 0.79813300 | -0.85566700 |
| H | 1.42810900 | 0.61383800 | 1.21639700 |
| C | 3.49877800 | 0.48500900 | -0.38957100 |
| H | 2.92672800 | -1.62334700 | -0.22677100 |
| H | 3.35278000 | -0.95886000 | 1.36770200 |
| H | 1.82748400 | 0.29046700 | -1.79610900 |
| H | 1.74484700 | 1.84025000 | -0.92642800 |
| H | 4.25314200 | 0.29039000 | -1.15152300 |
| H | 3.86957800 | 1.25493400 | 0.28986200 |

| | | | |
|---|---|---|---|
| O | -2.09186100 | -0.66104900 | -0.57972200 |
| O | -1.89490600 | -1.97126300 | -1.08353800 |
| H | -2.66618700 | -2.42627200 | -0.72457500 |

cis-R5-R

| | | | |
|---|---|---|---|
| C | -0.97014600 | -0.40270300 | 0.26286600 |
| C | 0.43176200 | -0.91880600 | 0.15987500 |
| C | -0.47152600 | -1.36751900 | 1.28761500 |
| H | 0.55916900 | -1.65722300 | -0.62735700 |
| H | -0.86374800 | -2.37477200 | 1.23331200 |
| H | -0.28993600 | -0.97165200 | 2.28007700 |
| C | -1.28416300 | 1.02442300 | 0.57153400 |
| C | -1.69054000 | 1.96707200 | -0.52556700 |
| C | -2.69615900 | 1.53478000 | 0.50912200 |
| H | -0.66057100 | 1.43365200 | 1.35652700 |
| H | -1.79739100 | 1.54993900 | -1.51867200 |
| H | -1.31507300 | 2.98096200 | -0.48756500 |
| H | -3.00215100 | 2.25328500 | 1.25752800 |
| H | -3.46359300 | 0.84188600 | 0.19168400 |
| C | 1.59825800 | 0.00022700 | 0.38062000 |
| C | 3.02962700 | -0.59166200 | 0.35221600 |
| C | 2.02442600 | 0.94203900 | -0.77151400 |
| H | 1.46646000 | 0.57873600 | 1.30002400 |
| C | 3.49513400 | 0.70033800 | -0.35940100 |
| H | 3.07256200 | -1.44887500 | -0.32441300 |
| H | 3.49393900 | -0.86113900 | 1.30096900 |
| H | 1.80361100 | 0.48044400 | -1.73758600 |
| H | 1.63721800 | 1.96148500 | -0.76694300 |
| H | 4.23949800 | 0.60780200 | -1.14956700 |
| H | 3.83140200 | 1.44831400 | 0.36113000 |
| O | -1.91970900 | -0.88881200 | -0.70122700 |
| O | -1.76921900 | -2.13018400 | -1.03294200 |

cis-R5-TS

| | | | |
|---|---|---|---|
| C | -0.81545500 | 0.69781800 | -0.03754600 |
| C | 0.66038400 | 0.87511400 | 0.13102100 |
| C | -0.17871400 | 1.96516800 | -0.50229700 |
| H | 0.94661400 | 1.05298300 | 1.16410800 |
| H | -0.38791000 | 2.85513300 | 0.07671200 |
| H | -0.09556900 | 2.10781200 | -1.57336500 |
| C | -1.39792700 | -0.35201100 | -0.95297800 |
| C | -2.13400000 | -1.43838800 | -0.27193200 |
| C | -2.91336000 | -0.42659300 | -1.01980400 |
| H | -0.80320800 | -0.59244100 | -1.82681400 |
| H | -1.97998800 | -1.07053000 | 1.06348500 |

| | | | |
|---|---|---|---|
| H | -2.07023400 | -2.49967000 | -0.47272100 |
| H | -3.39498300 | -0.73072900 | -1.94196300 |
| H | -3.43722600 | 0.33526200 | -0.45093300 |
| O | -1.60632000 | 0.85925500 | 1.10929700 |
| O | -1.57976400 | -0.33063000 | 1.83971400 |
| C | 1.63888000 | 0.06518200 | -0.67980400 |
| C | 3.11649600 | 0.41658800 | -0.36230700 |
| C | 1.89089800 | -1.33822300 | -0.06703900 |
| H | 1.36632500 | 0.08929600 | -1.73754200 |
| C | 3.14269500 | -0.76306000 | 0.63942600 |
| H | 3.31352900 | 1.41935400 | 0.02032600 |
| H | 3.76413600 | 0.22746500 | -1.22001800 |
| H | 1.09316200 | -1.74194100 | 0.55931100 |
| H | 2.16006500 | -2.06990400 | -0.83124800 |
| H | 2.91598900 | -0.43777200 | 1.65578500 |
| H | 4.03859600 | -1.38288600 | 0.66234300 |

cis-R5-P

| | | | |
|---|---|---|---|
| C | 2.65270300 | -1.71834500 | 0.47755900 |
| C | 1.72045600 | -2.00843900 | -0.62285700 |
| C | 1.23269100 | -1.15496300 | 0.46772600 |
| H | 2.85486700 | -2.49066400 | 1.21259600 |
| H | 3.47578800 | -1.03877900 | 0.28439900 |
| H | 1.81472000 | -1.79376200 | -1.67599400 |
| H | 0.56001000 | -1.60744700 | 1.18999400 |
| C | 0.97749100 | 0.30825300 | 0.26003700 |
| C | -0.41771500 | 0.84851900 | 0.21635700 |
| C | 0.50740100 | 1.17050700 | 1.38324700 |
| H | -0.56108600 | 1.67707000 | -0.47121400 |
| H | 0.89719300 | 2.17746000 | 1.43878800 |
| H | 0.31412800 | 0.68260600 | 2.33193500 |
| C | -1.61240500 | -0.04993700 | 0.36547000 |
| C | -3.02564100 | 0.58147500 | 0.42727500 |
| C | -2.08968700 | -0.86138900 | -0.86340300 |
| H | -1.48630400 | -0.72231100 | 1.21929000 |
| C | -3.54370900 | -0.62218000 | -0.39452600 |
| H | -3.05679500 | 1.50088000 | -0.16346500 |
| H | -3.46359300 | 0.77180100 | 1.40738400 |
| H | -1.87785600 | -0.31055400 | -1.78406900 |
| H | -1.73128800 | -1.88575300 | -0.97049100 |
| H | -4.30305700 | -0.43460900 | -1.15326100 |
| H | -3.88471300 | -1.42773600 | 0.25854900 |
| O | 1.96260000 | 0.78067800 | -0.64699700 |
| O | 1.84552700 | 2.18178800 | -0.81399400 |
| H | 1.57346700 | 2.24105500 | -1.73782700 |

cis-R6-R

| | | | |
|---|---|---|---|
| C | -1.67322500 | -2.34288500 | -0.14942700 |
| C | -1.49268300 | -0.85449700 | -0.26937000 |
| C | -2.87520300 | -1.44142400 | -0.23267800 |
| H | -1.43403200 | -2.95716900 | -1.00731200 |
| H | -1.44590100 | -2.79269400 | 0.80928200 |
| H | -1.16265500 | -0.48572100 | -1.23213100 |
| H | -3.45483400 | -1.28886400 | 0.66907900 |
| H | -3.45152000 | -1.43880300 | -1.14777600 |
| C | -1.00262800 | -0.09557600 | 0.92197900 |
| C | 0.43479100 | 0.37696900 | 1.02170200 |
| C | -0.67238000 | 1.36384800 | 0.81837900 |
| H | -1.47483700 | -0.38654300 | 1.85561000 |
| H | 0.86647200 | 0.37749800 | 2.01860500 |
| H | -0.97156700 | 2.06591200 | 1.58577100 |
| C | 1.42019900 | 0.06527600 | -0.07082400 |
| C | 2.81925500 | 0.72561600 | -0.02667900 |
| C | 2.12587900 | -1.31115000 | -0.03453500 |
| H | 0.97837400 | 0.22676000 | -1.05556500 |
| C | 3.40606600 | -0.60868600 | -0.54475900 |
| H | 3.12368200 | 0.91175800 | 1.00686700 |
| H | 2.97619800 | 1.62948300 | -0.61524100 |
| H | 2.24856600 | -1.65003600 | 0.99790800 |
| H | 1.69702200 | -2.11944000 | -0.62756300 |
| H | 4.36423600 | -0.93621700 | -0.14244100 |
| H | 3.45410900 | -0.62035800 | -1.63508900 |
| O | -0.72542100 | 1.94357100 | -0.47765200 |
| O | -1.93129700 | 2.33136600 | -0.78268900 |

cis-R6-TS

| | | | |
|---|---|---|---|
| C | -0.51460600 | 0.37044500 | 0.85742900 |
| C | 0.95162800 | 0.04674800 | 1.11623400 |
| C | 0.46787000 | 1.49314400 | 0.89950000 |
| H | -1.12576900 | 0.35346800 | 1.75834100 |
| H | 1.28365700 | -0.17547700 | 2.12609700 |
| H | 0.45264700 | 2.15657300 | 1.75475800 |
| C | 1.75739700 | -0.50511500 | 0.01909900 |
| H | 1.64762000 | 0.50691300 | -0.91254700 |
| O | 0.99352400 | 2.21571800 | -0.16502200 |
| O | 1.10294600 | 1.45078600 | -1.31291300 |
| C | 2.95066000 | -1.36686400 | 0.13637600 |
| H | 3.80920900 | -1.16155000 | -0.49196500 |
| H | 3.19127500 | -1.76996000 | 1.11467000 |
| C | 1.69536400 | -1.86997500 | -0.56681000 |

| | | | |
|---|---|---|---|
| H | 1.07523000 | -2.60176700 | -0.05980000 |
| H | 1.75032900 | -1.98665200 | -1.64288200 |
| C | -1.24624600 | -0.15013400 | -0.35515800 |
| C | -2.01774100 | -1.45938400 | -0.03425800 |
| C | -2.61646800 | 0.54940200 | -0.56125200 |
| H | -0.59735000 | -0.17853300 | -1.22476000 |
| C | -3.29629900 | -0.61716900 | 0.19594600 |
| H | -1.64679900 | -2.05842000 | 0.80026900 |
| H | -2.10429900 | -2.09552900 | -0.91678400 |
| H | -2.72221000 | 1.55460200 | -0.15042700 |
| H | -2.89603100 | 0.56709800 | -1.61550400 |
| H | -3.44781500 | -0.38605400 | 1.25180100 |
| H | -4.23081800 | -1.00333400 | -0.21041400 |

cis-R6-P

| | | | |
|---|---|---|---|
| C | -0.50690700 | 0.58940900 | 1.04889000 |
| C | 0.82154400 | -0.10764400 | 1.28932700 |
| C | 0.76344700 | 1.32130200 | 0.75206400 |
| H | -1.02711300 | 0.92564600 | 1.94132700 |
| H | 1.15237800 | -0.15895400 | 2.32114900 |
| H | 1.09465800 | 2.11768600 | 1.41223600 |
| C | 1.23177700 | -1.23742800 | 0.46767600 |
| C | 2.52975400 | -1.78698600 | 0.05980300 |
| H | 3.38191500 | -1.11321800 | 0.04599700 |
| H | 2.76445000 | -2.82227300 | 0.28579300 |
| C | 1.41825400 | -1.52154300 | -0.95700000 |
| H | 0.91976300 | -2.39266100 | -1.37352100 |
| H | 1.55219700 | -0.69536600 | -1.64760300 |
| C | -1.41108300 | 0.10754400 | -0.05047000 |
| C | -2.28978700 | -1.13276700 | 0.23283200 |
| C | -2.70929100 | 0.89472200 | -0.35145300 |
| H | -0.84785200 | -0.04200200 | -0.97196600 |
| C | -3.40996200 | -0.46231600 | -0.59741400 |
| H | -2.55950900 | -1.17971500 | 1.29161800 |
| H | -1.90957700 | -2.10478500 | -0.08308800 |
| H | -3.09056300 | 1.37196000 | 0.55568400 |
| H | -2.68844900 | 1.62608000 | -1.15969000 |
| H | -4.43864800 | -0.57614600 | -0.25605000 |
| H | -3.34835500 | -0.75199500 | -1.64798300 |
| O | 1.02643500 | 1.54548300 | -0.58986400 |
| O | 2.44174200 | 1.39784100 | -0.77796200 |
| H | 2.67281200 | 2.26340200 | -1.13682600 |

cis-R7-R

| | | | |
|---|---|---|---|
| C | -1.67322500 | -2.34288500 | -0.14942700 |

| | | | |
|---|---|---|---|
| C | -1.49268300 | -0.85449700 | -0.26937000 |
| C | -2.87520300 | -1.44142400 | -0.23267800 |
| H | -1.43403200 | -2.95716900 | -1.00731200 |
| H | -1.44590100 | -2.79269400 | 0.80928200 |
| H | -1.16265500 | -0.48572100 | -1.23213100 |
| H | -3.45483400 | -1.28886400 | 0.66907900 |
| H | -3.45152000 | -1.43880300 | -1.14777600 |
| C | -1.00262800 | -0.09557600 | 0.92197900 |
| C | 0.43479100 | 0.37696900 | 1.02170200 |
| C | -0.67238000 | 1.36384800 | 0.81837900 |
| H | -1.47483700 | -0.38654300 | 1.85561000 |
| H | 0.86647200 | 0.37749800 | 2.01860500 |
| H | -0.97156700 | 2.06591200 | 1.58577100 |
| C | 1.42019900 | 0.06527600 | -0.07082400 |
| C | 2.81925500 | 0.72561600 | -0.02667900 |
| C | 2.12587900 | -1.31115000 | -0.03453500 |
| H | 0.97837400 | 0.22676000 | -1.05556500 |
| C | 3.40606600 | -0.60868600 | -0.54475900 |
| H | 3.12368200 | 0.91175800 | 1.00686700 |
| H | 2.97619800 | 1.62948300 | -0.61524100 |
| H | 2.24856600 | -1.65003600 | 0.99790800 |
| H | 1.69702200 | -2.11944000 | -0.62756300 |
| H | 4.36423600 | -0.93621700 | -0.14244100 |
| H | 3.45410900 | -0.62035800 | -1.63508900 |
| O | -0.72542100 | 1.94357100 | -0.47765200 |
| O | -1.93129700 | 2.33136600 | -0.78268900 |

cis-R7-TS

| | | | |
|---|---|---|---|
| C | -1.20187900 | 0.40112100 | 0.82574400 |
| C | 0.28047800 | 0.48917500 | 1.16180900 |
| C | -0.50066900 | 1.71239400 | 0.66751300 |
| H | -1.84122600 | 0.39263100 | 1.70558300 |
| H | 0.57144600 | 0.52337300 | 2.20863900 |
| H | -0.72366400 | 2.50828800 | 1.36631100 |
| C | 1.27979300 | -0.07399200 | 0.22909400 |
| C | 2.78454400 | -0.06960500 | 0.48576200 |
| C | 1.34867900 | -1.52544700 | -0.26387800 |
| H | 0.97921800 | 0.65299300 | -0.85457000 |
| C | 2.88449900 | -1.35198800 | -0.37944000 |
| H | 3.00599200 | -0.26287800 | 1.54156500 |
| H | 3.35150000 | 0.80641100 | 0.16648700 |
| H | 1.05964100 | -2.21941900 | 0.53258100 |
| H | 0.79668500 | -1.78846200 | -1.16778100 |
| H | 3.50290300 | -2.16113400 | 0.00670400 |
| H | 3.18846800 | -1.12510900 | -1.40160600 |

| | | | |
|---|---|---|---|
| O | -0.09575100 | 2.31033000 | -0.52300200 |
| O | 0.24728200 | 1.37468000 | -1.48127000 |
| C | -1.73827000 | -0.45995400 | -0.27579000 |
| C | -2.25717800 | -1.82317300 | 0.09208900 |
| C | -3.21598500 | -0.73368400 | -0.30525400 |
| H | -1.25563500 | -0.36286500 | -1.23639300 |
| H | -2.22123200 | -2.10274400 | 1.13821100 |
| H | -2.08843100 | -2.63690500 | -0.60106400 |
| H | -3.69861800 | -0.80264000 | -1.27073400 |
| H | -3.82337000 | -0.29069600 | 0.47435500 |

cis-R7-P

| | | | |
|---|---|---|---|
| C | -1.45623400 | -2.64935800 | -0.17112600 |
| C | -0.02627000 | -2.26611100 | -0.43237500 |
| C | -1.07171900 | -1.19837300 | -0.25415800 |
| H | -2.03185200 | -3.07093300 | -0.98422600 |
| H | -1.69825400 | -3.04390100 | 0.80819300 |
| H | 0.37622500 | -2.42975100 | -1.42427700 |
| H | 0.68271900 | -2.39292300 | 0.37721100 |
| H | -1.37721300 | -0.67370900 | -1.15178600 |
| C | -1.07345900 | -0.37410100 | 0.99790300 |
| C | 0.14379200 | 0.51946600 | 1.33080000 |
| C | -1.17139500 | 1.11816100 | 0.91429400 |
| H | -1.56536900 | -0.81603300 | 1.85818700 |
| H | 0.36019100 | 0.60477500 | 2.39130600 |
| H | -1.76890300 | 1.65612200 | 1.64397700 |
| C | 1.31715600 | 0.54439400 | 0.46733500 |
| C | 1.53970500 | 0.76535800 | -1.00194600 |
| C | 2.71200200 | 0.04157300 | 0.71709100 |
| C | 2.96332600 | 0.16471000 | -0.81512000 |
| H | 1.52183800 | 1.81710200 | -1.30859500 |
| H | 0.87499400 | 0.21617800 | -1.67561500 |
| H | 3.34039400 | 0.69520600 | 1.33400400 |
| H | 2.78878800 | -0.97364800 | 1.12462400 |
| H | 3.78801900 | 0.81788800 | -1.09884500 |
| H | 3.09027700 | -0.80016900 | -1.30586200 |
| O | -1.15233800 | 1.70085700 | -0.34786200 |
| O | -2.46542300 | 1.54153200 | -0.90507100 |
| H | -2.70119800 | 2.46037700 | -1.08102300 |

cis-R8-R

| | | | |
|---|---|---|---|
| C | 1.26514000 | 0.63983500 | 0.22736600 |
| C | -0.23186200 | 0.70865600 | 0.24550200 |
| C | 0.54653900 | 1.36444400 | 1.34060300 |
| H | 1.71504000 | 1.29807300 | -0.51056400 |

| | | | |
|---|---|---|---|
| H | 0.58586500 | 2.44584300 | 1.32596000 |
| H | 0.50341300 | 0.89444900 | 2.31624400 |
| C | 1.97046600 | -0.65843200 | 0.45380800 |
| C | 2.31093100 | -1.51907700 | -0.73230600 |
| C | 3.40104100 | -0.79885000 | 0.01260400 |
| H | 1.70419600 | -1.17959200 | 1.36675500 |
| H | 2.03173900 | -1.13966300 | -1.70768700 |
| H | 2.24217900 | -2.59344500 | -0.62378400 |
| H | 4.06983000 | -1.38438300 | 0.62901700 |
| H | 3.85711300 | 0.05841400 | -0.46627900 |
| C | -1.09333300 | -0.49810100 | 0.44981500 |
| C | -2.60663000 | -0.32317400 | 0.73146500 |
| C | -1.46635200 | -1.36970800 | -0.77394800 |
| H | -0.63967100 | -1.11968400 | 1.22486900 |
| C | -2.85319100 | -1.58896100 | -0.12409200 |
| H | -2.99094700 | 0.56874200 | 0.23428100 |
| H | -2.93249100 | -0.32856400 | 1.77156000 |
| H | -1.54521900 | -0.75502300 | -1.67269000 |
| H | -0.84270600 | -2.23942100 | -0.98127900 |
| H | -3.72411900 | -1.59550400 | -0.77819800 |
| H | -2.86294500 | -2.48883300 | 0.49382300 |
| O | -0.85876600 | 1.56484600 | -0.72609100 |
| O | -0.21970500 | 2.66250500 | -0.97477600 |

cis-R8-TS

| | | | |
|---|---|---|---|
| C | -1.20780100 | -0.79706400 | -0.54641200 |
| C | 0.18561200 | -0.64794700 | -0.03465500 |
| C | -0.77920400 | -1.49688000 | 0.72834800 |
| H | -1.28375000 | -1.45624000 | -1.40558700 |
| H | -0.62312000 | -2.56603200 | 0.67558500 |
| H | -1.13348900 | -1.12323700 | 1.68088400 |
| C | 0.73202900 | 0.66057000 | 0.50529700 |
| C | 2.21178700 | 0.55194900 | 0.88657300 |
| C | 1.13056100 | 1.68336600 | -0.59582800 |
| H | 0.07226600 | 1.03906600 | 1.28724100 |
| C | 2.63104400 | 1.47259800 | -0.25774500 |
| H | 2.47665500 | -0.70904200 | 0.55992700 |
| H | 2.56108800 | 0.67737300 | 1.90940000 |
| H | 0.83764100 | 1.38916600 | -1.60441800 |
| H | 0.77309000 | 2.69246000 | -0.39098900 |
| H | 3.19206200 | 0.96881300 | -1.04776500 |
| H | 3.18096400 | 2.36481900 | 0.04609400 |
| O | 1.15396900 | -1.31095000 | -0.80324900 |
| O | 2.15863000 | -1.75280000 | 0.05496500 |
| C | -2.15666800 | 0.36223000 | -0.56790400 |

| | | | |
|---|---|---|---|
| C | -3.58933600 | 0.15655000 | -0.16004900 |
| C | -2.68111100 | 1.00326400 | 0.68648100 |
| H | -2.00035900 | 1.04100200 | -1.40006200 |
| H | -3.84604800 | -0.82304100 | 0.22378500 |
| H | -4.36651300 | 0.64519100 | -0.73203100 |
| H | -2.82556900 | 2.07562100 | 0.67494500 |
| H | -2.37719600 | 0.60226100 | 1.64462900 |

cis-R8-P

| | | | |
|---|---|---|---|
| C | 3.44289400 | -0.54352100 | -0.05975200 |
| C | 2.41432600 | -1.37502200 | -0.77816200 |
| C | 2.01587800 | -0.54671900 | 0.41270000 |
| H | 4.18298300 | -1.05618400 | 0.54018100 |
| H | 3.79847900 | 0.35280600 | -0.55243200 |
| H | 2.45791300 | -2.45046000 | -0.66677800 |
| H | 2.07746700 | -1.02995500 | -1.74796400 |
| H | 1.82694100 | -1.09071600 | 1.33146900 |
| C | 1.17241800 | 0.67042000 | 0.19834900 |
| C | -0.31426100 | 0.61975900 | 0.32599300 |
| C | 0.46981400 | 1.36995100 | 1.35472800 |
| H | 1.53229600 | 1.34575100 | -0.57079900 |
| H | 0.45314500 | 2.45137200 | 1.32868100 |
| H | 0.54802900 | 0.92747400 | 2.34136700 |
| C | -1.01100800 | -0.68128500 | 0.64902200 |
| C | -2.49797000 | -0.61653200 | 0.87495800 |
| C | -1.29982600 | -1.59810500 | -0.58034300 |
| H | -0.44762900 | -1.17139700 | 1.44990900 |
| C | -2.82964600 | -1.42310300 | -0.34593000 |
| H | -3.09958400 | -0.01056900 | 1.53938100 |
| H | -0.93326800 | -1.19202600 | -1.52245100 |
| H | -0.93865400 | -2.61885500 | -0.45829200 |
| H | -3.33621100 | -0.85960100 | -1.13726000 |
| H | -3.39111600 | -2.34737800 | -0.17652600 |
| O | -1.13922200 | 1.30759600 | -0.60286800 |
| O | -0.48940400 | 2.48346700 | -1.05881100 |
| H | -1.07749300 | 3.16618400 | -0.71443900 |

cis-R9-R

| | | | |
|---|---|---|---|
| C | 1.26514000 | 0.63983500 | 0.22736600 |
| C | -0.23186200 | 0.70865600 | 0.24550200 |
| C | 0.54653900 | 1.36444400 | 1.34060300 |
| H | 1.71504000 | 1.29807300 | -0.51056400 |
| H | 0.58586500 | 2.44584300 | 1.32596000 |
| H | 0.50341300 | 0.89444900 | 2.31624400 |
| C | 1.97046600 | -0.65843200 | 0.45380800 |

| | | | |
|---|---|---|---|
| C | 2.31093100 | -1.51907700 | -0.73230600 |
| C | 3.40104100 | -0.79885000 | 0.01260400 |
| H | 1.70419600 | -1.17959200 | 1.36675500 |
| H | 2.03173900 | -1.13966300 | -1.70768700 |
| H | 2.24217900 | -2.59344500 | -0.62378400 |
| H | 4.06983000 | -1.38438300 | 0.62901700 |
| H | 3.85711300 | 0.05841400 | -0.46627900 |
| C | -1.09333300 | -0.49810100 | 0.44981500 |
| C | -2.60663000 | -0.32317400 | 0.73146500 |
| C | -1.46635200 | -1.36970800 | -0.77394800 |
| H | -0.63967100 | -1.11968400 | 1.22486900 |
| C | -2.85319100 | -1.58896100 | -0.12409200 |
| H | -2.99094700 | 0.56874200 | 0.23428100 |
| H | -2.93249100 | -0.32856400 | 1.77156000 |
| H | -1.54521900 | -0.75502300 | -1.67269000 |
| H | -0.84270600 | -2.23942100 | -0.98127900 |
| H | -3.72411900 | -1.59550400 | -0.77819800 |
| H | -2.86294500 | -2.48883300 | 0.49382300 |
| O | -0.85876600 | 1.56484600 | -0.72609100 |
| O | -0.21970500 | 2.66250500 | -0.97477600 |

cis-R9-TS

| | | | |
|---|---|---|---|
| C | 1.30928300 | 0.84791100 | 0.04396700 |
| C | -0.18234000 | 0.79550500 | 0.14894400 |
| C | 0.56958200 | 1.77636600 | 0.98413700 |
| H | 1.65307700 | 1.30101300 | -0.88074500 |
| H | 0.48625400 | 2.81974600 | 0.71027800 |
| H | 0.63165300 | 1.57196900 | 2.04690800 |
| C | -0.92315100 | -0.40183500 | 0.70763400 |
| C | -2.35719600 | -0.10697500 | 1.23131500 |
| C | -1.53970000 | -1.27094900 | -0.38978600 |
| H | -0.27453200 | -0.94420200 | 1.39937000 |
| C | -2.95203500 | -1.04069300 | 0.14203900 |
| H | -2.64221500 | 0.93972500 | 1.12506900 |
| H | -2.53766500 | -0.43184400 | 2.25549800 |
| H | -1.32176600 | -0.50269100 | -1.45602800 |
| H | -1.14469600 | -2.24762700 | -0.66462400 |
| H | -3.64231400 | -0.56112700 | -0.55433400 |
| H | -3.42614300 | -1.94550900 | 0.52883600 |
| O | -0.90497300 | 1.37266800 | -0.90619000 |
| O | -0.87712500 | 0.48345400 | -1.97948100 |
| C | 2.15669700 | -0.27131100 | 0.56201700 |
| C | 2.38972400 | -1.46238200 | -0.32870800 |
| C | 3.51329200 | -0.50451200 | -0.04051800 |
| H | 2.06967300 | -0.47123200 | 1.62471400 |

| | | | |
|---|---|---|---|
| H | 1.93699500 | -1.42389300 | -1.31245100 |
| H | 2.42991600 | -2.44531100 | 0.12232600 |
| H | 4.31121900 | -0.83719200 | 0.60976600 |
| H | 3.82239300 | 0.16244600 | -0.83545000 |

cis-R9-P

| | | | |
|---|---|---|---|
| C | 2.56689800 | -1.30929600 | -0.84303700 |
| C | 2.05533300 | -0.59889200 | 0.37967300 |
| C | 3.51328300 | -0.52074000 | 0.02265500 |
| H | 2.63405700 | -2.38898200 | -0.82215700 |
| H | 2.29620100 | -0.88883700 | -1.80391800 |
| H | 1.81446600 | -1.22647000 | 1.23017300 |
| H | 3.87902200 | 0.42371200 | -0.36071600 |
| H | 4.22111000 | -1.06754400 | 0.63124200 |
| C | 1.19654000 | 0.61418800 | 0.21106400 |
| C | -0.29474400 | 0.54726400 | 0.28986700 |
| C | 0.45100400 | 1.24079000 | 1.38208600 |
| H | 1.58174300 | 1.34071100 | -0.49785600 |
| H | 0.41175900 | 2.32087600 | 1.41411700 |
| H | 0.50246100 | 0.73875400 | 2.34167600 |
| C | -1.01197000 | -0.75930900 | 0.54016000 |
| C | -2.53024400 | -0.64129100 | 0.88696600 |
| C | -1.41402700 | -1.53231600 | -0.68671300 |
| H | -0.42902400 | -1.32276600 | 1.27567100 |
| C | -2.88430300 | -1.33127300 | -0.46385200 |
| H | -2.88877400 | 0.38129500 | 0.99185400 |
| H | -2.81430500 | -1.22217400 | 1.76374700 |
| H | -0.83792500 | -1.88266800 | -1.53337300 |
| H | -3.35852400 | -0.65709700 | -1.18600400 |
| H | -3.49606600 | -2.23576500 | -0.39150800 |
| O | -1.09948300 | 1.29057100 | -0.61409200 |
| O | -0.53541500 | 2.57284100 | -0.83483300 |
| H | -0.32363800 | 2.52492200 | -1.77476900 |

cis-R10-R

| | | | |
|---|---|---|---|
| C | 1.48090400 | 0.91318200 | -0.54196600 |
| C | 0.00884200 | 1.18595200 | -0.72632900 |
| C | 0.85447900 | 2.11906200 | 0.10289100 |
| H | 2.09518900 | 1.10289100 | -1.41760700 |
| H | -0.27636700 | 1.55183300 | -1.70651500 |
| H | 1.06762400 | 3.09883500 | -0.30334900 |
| H | 0.72396100 | 2.08375200 | 1.17640500 |
| C | 1.95409300 | -0.23861400 | 0.28793500 |
| C | 2.38926000 | -1.50317300 | -0.40082200 |
| C | 3.40961400 | -0.61077700 | 0.25380400 |

| | | | |
|---|---|---|---|
| H | 1.45413700 | -0.35847200 | 1.24162200 |
| H | 2.35454300 | -1.51287300 | -1.48377800 |
| H | 2.16073800 | -2.45044700 | 0.06990500 |
| H | 3.87199000 | -0.95169600 | 1.17040500 |
| H | 4.05593700 | -0.02901400 | -0.39181200 |
| C | -1.04206100 | 0.27759700 | -0.14303000 |
| C | -2.52083800 | 0.50728300 | -0.53874500 |
| C | -1.13519400 | -1.16571700 | -0.68883000 |
| C | -2.66492100 | -1.00160800 | -0.85761900 |
| H | -2.60699600 | 1.15442500 | -1.41229400 |
| H | -3.14414700 | 0.89675200 | 0.26587500 |
| H | -0.59096200 | -1.25622600 | -1.63018500 |
| H | -0.79357600 | -1.93603800 | 0.00250100 |
| H | -3.06516900 | -1.24412600 | -1.84122800 |
| H | -3.22655600 | -1.53465000 | -0.09285900 |
| O | -0.87776000 | 0.30627600 | 1.30522600 |
| O | -1.68291700 | -0.49553500 | 1.92842200 |

cis-R10-TS

| | | | |
|---|---|---|---|
| C | -1.37104400 | 0.62378800 | 0.83085200 |
| C | 0.12347100 | 0.60913800 | 1.13529200 |
| C | -0.54671700 | 1.84984000 | 0.69313900 |
| H | -2.00672900 | 0.61057900 | 1.71555800 |
| H | 0.43805700 | 0.53132100 | 2.17239900 |
| H | -0.63631900 | 2.78174200 | 1.23697500 |
| H | 0.02908900 | 2.00120900 | -0.57676200 |
| C | 1.10315100 | -0.04191000 | 0.17107600 |
| C | 2.61443300 | 0.15967200 | 0.41486500 |
| C | 1.32278800 | -1.56303200 | 0.18794700 |
| C | 2.83424400 | -1.30727300 | -0.03379400 |
| H | 2.82989200 | 0.30315900 | 1.47526300 |
| H | 3.07127900 | 0.95861700 | -0.16792900 |
| H | 1.10926200 | -1.96784000 | 1.17868900 |
| H | 0.76911600 | -2.12378600 | -0.56542800 |
| H | 3.52995100 | -1.90650600 | 0.55161600 |
| H | 3.10053100 | -1.36917700 | -1.08812900 |
| O | 0.75383400 | 0.28196000 | -1.17009100 |
| O | 0.78955300 | 1.65863800 | -1.34954300 |
| C | -1.97861500 | -0.11464400 | -0.32775900 |
| C | -2.40530200 | -1.54058400 | -0.11806600 |
| C | -3.44071800 | -0.45759000 | -0.26233900 |
| H | -1.58516400 | 0.13081900 | -1.30377400 |
| H | -2.25563700 | -1.96056200 | 0.86955000 |
| H | -2.27542100 | -2.23857800 | -0.93456700 |
| H | -4.01274700 | -0.41686000 | -1.17954400 |

| | | | |
|---|---|---|---|
| H | -3.98640900 | -0.16334400 | 0.62586400 |

cis-R10-P

| | | | |
|---|---|---|---|
| C | -2.52264800 | -1.45538200 | 0.26133700 |
| C | -1.99453500 | -0.16953900 | -0.31558400 |
| C | -3.46386900 | -0.47750000 | -0.38829400 |
| H | -2.30104200 | -2.37793600 | -0.25908100 |
| H | -2.55203200 | -1.53739900 | 1.34138600 |
| H | -1.43256500 | -0.24708100 | -1.23855600 |
| H | -4.12282400 | 0.08979600 | 0.25748800 |
| H | -3.88413000 | -0.73593800 | -1.35104700 |
| C | -1.52344600 | 0.90120300 | 0.61914200 |
| C | -0.02594000 | 1.15200500 | 0.85825100 |
| C | -0.86173200 | 2.11366700 | 0.12310100 |
| H | -2.12822400 | 0.99733600 | 1.52053700 |
| H | 0.21833600 | 1.39377100 | 1.89005700 |
| H | -0.75671200 | 2.45739400 | -0.89452900 |
| C | 1.03253200 | 0.29309200 | 0.19800100 |
| C | 2.48287500 | 0.40197900 | 0.73280500 |
| C | 1.02748000 | -1.21334600 | 0.56604600 |
| C | 2.54692900 | -1.14145400 | 0.85452400 |
| H | 2.52561400 | 0.91779400 | 1.69324100 |
| H | 3.19195200 | 0.87262600 | 0.05060100 |
| H | 0.41885000 | -1.39745400 | 1.45314900 |
| H | 0.70669300 | -1.87947000 | -0.23437800 |
| H | 2.87004700 | -1.52977600 | 1.81988800 |
| H | 3.13991200 | -1.59815800 | 0.06379600 |
| O | 0.91086000 | 0.55432800 | -1.19920600 |
| O | 1.78205800 | -0.32412800 | -1.90549900 |
| H | 2.37691300 | 0.30454900 | -2.33088500 |

cis-R11-R

| | | | |
|---|---|---|---|
| C | 1.48090400 | 0.91318200 | -0.54196600 |
| C | 0.00884200 | 1.18595200 | -0.72632900 |
| C | 0.85447900 | 2.11906200 | 0.10289100 |
| H | 2.09518900 | 1.10289100 | -1.41760700 |
| H | -0.27636700 | 1.55183300 | -1.70651500 |
| H | 1.06762400 | 3.09883500 | -0.30334900 |
| H | 0.72396100 | 2.08375200 | 1.17640500 |
| C | 1.95409300 | -0.23861400 | 0.28793500 |
| C | 2.38926000 | -1.50317300 | -0.40082200 |
| C | 3.40961400 | -0.61077700 | 0.25380400 |
| H | 1.45413700 | -0.35847200 | 1.24162200 |
| H | 2.35454300 | -1.51287300 | -1.48377800 |
| H | 2.16073800 | -2.45044700 | 0.06990500 |

| | | | |
|---|---|---|---|
| H | 3.87199000 | -0.95169600 | 1.17040500 |
| H | 4.05593700 | -0.02901400 | -0.39181200 |
| C | -1.04206100 | 0.27759700 | -0.14303000 |
| C | -2.52083800 | 0.50728300 | -0.53874500 |
| C | -1.13519400 | -1.16571700 | -0.68883000 |
| C | -2.66492100 | -1.00160800 | -0.85761900 |
| H | -2.60699600 | 1.15442500 | -1.41229400 |
| H | -3.14414700 | 0.89675200 | 0.26587500 |
| H | -0.59096200 | -1.25622600 | -1.63018500 |
| H | -0.79357600 | -1.93603800 | 0.00250100 |
| H | -3.06516900 | -1.24412600 | -1.84122800 |
| H | -3.22655600 | -1.53465000 | -0.09285900 |
| O | -0.87776000 | 0.30627600 | 1.30522600 |
| O | -1.68291700 | -0.49553500 | 1.92842200 |

cis-R11-TS

| | | | |
|---|---|---|---|
| C | -0.93904100 | 0.34372000 | -0.22760400 |
| C | -1.05015900 | -0.14064400 | 1.24207000 |
| C | -2.55228200 | -0.20513400 | 0.96883900 |
| C | -2.39777000 | 0.86188300 | -0.09637400 |
| H | -0.79731800 | 0.65738300 | 1.94544300 |
| H | -0.52585400 | -1.05963700 | 1.49990700 |
| H | -3.35785700 | -0.32065700 | 1.68957500 |
| H | -2.48044900 | -1.26790000 | 0.18527600 |
| H | -2.38672500 | 1.86787700 | 0.33700600 |
| H | -3.02713600 | 0.83368600 | -0.98693500 |
| O | -0.97497200 | -0.78028500 | -1.13147200 |
| O | -1.73262500 | -1.83164700 | -0.60374200 |
| C | 0.13205200 | 1.24108700 | -0.75832600 |
| C | 0.85901700 | 2.21870900 | 0.12271000 |
| C | 1.58349200 | 1.01620400 | -0.41699800 |
| H | -0.07318100 | 1.57176400 | -1.77146300 |
| H | 0.63266800 | 2.21154300 | 1.18260300 |
| H | 1.08355800 | 3.19597500 | -0.28320200 |
| H | 2.27402400 | 1.20428200 | -1.23242500 |
| C | 1.99679800 | -0.09289100 | 0.50671900 |
| C | 1.98688100 | -1.51023900 | 0.00367200 |
| C | 3.29381800 | -0.81255100 | 0.25479400 |
| H | 1.73338300 | 0.04786800 | 1.54960900 |
| H | 1.66844800 | -1.65430700 | -1.02062600 |
| H | 1.68503400 | -2.29472800 | 0.68595200 |
| H | 3.88239300 | -1.11799800 | 1.10965500 |
| H | 3.87294600 | -0.50056200 | -0.60568000 |

cis-R11-P

| | | | |
|---|---|---|---|
| C | 2.55737200 | 1.33518400 | 0.58400200 |

| | | | |
|---|---|---|---|
| C | 1.94359700 | 0.24102600 | -0.24580100 |
| C | 3.43238400 | 0.44609100 | -0.25942000 |
| H | 2.41395600 | 2.36060000 | 0.26927500 |
| H | 2.58356600 | 1.18819600 | 1.65738900 |
| H | 1.40390400 | 0.55415400 | -1.13098900 |
| H | 4.04010300 | -0.29025200 | 0.25216200 |
| H | 3.88352600 | 0.86905800 | -1.14711800 |
| C | 1.38665800 | -0.96327800 | 0.44442100 |
| C | -0.09829500 | -1.18409900 | 0.57137100 |
| C | 0.72075100 | -2.06549300 | -0.33593000 |
| H | 1.96850800 | -1.27786400 | 1.30662800 |
| H | -0.42294900 | -1.62747800 | 1.50676500 |
| H | 0.88482600 | -3.09207300 | -0.03576800 |
| H | 0.61471900 | -1.90596900 | -1.40090000 |
| C | -1.11001000 | -0.18514700 | 0.05012200 |
| C | -2.56179700 | -0.48521200 | 0.52191100 |
| C | -1.12554500 | 1.13863400 | 0.86950200 |
| C | -2.36977300 | 0.61431100 | 1.52534200 |
| H | -2.75576200 | -1.50009900 | 0.88110400 |
| H | -3.30012400 | -0.23795200 | -0.25071900 |
| H | -0.24335300 | 1.31799500 | 1.49000800 |
| H | -1.29947100 | 2.01365200 | 0.23283100 |
| H | -3.01536300 | 1.01794700 | 2.29150500 |
| O | -0.90113800 | -0.08546500 | -1.35044400 |
| O | -1.73838900 | 0.93847700 | -1.87377000 |
| H | -2.29192700 | 0.43388800 | -2.48158600 |

cis-R12-R

| | | | |
|---|---|---|---|
| C | 1.64168000 | 0.70082600 | -0.65602500 |
| C | 0.22557600 | 1.21385900 | -0.71187000 |
| C | 1.37055800 | 2.17222900 | -0.50617300 |
| H | 2.05348300 | 0.38516800 | -1.60955400 |
| H | -0.23234200 | 1.21396900 | -1.69473100 |
| H | 1.64026700 | 2.83787000 | -1.31526100 |
| H | 1.48851400 | 2.59338300 | 0.48621300 |
| C | 2.11276400 | -0.11706000 | 0.50716100 |
| C | 1.99292500 | -1.61620500 | 0.43677700 |
| C | 3.34577600 | -0.96236700 | 0.35517000 |
| H | 1.96699900 | 0.33128000 | 1.48408400 |
| H | 1.57390200 | -2.03021100 | -0.47274600 |
| H | 1.74744300 | -2.15950100 | 1.34032700 |
| H | 4.00947200 | -1.06036300 | 1.20386200 |
| H | 3.84008100 | -0.94622600 | -0.60815800 |
| C | -0.72282700 | 0.90555400 | 0.41161600 |

| | | | |
|---|---|---|---|
| C | -2.16565900 | 1.47553700 | 0.38186100 |
| C | -1.33631300 | -0.50686800 | 0.49175900 |
| H | -0.26126300 | 1.11962600 | 1.37917500 |
| C | -2.66501300 | 0.11897800 | 0.93924400 |
| H | -2.49884700 | 1.63225200 | -0.64555200 |
| H | -2.36652000 | 2.37180500 | 0.96705300 |
| H | -0.85845600 | -1.28110000 | 1.09134200 |
| H | -3.56332500 | -0.32592800 | 0.51281300 |
| H | -2.74110700 | 0.12237200 | 2.02726500 |
| O | -1.44494800 | -1.01853700 | -0.85766600 |
| O | -2.12944200 | -2.12037500 | -0.89774000 |

cis-R12-TS

| | | | |
|---|---|---|---|
| C | -1.42319400 | -0.16718000 | 0.80321500 |
| C | 0.03323000 | 0.04385100 | 0.61393800 |
| C | -0.65445000 | 0.81361300 | 1.68108800 |
| H | -1.73063800 | -1.08683100 | 1.29526700 |
| H | 0.85150700 | -0.96024300 | 1.03172700 |
| H | -0.50086200 | 0.53967900 | 2.71849400 |
| H | -0.81475600 | 1.87475300 | 1.51011300 |
| C | 0.72448700 | 0.55714800 | -0.60559300 |
| C | 1.67086500 | 1.76598800 | -0.38169600 |
| C | 2.02262600 | -0.28529100 | -0.90002100 |
| H | 0.03348100 | 0.60643500 | -1.45056600 |
| C | 2.86295800 | 0.79385300 | -0.20325800 |
| H | 1.43121700 | 2.41022100 | 0.46380600 |
| H | 1.76347100 | 2.37176200 | -1.28403900 |
| H | 2.22079100 | -0.37675100 | -1.96817000 |
| H | 3.01114300 | 0.53345900 | 0.84421700 |
| H | 3.81641200 | 1.05500000 | -0.66005000 |
| O | 2.00840000 | -1.60558900 | -0.42717500 |
| O | 1.81856500 | -1.60407300 | 0.94816300 |
| C | -2.38403100 | 0.33766100 | -0.22325300 |
| C | -3.00183300 | -0.63369400 | -1.19191700 |
| C | -3.84112800 | -0.01315100 | -0.10829500 |
| H | -2.15613700 | 1.32606000 | -0.61014000 |
| H | -2.72869100 | -1.67599500 | -1.08319600 |
| H | -3.16505500 | -0.30950500 | -2.21114800 |
| H | -4.57043800 | 0.73314900 | -0.39352700 |
| H | -4.13434400 | -0.64067700 | 0.72405500 |

cis-R12-P

| | | | |
|---|---|---|---|
| C | -1.20057500 | 1.64681600 | 0.65297600 |
| C | -2.15910200 | 0.51115600 | 0.40680900 |
| C | -2.59840200 | 1.93473300 | 0.18946700 |
| H | -0.94671200 | 1.88470700 | 1.67881000 |

| | | | |
|---|---|---|---|
| H | -0.38982800 | 1.76178900 | -0.05649000 |
| H | -2.55949000 | 0.02689800 | 1.29172000 |
| H | -2.73009600 | 2.25553100 | -0.83673000 |
| H | -3.29712500 | 2.36721500 | 0.89321900 |
| C | -1.97104200 | -0.38376500 | -0.78447300 |
| C | -0.64645500 | -1.13756500 | -1.00469400 |
| C | -1.91243700 | -1.86856100 | -0.62290700 |
| H | -2.42717100 | -0.03220000 | -1.70401100 |
| H | -0.33669700 | -1.20078200 | -2.04248800 |
| H | -2.38449600 | -2.49898900 | -1.36488400 |
| H | -1.95129400 | -2.24762100 | 0.39272800 |
| C | 0.43627000 | -1.01782300 | -0.04527600 |
| C | 0.54174000 | -1.20686800 | 1.44212600 |
| C | 1.83254000 | -0.50300900 | -0.18017800 |
| C | 1.95007000 | -0.54441900 | 1.36259000 |
| H | 0.55620500 | -2.25422400 | 1.76483400 |
| H | -0.19741600 | -0.67386600 | 2.04937300 |
| H | 2.54489700 | -1.13636000 | -0.72605800 |
| H | 2.78008600 | -1.12706600 | 1.75973600 |
| H | 1.97653700 | 0.46450900 | 1.77379000 |
| O | 1.84613800 | 0.79464000 | -0.76466900 |
| O | 3.19159100 | 1.26965500 | -0.70219900 |
| H | 3.42512400 | 1.31192900 | -1.63724100 |

cis-R13-R

| | | | |
|---|---|---|---|
| C | 1.64168000 | 0.70082600 | -0.65602500 |
| C | 0.22557600 | 1.21385900 | -0.71187000 |
| C | 1.37055800 | 2.17222900 | -0.50617300 |
| H | 2.05348300 | 0.38516800 | -1.60955400 |
| H | -0.23234200 | 1.21396900 | -1.69473100 |
| H | 1.64026700 | 2.83787000 | -1.31526100 |
| H | 1.48851400 | 2.59338300 | 0.48621300 |
| C | 2.11276400 | -0.11706000 | 0.50716100 |
| C | 1.99292500 | -1.61620500 | 0.43677700 |
| C | 3.34577600 | -0.96236700 | 0.35517000 |
| H | 1.96699900 | 0.33128000 | 1.48408400 |
| H | 1.57390200 | -2.03021100 | -0.47274600 |
| H | 1.74744300 | -2.15950100 | 1.34032700 |
| H | 4.00947200 | -1.06036300 | 1.20386200 |
| H | 3.84008100 | -0.94622600 | -0.60815800 |
| C | -0.72282700 | 0.90555400 | 0.41161600 |
| C | -2.16565900 | 1.47553700 | 0.38186100 |
| C | -1.33631300 | -0.50686800 | 0.49175900 |
| H | -0.26126300 | 1.11962600 | 1.37917500 |

| | | | |
|---|---|---|---|
| C | -2.66501300 | 0.11897800 | 0.93924400 |
| H | -2.49884700 | 1.63225200 | -0.64555200 |
| H | -2.36652000 | 2.37180500 | 0.96705300 |
| H | -0.85845600 | -1.28110000 | 1.09134200 |
| H | -3.56332500 | -0.32592800 | 0.51281300 |
| H | -2.74110700 | 0.12237200 | 2.02726500 |
| O | -1.44494800 | -1.01853700 | -0.85766600 |
| O | -2.12944200 | -2.12037500 | -0.89774000 |

cis-R13-TS

| | | | |
|---|---|---|---|
| C | -1.25115200 | -0.99643400 | 0.27404300 |
| C | -2.45870200 | -0.68495800 | 1.18282900 |
| C | -2.15331500 | 0.77720300 | 0.85394200 |
| C | -0.69393600 | 0.41581200 | 0.62172900 |
| H | -0.63328100 | -1.88088700 | 0.41556700 |
| H | -2.24176200 | -0.93051700 | 2.22574900 |
| H | -3.42288300 | -1.09629100 | 0.88654900 |
| H | -2.55416200 | 1.67483100 | 1.32003500 |
| H | -2.55400100 | 0.69930500 | -0.39954700 |
| H | -0.17789200 | 0.33905600 | 1.58686400 |
| O | -1.61425900 | -1.01306400 | -1.10486100 |
| O | -2.63099600 | -0.07720000 | -1.35447900 |
| C | 0.13103700 | 1.14737700 | -0.39319100 |
| C | 1.12978000 | 2.19203700 | 0.03562500 |
| C | 1.60932700 | 0.86983100 | -0.49450600 |
| H | -0.38191900 | 1.31820900 | -1.33329500 |
| H | 1.24260300 | 2.36043200 | 1.10104200 |
| H | 1.24673700 | 3.07580500 | -0.57724000 |
| H | 2.00576300 | 0.87447900 | -1.50488400 |
| C | 2.26674200 | -0.12534400 | 0.41197200 |
| C | 2.32325500 | -1.56860000 | -0.01255000 |
| C | 3.57695200 | -0.73647100 | 0.00228900 |
| H | 2.14792100 | 0.05858700 | 1.47436600 |
| H | 1.88857300 | -1.80930500 | -0.97553300 |
| H | 2.21583800 | -2.33640300 | 0.74302600 |
| H | 4.31195900 | -0.93824900 | 0.77002100 |
| H | 3.98862800 | -0.42965700 | -0.95109200 |

cis-R13-P

| | | | |
|---|---|---|---|
| C | -1.72514800 | 1.54961400 | 0.65398300 |
| C | -2.18419500 | 0.12393400 | 0.48710600 |
| C | -3.14734300 | 1.25627000 | 0.26452800 |
| H | -1.54112700 | 1.91637000 | 1.65596900 |
| H | -1.05624600 | 1.93328000 | -0.10716600 |
| H | -2.32975000 | -0.44154900 | 1.40128600 |
| H | -3.44050100 | 1.46010600 | -0.75793900 |

| | | | |
|---|---|---|---|
| H | -3.92336700 | 1.42138700 | 1.00011900 |
| C | -1.69581100 | -0.66701700 | -0.69206400 |
| C | -0.29505600 | -1.22245700 | -0.71218300 |
| C | -1.47194500 | -2.15082600 | -0.57500900 |
| H | -2.06139700 | -0.31456000 | -1.65085600 |
| H | 0.20606700 | -1.21532500 | -1.67313500 |
| H | -1.72957900 | -2.78619600 | -1.41198600 |
| H | -1.64229300 | -2.59416300 | 0.40008500 |
| C | 0.61411800 | -0.97931600 | 0.46720400 |
| C | 1.93732600 | -1.69268300 | 0.51111400 |
| C | 1.42006500 | 0.35662400 | 0.49594100 |
| H | 0.03579200 | -1.08573300 | 1.39453800 |
| C | 2.73969400 | -0.43473600 | 0.68325200 |
| H | 2.19040600 | -2.74165300 | 0.58272200 |
| H | 1.15509900 | 1.06464800 | 1.28416400 |
| H | 3.49495200 | -0.22370500 | -0.08163100 |
| H | 3.20789700 | -0.30079100 | 1.66476700 |
| O | 1.30366100 | 0.99038900 | -0.75919400 |
| O | 2.13971800 | 2.14595900 | -0.71016600 |
| H | 2.73678500 | 1.98065800 | -1.44927400 |

cis-R14-R

| | | | |
|---|---|---|---|
| C | -2.09992000 | 0.77647900 | 0.48302800 |
| C | -0.64478900 | 1.17033600 | 0.52747300 |
| C | -1.66054400 | 2.09502800 | -0.09229600 |
| H | -2.62109600 | 0.82295400 | 1.43452300 |
| H | -0.26691300 | 1.46133200 | 1.50367000 |
| H | -1.92122600 | 3.00638100 | 0.42914100 |
| H | -1.65255300 | 2.17713400 | -1.17361300 |
| C | -2.57120600 | -0.32959300 | -0.40912900 |
| C | -2.69345900 | -1.71990300 | 0.15266100 |
| C | -3.93012000 | -0.92846300 | -0.17945600 |
| H | -2.25981900 | -0.25837900 | -1.44532300 |
| H | -2.44486600 | -1.84885300 | 1.19922300 |
| H | -2.44075300 | -2.55923500 | -0.48203400 |
| H | -4.51093400 | -1.23021400 | -1.04079800 |
| H | -4.51184100 | -0.53430600 | 0.64450600 |
| C | 0.36118400 | 0.44956600 | -0.32131000 |
| C | 1.80729500 | 1.00889600 | -0.39461900 |
| C | 0.95495900 | -0.87666400 | 0.22507400 |
| H | -0.02691800 | 0.29393400 | -1.33070800 |
| C | 2.29125500 | -0.44601000 | -0.37577800 |
| H | 2.07844600 | 1.50076800 | 0.54296600 |
| H | 2.09384300 | 1.63406200 | -1.23809900 |

| | | | |
|---|---|---|---|
| H | 1.01305800 | -0.84407400 | 1.31628600 |
| H | 0.51654900 | -1.82035100 | -0.09531900 |
| H | 2.50270600 | -0.85415700 | -1.36473000 |
| O | 3.42025700 | -0.73754100 | 0.46657300 |
| O | 4.52529100 | -0.28058900 | -0.04077000 |

cis-R14-TS

| | | | |
|---|---|---|---|
| C | 0.82312600 | 0.65456400 | 0.27950400 |
| C | 2.29672600 | 1.04668200 | 0.29151600 |
| C | 1.15975700 | -0.54367700 | 1.18093500 |
| H | 0.85088600 | -0.03160100 | -0.82334900 |
| C | 2.56160800 | -0.45679700 | 0.54238500 |
| H | 2.54215900 | 1.62928300 | 1.18595300 |
| H | 2.73851700 | 1.50262200 | -0.59532600 |
| H | 1.18839900 | -0.25479800 | 2.23577400 |
| H | 0.59848700 | -1.46862900 | 1.05227500 |
| H | 3.45158500 | -0.78466400 | 1.07467000 |
| O | 2.61544500 | -1.12968800 | -0.71695100 |
| O | 1.37885700 | -1.03137700 | -1.37073200 |
| C | -0.35362300 | 1.55434800 | 0.36264800 |
| C | -1.11468400 | 1.94513200 | -0.88456100 |
| C | -1.73677500 | 1.00945400 | 0.11226400 |
| H | -0.26518400 | 2.32641200 | 1.12426900 |
| H | -0.77283600 | 1.51392200 | -1.81857300 |
| H | -1.49101600 | 2.95673400 | -0.95672000 |
| H | -2.52169700 | 1.43018600 | 0.73377600 |
| C | -1.93270800 | -0.43589200 | -0.21946300 |
| C | -2.47267200 | -1.36118800 | 0.83566100 |
| C | -3.33038700 | -0.97308400 | -0.33966200 |
| H | -1.19928800 | -0.86423500 | -0.89557100 |
| H | -2.71344100 | -0.92731000 | 1.79869300 |
| H | -2.09106000 | -2.37310200 | 0.87432400 |
| H | -3.52751700 | -1.72193000 | -1.09492000 |
| H | -4.14462900 | -0.28161900 | -0.16118200 |

cis-R14-P

| | | | |
|---|---|---|---|
| C | -2.76463100 | -2.02112400 | 0.04178200 |
| C | -1.37547700 | -1.80692000 | 0.57643600 |
| C | -1.95247800 | -0.84610000 | -0.42725200 |
| H | -2.92729500 | -2.83893500 | -0.64745200 |
| H | -3.59578700 | -1.81483300 | 0.70494200 |
| H | -0.58788500 | -2.47747900 | 0.25657500 |
| H | -1.28297500 | -1.44439600 | 1.59323900 |
| H | -1.55706900 | -0.92138100 | -1.43452600 |
| C | -2.32584800 | 0.53725900 | 0.01246100 |
| C | -1.22333700 | 1.55160300 | 0.36549600 |

| | | | |
|---|---|---|---|
| C | -2.06579300 | 1.71328800 | -0.87505800 |
| H | -3.20844400 | 0.59908900 | 0.64138000 |
| H | -1.46794000 | 2.19989900 | 1.20111500 |
| H | -2.78566500 | 2.52051900 | -0.90485600 |
| H | -1.57188200 | 1.50725400 | -1.81852200 |
| C | 0.17916400 | 1.17698100 | 0.26487500 |
| C | 1.01634800 | 0.60205400 | -0.84615400 |
| C | 1.20110800 | 0.86145300 | 1.32466800 |
| C | 2.17942500 | 0.54841300 | 0.17362300 |
| H | 1.17236700 | 1.21426500 | -1.73903200 |
| H | 0.73098400 | -0.41169300 | -1.15641800 |
| H | 1.49543100 | 1.65321800 | 2.01881300 |
| H | 0.97231300 | -0.04579900 | 1.90035100 |
| H | 2.94519600 | 1.31133000 | 0.01006100 |
| O | 2.77950400 | -0.71214200 | 0.36622900 |
| O | 3.47879900 | -1.02509900 | -0.83692100 |
| H | 4.39133300 | -1.05457800 | -0.52540600 |

cis-R15-R

| | | | |
|---|---|---|---|
| C | 1.68004300 | 0.99396500 | -0.33514200 |
| C | 0.17273700 | 0.93929500 | -0.31510300 |
| C | 0.90233100 | 2.00764400 | 0.45822400 |
| H | 2.11457800 | 1.34819400 | -1.26497900 |
| H | -0.32281100 | 1.26314900 | -1.22329600 |
| H | 0.85312200 | 3.02785300 | 0.10194800 |
| H | 0.92778600 | 1.90452700 | 1.53755100 |
| C | 2.50240200 | -0.04964600 | 0.35514800 |
| C | 2.98412500 | -1.23863400 | -0.43081500 |
| C | 3.95934900 | -0.18045500 | 0.01075600 |
| H | 2.24999000 | -0.23338300 | 1.39363900 |
| H | 2.71773200 | -1.27116500 | -1.48037900 |
| H | 3.02230400 | -2.20263500 | 0.05949300 |
| H | 4.65433500 | -0.42761800 | 0.80210600 |
| H | 4.35109600 | 0.48980600 | -0.74418700 |
| C | -0.51802400 | -0.18598800 | 0.39804600 |
| C | -2.03600000 | -0.10600200 | 0.65777100 |
| C | -0.76961800 | -1.52304200 | -0.34639400 |
| H | -0.03532400 | -0.39020100 | 1.35739400 |
| C | -2.13641500 | -1.61251500 | 0.37704300 |
| H | -2.42135800 | 0.31111600 | 1.58803200 |
| H | -0.89344700 | -1.35096300 | -1.41722000 |
| H | -0.04987600 | -2.32522100 | -0.18901000 |
| H | -3.01337600 | -1.92882200 | -0.18639500 |
| H | -2.07399200 | -2.18094600 | 1.30583300 |

| | | | |
|---|---|---|---|
| O | -2.63523200 | 0.64424400 | -0.42578800 |
| O | -3.93056100 | 0.56807900 | -0.40142800 |

cis-R15-TS

| | | | |
|---|---|---|---|
| C | 1.30723400 | -0.87493400 | 0.25298500 |
| C | -0.12318400 | -0.77782300 | -0.13021900 |
| C | 0.67511700 | -1.89375700 | -0.69165300 |
| H | 1.53573800 | -1.25539800 | 1.24586600 |
| H | -1.07183700 | -1.13919100 | 0.77877000 |
| H | 0.52928700 | -2.89657400 | -0.30775900 |
| H | 0.93857500 | -1.83989100 | -1.74432500 |
| C | -0.79709200 | 0.31214700 | -0.89326800 |
| C | -2.32350800 | 0.40471100 | -0.50909400 |
| C | -0.63986300 | 1.74401700 | -0.31424300 |
| H | -0.63003000 | 0.19506900 | -1.96740500 |
| C | -2.01807200 | 1.61872900 | 0.38132600 |
| H | -2.96360000 | 0.62100500 | -1.36435200 |
| H | 0.22201900 | 1.89438800 | 0.33485000 |
| H | -0.63451700 | 2.49858500 | -1.10187300 |
| H | -1.92188600 | 1.30918000 | 1.42105400 |
| H | -2.70412200 | 2.46149100 | 0.30900900 |
| O | -2.86831800 | -0.75970900 | 0.05553100 |
| O | -2.15605700 | -1.09500300 | 1.19970500 |
| C | 2.28956300 | 0.12569700 | -0.26442600 |
| C | 2.96215500 | 1.06876900 | 0.69431900 |
| C | 3.74813100 | -0.04593400 | 0.05928500 |
| H | 2.05487300 | 0.52553100 | -1.24629000 |
| H | 2.70789300 | 0.97124600 | 1.74286000 |
| H | 3.15645800 | 2.08138500 | 0.36579500 |
| H | 4.47473800 | 0.21105700 | -0.69982100 |
| H | 4.01852400 | -0.88991500 | 0.68165100 |

cis-R15-P

| | | | |
|---|---|---|---|
| C | -3.70007300 | -0.63722100 | -0.58381300 |
| C | -3.17479300 | 0.64225600 | -1.18266900 |
| C | -2.69864800 | 0.19126000 | 0.16864400 |
| H | -4.72396200 | -0.65281400 | -0.23441900 |
| H | -3.34926100 | -1.57025200 | -1.00743100 |
| H | -3.84249100 | 1.49186400 | -1.23708200 |
| H | -2.47525400 | 0.55994600 | -2.00571800 |
| H | -3.06840900 | 0.74984500 | 1.02228000 |
| C | -1.29416200 | -0.30956600 | 0.31610700 |
| C | -0.31484100 | 0.43829600 | 1.11108400 |
| C | -0.82539700 | -0.81568300 | 1.68419400 |
| H | -0.91154400 | -0.84702400 | -0.55070700 |
| H | -0.16456800 | -1.67736700 | 1.71392400 |

| | | | |
|---|---|---|---|
| H | -1.55337400 | -0.76927800 | 2.48978600 |
| C | 1.03730400 | 0.96532600 | 0.85145400 |
| C | 2.17819300 | -0.00618800 | 0.40939700 |
| C | 1.31331400 | 1.78608100 | -0.43104800 |
| H | 1.41117400 | 1.49914700 | 1.73150800 |
| C | 2.69596600 | 1.09803200 | -0.52714200 |
| H | 2.84444600 | -0.41296100 | 1.17508200 |
| H | 0.64706600 | 1.46881100 | -1.23440700 |
| H | 1.28926200 | 2.87137000 | -0.34309000 |
| H | 3.03788200 | 0.75586000 | -1.50273800 |
| H | 3.48157900 | 1.68254800 | -0.04591200 |
| O | 1.57133200 | -1.06641200 | -0.30764400 |
| O | 2.61205400 | -1.76531500 | -0.98569100 |
| H | 2.60919300 | -2.61143000 | -0.52163800 |

cis-R16-R

| | | | |
|---|---|---|---|
| C | 1.68004300 | 0.99396500 | -0.33514200 |
| C | 0.17273700 | 0.93929500 | -0.31510300 |
| C | 0.90233100 | 2.00764400 | 0.45822400 |
| H | 2.11457800 | 1.34819400 | -1.26497900 |
| H | -0.32281100 | 1.26314900 | -1.22329600 |
| H | 0.85312200 | 3.02785300 | 0.10194800 |
| H | 0.92778600 | 1.90452700 | 1.53755100 |
| C | 2.50240200 | -0.04964600 | 0.35514800 |
| C | 2.98412500 | -1.23863400 | -0.43081500 |
| C | 3.95934900 | -0.18045500 | 0.01075600 |
| H | 2.24999000 | -0.23338300 | 1.39363900 |
| H | 2.71773200 | -1.27116500 | -1.48037900 |
| H | 3.02230400 | -2.20263500 | 0.05949300 |
| H | 4.65433500 | -0.42761800 | 0.80210600 |
| H | 4.35109600 | 0.48980600 | -0.74418700 |
| C | -0.51802400 | -0.18598800 | 0.39804600 |
| C | -2.03600000 | -0.10600200 | 0.65777100 |
| C | -0.76961800 | -1.52304200 | -0.34639400 |
| H | -0.03532400 | -0.39020100 | 1.35739400 |
| C | -2.13641500 | -1.61251500 | 0.37704300 |
| H | -2.42135800 | 0.31111600 | 1.58803200 |
| H | -0.89344700 | -1.35096300 | -1.41722000 |
| H | -0.04987600 | -2.32522100 | -0.18901000 |
| H | -3.01337600 | -1.92882200 | -0.18639500 |
| H | -2.07399200 | -2.18094600 | 1.30583300 |
| O | -2.63523200 | 0.64424400 | -0.42578800 |
| O | -3.93056100 | 0.56807900 | -0.40142800 |

cis-R16-TS

| | | | |
|---|---|---|---|
| C | 1.01076500 | -1.29582100 | -0.01518100 |
| C | 2.28954100 | -1.32091400 | -0.85308000 |
| C | 0.65414600 | 0.05904900 | -0.60845200 |
| H | 1.59048500 | -0.85780900 | 1.08036000 |
| C | 2.19356500 | 0.21830400 | -0.79399100 |
| H | 2.11416100 | -1.68876200 | -1.86747100 |
| H | 3.18297900 | -1.77863200 | -0.43013400 |
| H | 0.21695900 | -0.08376100 | -1.60460900 |
| H | 2.59589900 | 0.84995500 | -1.58348900 |
| O | 2.75056000 | 0.73571100 | 0.41131800 |
| O | 2.52628500 | -0.15250000 | 1.47663800 |
| H | 0.33232400 | -2.11310100 | 0.22087100 |
| C | -0.11010700 | 1.06353200 | 0.20063400 |
| C | -1.02638100 | 2.05473600 | -0.46831900 |
| C | -1.60581700 | 0.92923100 | 0.34271900 |
| H | 0.42569200 | 1.41144100 | 1.07697000 |
| H | -1.13026600 | 1.98282400 | -1.54556200 |
| H | -1.07121800 | 3.06136500 | -0.07488300 |
| H | -2.00281900 | 1.19702200 | 1.31727900 |
| C | -2.34561600 | -0.18860500 | -0.32233800 |
| C | -2.71163100 | -1.40546900 | 0.48336100 |
| C | -3.78045000 | -0.43943300 | 0.04578500 |
| H | -2.09000700 | -0.36350700 | -1.36187700 |
| H | -2.43078800 | -1.40361200 | 1.52972400 |
| H | -2.67600600 | -2.37494200 | 0.00341400 |
| H | -4.46365200 | -0.75516000 | -0.73132600 |
| H | -4.21660400 | 0.20333200 | 0.80025800 |
| cis-R16-P | | | |
| C | -4.01731200 | -0.08333600 | 0.11281500 |
| C | -3.20370000 | 1.14588900 | -0.19460000 |
| C | -2.53973900 | -0.07237400 | 0.38629800 |
| H | -4.69659700 | -0.04620400 | 0.95405000 |
| H | -4.36138400 | -0.68083400 | -0.72230500 |
| H | -3.33392300 | 2.01502200 | 0.43690200 |
| H | -2.99944200 | 1.36487100 | -1.23579500 |
| H | -2.25732800 | -0.00840200 | 1.43108300 |
| C | -1.63067100 | -0.88602000 | -0.48068100 |
| C | -0.13566800 | -0.70871300 | -0.43288700 |
| C | -0.76185000 | -1.96579300 | 0.10810100 |
| H | -2.03945800 | -1.08902000 | -1.46626400 |
| H | 0.38363500 | -0.80137200 | -1.38001300 |
| H | -0.62642600 | -2.88910300 | -0.43924100 |
| H | -0.78592400 | -2.07630600 | 1.18670100 |
| C | 0.46495400 | 0.31827700 | 0.50590700 |

| | | | |
|---|---|---|---|
| C | 2.02194500 | 0.35994700 | 0.54162600 |
| C | 0.53623700 | 1.71245000 | -0.05849200 |
| H | 0.01003800 | 0.22252300 | 1.49686400 |
| C | 2.01583000 | 1.65263600 | -0.30880000 |
| H | 2.42626400 | 0.50816400 | 1.54689800 |
| H | -0.24171900 | 2.39248200 | -0.37739200 |
| H | 2.28692500 | 1.43755000 | -1.35040200 |
| H | 2.63642000 | 2.47913400 | 0.04561600 |
| O | 2.59669700 | -0.78270800 | -0.04620500 |
| O | 3.99557900 | -0.52330100 | -0.15484800 |
| H | 4.36055500 | -1.21821000 | 0.40600500 |

trans-R1-R

| | | | |
|---|---|---|---|
| C | 0.37875500 | 0.47516600 | -0.00239600 |
| C | -0.25898700 | 1.83809800 | -0.01663300 |
| H | 0.50586000 | -0.00553300 | -0.96663300 |
| H | -0.53197200 | 2.25998600 | -0.97601000 |
| H | 0.08531500 | 2.55752600 | 0.71611700 |
| C | 1.45125900 | 0.18033300 | 0.99626500 |
| C | 2.09635700 | -1.18534400 | 1.02817200 |
| C | 2.86722800 | 0.00655000 | 0.55751100 |
| H | 1.32919500 | 0.68387800 | 1.95010500 |
| H | 1.78413600 | -1.88222100 | 0.25967800 |
| H | 2.37669000 | -1.62673300 | 1.97495100 |
| H | 3.70135500 | 0.39820600 | 1.12442600 |
| O | 3.07702700 | 0.11851500 | -0.84394300 |
| O | 4.19483700 | -0.43826700 | -1.20974800 |
| C | -1.03906600 | 0.64394800 | 0.46801400 |
| H | -1.18861000 | 0.57715100 | 1.54321700 |
| C | -2.17019200 | 0.10226200 | -0.35460700 |
| C | -2.67908200 | -1.33450500 | -0.08173500 |
| C | -3.60965300 | 0.60393400 | -0.08140900 |
| H | -1.93821500 | 0.21449100 | -1.41957400 |
| C | -4.11135200 | -0.82945300 | -0.37321800 |
| H | -2.55845000 | -1.58428700 | 0.97594200 |
| H | -2.27876700 | -2.14725900 | -0.68861900 |
| H | -3.72891800 | 0.85357200 | 0.97639200 |
| H | -3.99346200 | 1.42492400 | -0.68755200 |
| H | -4.92615000 | -1.22075100 | 0.23540500 |
| H | -4.36451900 | -0.95088000 | -1.42810200 |

trans-R1-TS

| | | | |
|---|---|---|---|
| C | 0.61180100 | 0.82689500 | -0.07490600 |
| C | -0.16853500 | 2.03198000 | -0.43163600 |
| H | 0.94974400 | -0.15209500 | -0.98828300 |

| | | | |
|---|---|---|---|
| H | -0.42630200 | 2.19931500 | -1.47236100 |
| H | -0.00219600 | 2.93578600 | 0.14578300 |
| C | 1.75687800 | 0.73438500 | 0.84198000 |
| C | 2.07346400 | -0.62440600 | 1.44175500 |
| C | 2.92871900 | -0.11502800 | 0.34029500 |
| H | 2.04667900 | 1.63474000 | 1.37490600 |
| H | 1.37205200 | -1.41198300 | 1.19747600 |
| H | 2.51552100 | -0.66660000 | 2.42849000 |
| H | 3.93900300 | 0.21474500 | 0.54154700 |
| O | 2.87462000 | -0.58596400 | -0.96933200 |
| O | 1.61161500 | -1.06658900 | -1.26763400 |
| C | -0.83879200 | 0.83533800 | 0.23703600 |
| H | -1.09190100 | 0.94380700 | 1.29145700 |
| C | -1.80026300 | -0.00452500 | -0.56643200 |
| C | -2.09987500 | -1.37216400 | 0.10111300 |
| C | -3.28508400 | 0.37877900 | -0.33702800 |
| H | -1.46254500 | -0.04138200 | -1.60559500 |
| C | -3.37686000 | -0.75105600 | 0.71732100 |
| H | -1.34001700 | -1.76355400 | 0.78017000 |
| H | -2.33947200 | -2.13470300 | -0.64157500 |
| H | -3.48413600 | 1.40123900 | -0.01092900 |
| H | -3.89526000 | 0.15819400 | -1.21439400 |
| H | -3.19964200 | -0.37767600 | 1.72734800 |
| H | -4.28012800 | -1.36059000 | 0.72471200 |

trans-R1-P

| | | | |
|---|---|---|---|
| C | 2.17593700 | -0.13167900 | 1.26806900 |
| C | 2.01789100 | 1.03694000 | 0.32384600 |
| C | 2.32028200 | -0.36193300 | -0.19766400 |
| H | 3.05821000 | -0.19777900 | 1.89016000 |
| H | 1.25365500 | -0.49754000 | 1.70340500 |
| H | 2.87051000 | 1.70131900 | 0.22485600 |
| H | 3.30593200 | -0.54553900 | -0.61496700 |
| C | 0.72804100 | 1.69514700 | 0.18417300 |
| C | -0.71588100 | 1.47839600 | 0.24036600 |
| C | -0.07863800 | 2.41855500 | -0.79732700 |
| H | -1.22945600 | 1.98736100 | 1.05929800 |
| H | -0.07436700 | 2.06794700 | -1.82837800 |
| H | -0.23359900 | 3.48847800 | -0.68805400 |
| C | -1.40603100 | 0.20941100 | -0.18927300 |
| C | -1.52526600 | -0.95273200 | 0.82522800 |
| C | -2.95152100 | 0.21095000 | -0.28995600 |
| H | -0.96614300 | -0.16216100 | -1.11639100 |
| C | -2.93725000 | -1.23470200 | 0.25923700 |
| H | -1.58461900 | -0.56409300 | 1.84675800 |

| | | | |
|---|---|---|---|
| H | -0.77602500 | -1.74318500 | 0.77303700 |
| H | -3.38213300 | 0.90216600 | 0.44014800 |
| H | -3.40415000 | 0.39438100 | -1.26513400 |
| H | -3.72249700 | -1.52377100 | 0.95790600 |
| H | -2.89518000 | -1.96449300 | -0.55155200 |
| O | 1.28087000 | -0.97853700 | -0.87744200 |
| O | 1.28829700 | -2.36825800 | -0.52395200 |
| H | 1.46114100 | -2.77885700 | -1.38013500 |

trans-R2-R

| | | | |
|---|---|---|---|
| C | -0.44086500 | -1.01506900 | -0.36400300 |
| C | 0.46294900 | -2.21868300 | -0.38264200 |
| H | -0.53254900 | -0.46477200 | -1.29457900 |
| H | 0.94170000 | -2.47881900 | -1.31874300 |
| H | 0.18543700 | -3.06281700 | 0.23665600 |
| C | -1.67694500 | -1.04211500 | 0.47620900 |
| C | -2.51347600 | 0.18661900 | 0.61135200 |
| C | -3.03674900 | -0.97091500 | -0.17782300 |
| H | -1.59853100 | -1.64273700 | 1.37687100 |
| H | -2.96645500 | 0.47974500 | 1.54912900 |
| H | -3.86400500 | -1.53107900 | 0.23652000 |
| H | -3.04740100 | -0.84640200 | -1.25387900 |
| O | -2.04654600 | 1.30765900 | -0.12809200 |
| O | -2.99025500 | 2.18143200 | -0.32657000 |
| C | 0.90662600 | -0.94968500 | 0.29712500 |
| H | 0.89539800 | -0.96411700 | 1.38461900 |
| C | 1.98596700 | -0.10077400 | -0.30560000 |
| C | 2.09316300 | 1.38264700 | 0.12319700 |
| C | 3.45046400 | -0.28517000 | 0.16237400 |
| H | 1.93834200 | -0.16500200 | -1.39849500 |
| C | 3.63094500 | 1.24547300 | 0.03264200 |
| H | 1.76954300 | 1.49967700 | 1.16108500 |
| H | 1.59445000 | 2.13266500 | -0.49069200 |
| H | 3.47684400 | -0.59524700 | 1.21057700 |
| H | 4.10047900 | -0.93905400 | -0.41981300 |
| H | 4.23607700 | 1.75488100 | 0.78218500 |
| H | 3.99259900 | 1.51638200 | -0.96112600 |

trans-R2-TS

| | | | |
|---|---|---|---|
| C | -0.56833500 | 0.62521700 | 0.06587800 |
| C | 0.11296900 | 1.81338600 | 0.64810000 |
| H | -1.05153000 | -0.27593100 | 1.00047500 |
| H | 0.33265500 | 1.82825000 | 1.71047000 |
| H | -0.11294400 | 2.78192300 | 0.21435500 |
| C | -1.66227800 | 0.58540200 | -0.91679100 |

| | | | |
|---|---|---|---|
| C | -2.66259200 | -0.55541800 | -0.73403400 |
| C | -3.07462900 | 0.83764500 | -0.42104100 |
| H | -1.41100800 | 0.80840900 | -1.94959500 |
| H | -3.05500000 | -1.05758000 | -1.60795500 |
| H | -3.78585800 | 1.30226000 | -1.09117600 |
| H | -3.15862500 | 1.10473400 | 0.62449600 |
| O | -2.37883600 | -1.51851100 | 0.23317700 |
| O | -1.92432300 | -0.92482100 | 1.39786100 |
| C | 0.87653800 | 0.78347100 | -0.18020000 |
| H | 1.16073000 | 1.08510200 | -1.18843600 |
| C | 1.87543600 | -0.10706600 | 0.51913100 |
| C | 2.31068200 | -1.31424000 | -0.35121000 |
| C | 3.32961100 | 0.42306200 | 0.42837200 |
| H | 1.50470700 | -0.34508300 | 1.51928100 |
| C | 3.55272700 | -0.50248700 | -0.79259900 |
| H | 1.60942000 | -1.64234000 | -1.12041600 |
| H | 2.58770400 | -2.17054000 | 0.26562400 |
| H | 3.45305600 | 1.49823800 | 0.28581100 |
| H | 3.92543100 | 0.10559900 | 1.28569700 |
| H | 3.38233100 | 0.02115700 | -1.73484500 |
| H | 4.50342000 | -1.03137300 | -0.85572300 |

trans-R2-P

| | | | |
|---|---|---|---|
| C | 1.95107600 | -0.40852100 | 0.82090900 |
| C | 1.31268400 | 0.96821900 | 0.96387400 |
| C | 2.79024000 | 0.81474900 | 0.68726700 |
| H | 2.03819100 | -1.02057900 | 1.71389000 |
| H | 1.03296200 | 1.18766000 | 1.98907000 |
| H | 3.09734000 | 1.05387200 | -0.32341200 |
| H | 3.50236600 | 1.04054200 | 1.46922600 |
| C | 0.42940100 | 1.55181800 | -0.04064200 |
| C | -0.95503800 | 1.28940000 | -0.45083900 |
| C | 0.18938600 | 1.35236600 | -1.47272700 |
| H | -1.64293800 | 2.13460900 | -0.46880600 |
| H | 0.51069500 | 0.42252400 | -1.93209200 |
| H | 0.21123700 | 2.21982300 | -2.12567500 |
| C | -1.59868300 | -0.02982000 | -0.12034800 |
| C | -2.37014900 | -0.16553800 | 1.21563200 |
| C | -2.90363900 | -0.43553900 | -0.84938500 |
| H | -0.85899900 | -0.83167000 | -0.20599000 |
| C | -3.39187300 | -1.06161600 | 0.47785400 |
| H | -2.81818900 | 0.79198000 | 1.49561000 |
| H | -1.84002100 | -0.57753700 | 2.07562900 |
| H | -3.48912200 | 0.45269300 | -1.10239800 |
| H | -2.82384900 | -1.07426300 | -1.72938400 |

| | | | |
|---|---|---|---|
| H | -4.44555700 | -0.95767300 | 0.73671400 |
| H | -3.11108600 | -2.11459300 | 0.54279500 |
| O | 1.62079700 | -1.11412000 | -0.32921900 |
| O | 2.80348900 | -1.79405700 | -0.77259300 |
| H | 2.52225400 | -2.71507800 | -0.71026700 |

trans-R3-R

| | | | |
|---|---|---|---|
| C | 2.98104800 | 0.67467100 | 0.98788800 |
| C | 2.29511300 | 1.58324500 | -0.00765400 |
| C | 1.80372400 | 0.19215800 | 0.20987900 |
| H | 3.94561500 | 0.28466100 | 0.69026900 |
| H | 2.83483400 | 0.84323700 | 2.04688600 |
| H | 2.82762900 | 1.76497200 | -0.93207200 |
| H | 1.66815900 | 2.38786400 | 0.35426700 |
| C | 0.48741700 | -0.09132000 | 0.85187500 |
| C | -0.68803800 | -0.56798800 | 0.04640500 |
| C | -0.00749700 | -1.50315800 | 1.01307400 |
| H | 0.26359200 | 0.59798000 | 1.65990100 |
| H | -0.48862900 | -0.75955800 | -1.00436500 |
| H | -0.53175700 | -1.74942500 | 1.92830300 |
| H | 0.60380800 | -2.29499600 | 0.60050200 |
| C | -2.05756400 | -0.03591400 | 0.34786900 |
| C | -2.55865600 | 1.22072100 | -0.40549800 |
| C | -3.29482800 | -0.78895900 | -0.19985000 |
| H | -2.16763200 | 0.11358700 | 1.42816800 |
| C | -3.96784900 | 0.58431300 | -0.42924700 |
| H | -2.12692700 | 1.25680900 | -1.40922900 |
| H | -2.43336400 | 2.19304900 | 0.07239600 |
| H | -3.05311700 | -1.27152300 | -1.15044900 |
| H | -3.78913200 | -1.51031900 | 0.45131200 |
| H | -4.57287900 | 0.71436200 | -1.32608100 |
| H | -4.55211300 | 0.88746200 | 0.44164500 |
| O | 2.04108200 | -0.76873800 | -0.83161500 |
| O | 3.16075300 | -0.61435900 | -1.46087100 |

trans-R3-TS

| | | | |
|---|---|---|---|
| C | -0.50433500 | 0.40627800 | -0.38590200 |
| C | 0.07082900 | -0.88064400 | -0.83715400 |
| H | -0.23333900 | 1.31368200 | -0.91870800 |
| H | 0.65065100 | -1.05502400 | -1.73526800 |
| H | -1.01202100 | -1.72963700 | -0.65825800 |
| C | -1.93311700 | 0.34067100 | 0.09060900 |
| C | -2.63747600 | 1.47582500 | 0.74555000 |
| C | -3.02961700 | 1.03495600 | -0.64950100 |
| H | -2.08802800 | 2.40082900 | 0.86285500 |

| | | | |
|---|---|---|---|
| H | -3.31263700 | 1.22433900 | 1.55273700 |
| H | -3.95440700 | 0.48397500 | -0.75567300 |
| H | -2.75566800 | 1.66461900 | -1.48625200 |
| O | -2.22615100 | -0.90656500 | 0.65726200 |
| O | -2.10197900 | -1.88861600 | -0.32648200 |
| C | 1.94343400 | 0.21695300 | 0.59420000 |
| C | 3.13777700 | -0.76689300 | 0.54117000 |
| C | 2.60552500 | 0.99855600 | -0.56698500 |
| H | 2.00505900 | 0.81527900 | 1.50839700 |
| C | 3.94790000 | 0.33649600 | -0.17615700 |
| H | 2.91371200 | -1.59486200 | -0.13635500 |
| H | 3.51868500 | -1.16462300 | 1.48179600 |
| H | 2.26247300 | 0.63334900 | -1.53885300 |
| H | 2.54108000 | 2.08703800 | -0.55205500 |
| H | 4.62084800 | 0.03788300 | -0.97932700 |
| H | 4.49641500 | 0.95223200 | 0.53875700 |
| C | 0.54046900 | -0.31700700 | 0.44872700 |
| H | 0.12389400 | -0.77877900 | 1.34263000 |

trans-R3-P

| | | | |
|---|---|---|---|
| C | 2.07138600 | -1.58802900 | 0.23565500 |
| C | 1.75573400 | -0.20936200 | -0.23274700 |
| C | 2.81465700 | -0.98140600 | -0.94679200 |
| H | 2.63577200 | -1.68515000 | 1.15247000 |
| H | 1.33201500 | -2.35702500 | 0.04960000 |
| H | 2.59347200 | -1.33526800 | -1.94572500 |
| H | 3.84673100 | -0.71249800 | -0.76618100 |
| C | 0.44083700 | 0.06072200 | -0.89773600 |
| C | -0.73854600 | 0.64715900 | -0.12308700 |
| C | -0.03754200 | 1.42564900 | -1.15727200 |
| H | 0.16954100 | -0.71053300 | -1.61685200 |
| H | -0.53919000 | 0.92864400 | 0.91063900 |
| H | 0.48416800 | 2.36758800 | -1.09056800 |
| C | -2.10784100 | 0.07174400 | -0.36878900 |
| C | -2.56539500 | -1.14008300 | 0.47807300 |
| C | -3.34825600 | 0.83706300 | 0.15339500 |
| H | -2.23065400 | -0.14565500 | -1.43493000 |
| C | -3.98692600 | -0.53067200 | 0.48989100 |
| H | -2.11137300 | -1.10042300 | 1.47187500 |
| H | -2.43015900 | -2.13959200 | 0.06290000 |
| H | -3.09870200 | 1.38804000 | 1.06401300 |
| H | -3.86914800 | 1.50166100 | -0.53601300 |
| H | -4.56974200 | -0.61119900 | 1.40718400 |
| H | -4.58298300 | -0.90375400 | -0.34508800 |
| O | 2.08192400 | 0.91986300 | 0.55785600 |

| | | | |
|---|---|---|---|
| O | 3.25721900 | 0.66211600 | 1.30597600 |
| H | 3.86845200 | 1.30263200 | 0.92248900 |

trans-R4-R

| | | | |
|---|---|---|---|
| C | 3.36475900 | 0.43258600 | -0.14003600 |
| C | 2.54190900 | 1.64079000 | 0.22315600 |
| C | 1.99919200 | 0.61866400 | -0.73394300 |
| H | 4.21801300 | 0.57058100 | -0.79046500 |
| H | 3.48255300 | -0.33661500 | 0.61241000 |
| H | 2.83638300 | 2.60256400 | -0.17459200 |
| H | 2.11127900 | 1.66337800 | 1.21598500 |
| H | 1.95487300 | 0.89952700 | -1.78047500 |
| C | 0.85972800 | -0.26115100 | -0.33049400 |
| C | -0.51849300 | -0.06116900 | -0.88111700 |
| C | 0.28978200 | -1.29102300 | -1.24330500 |
| H | -0.58845700 | 0.71839900 | -1.63568200 |
| H | -0.01600200 | -2.22578100 | -0.78914500 |
| H | 0.72729900 | -1.37500000 | -2.22971500 |
| C | -1.72835500 | -0.19072300 | -0.00091700 |
| C | -2.24657500 | 1.06413200 | 0.74070900 |
| C | -3.12043800 | -0.30666800 | -0.66836900 |
| H | -1.59023000 | -1.00692000 | 0.71317800 |
| C | -3.68534000 | 0.55910000 | 0.48244400 |
| H | -2.03608800 | 1.96572900 | 0.15888900 |
| H | -1.92051800 | 1.21663400 | 1.76955600 |
| H | -3.13767000 | 0.24083600 | -1.61459100 |
| H | -3.53459600 | -1.30305000 | -0.82399700 |
| H | -4.45056300 | 1.29554300 | 0.23890600 |
| H | -4.03827600 | -0.06318000 | 1.30673100 |
| O | 0.88113400 | -0.58710300 | 1.06458700 |
| O | 1.54949000 | -1.67413100 | 1.32719200 |

trans-R4-TS

| | | | |
|---|---|---|---|
| C | -0.75950400 | -0.30973000 | 0.31200300 |
| C | -0.16572500 | -1.42054600 | 1.12423600 |
| H | -0.00383800 | -2.35181600 | 0.59506000 |
| H | -0.46679900 | -1.51273700 | 2.16007600 |
| C | -1.81755100 | 0.59603700 | 0.89321200 |
| C | -2.33589800 | 1.69854900 | -0.01319800 |
| C | -3.14993600 | 0.49320700 | 0.26323700 |
| H | -1.74818900 | 0.75217700 | 1.96469400 |
| H | -1.84227400 | 1.77038200 | -0.97773900 |
| H | -2.65634100 | 2.64104300 | 0.41562100 |
| H | -4.11018200 | 0.44950400 | 0.75962700 |
| H | -2.91853300 | -0.50286700 | -0.68370300 |

| | | | |
|---|---|---|---|
| O | -0.98764500 | -0.54424900 | -1.04938300 |
| O | -2.19542600 | -1.22870000 | -1.19419100 |
| C | 0.68033500 | -0.23860600 | 0.69864600 |
| H | 0.89724400 | 0.49495400 | 1.47194900 |
| C | 1.76412500 | -0.41029400 | -0.33492200 |
| C | 2.23986200 | 0.93974500 | -0.93202700 |
| C | 3.17053500 | -0.56879600 | 0.30106100 |
| H | 1.46683200 | -1.17327500 | -1.05460100 |
| C | 3.39861800 | 0.94844700 | 0.09435600 |
| H | 1.52621600 | 1.76539700 | -0.90666800 |
| H | 2.60482400 | 0.81598600 | -1.95272300 |
| H | 3.21071400 | -0.95845500 | 1.31988700 |
| H | 3.82920600 | -1.16347700 | -0.33390600 |
| H | 3.14621100 | 1.52249400 | 0.98763200 |
| H | 4.38031000 | 1.26620800 | -0.25623300 |

trans-R4-P

| | | | |
|---|---|---|---|
| C | 2.47736600 | 1.75653200 | 0.28241500 |
| C | 1.97023900 | 0.74034900 | -0.73840900 |
| C | 3.31763700 | 0.63980800 | -0.17201700 |
| H | 2.68926000 | 2.75660100 | -0.08243700 |
| H | 2.05721300 | 1.71471100 | 1.28337000 |
| H | 1.88491100 | 1.10430900 | -1.76020400 |
| H | 3.80849800 | -0.15775600 | 0.36165200 |
| C | 0.87815400 | -0.21645500 | -0.35961900 |
| C | -0.51880800 | 0.00763700 | -0.88155000 |
| C | 0.30241700 | -1.15714600 | -1.37512700 |
| H | -0.60253700 | 0.85932500 | -1.55228200 |
| H | 0.02671300 | -2.13793700 | -1.00768100 |
| H | 0.72064500 | -1.13012300 | -2.37307900 |
| C | -1.71752000 | -0.21771300 | -0.00765100 |
| C | -2.22916600 | 0.94952000 | 0.86896300 |
| C | -3.11718800 | -0.26344100 | -0.66887900 |
| H | -1.56687400 | -1.10376800 | 0.61290100 |
| C | -3.67024400 | 0.47145300 | 0.57492400 |
| H | -2.02692300 | 1.90897900 | 0.38462900 |
| H | -1.89141200 | 0.99234200 | 1.90464000 |
| H | -3.14633000 | 0.38334000 | -1.55003800 |
| H | -3.53259800 | -1.23796000 | -0.92689200 |
| H | -4.44030400 | 1.22723400 | 0.42189600 |
| H | -4.01167300 | -0.23768100 | 1.33125200 |
| O | 0.90234500 | -0.66431400 | 0.96089200 |
| O | 1.79679300 | -1.78966800 | 1.03563900 |
| H | 2.28097400 | -1.57303000 | 1.84172400 |

trans-R5-R

| | | | |
|---|---|---|---|
| C | 3.36475900 | 0.43258600 | -0.14003600 |
| C | 2.54190900 | 1.64079000 | 0.22315600 |
| C | 1.99919200 | 0.61866400 | -0.73394300 |
| H | 4.21801300 | 0.57058100 | -0.79046500 |
| H | 3.48255300 | -0.33661500 | 0.61241000 |
| H | 2.83638300 | 2.60256400 | -0.17459200 |
| H | 2.11127900 | 1.66337800 | 1.21598500 |
| H | 1.95487300 | 0.89952700 | -1.78047500 |
| C | 0.85972800 | -0.26115100 | -0.33049400 |
| C | -0.51849300 | -0.06116900 | -0.88111700 |
| C | 0.28978200 | -1.29102300 | -1.24330500 |
| H | -0.58845700 | 0.71839900 | -1.63568200 |
| H | -0.01600200 | -2.22578100 | -0.78914500 |
| H | 0.72729900 | -1.37500000 | -2.22971500 |
| C | -1.72835500 | -0.19072300 | -0.00091700 |
| C | -2.24657500 | 1.06413200 | 0.74070900 |
| C | -3.12043800 | -0.30666800 | -0.66836900 |
| H | -1.59023000 | -1.00692000 | 0.71317800 |
| C | -3.68534000 | 0.55910000 | 0.48244400 |
| H | -2.03608800 | 1.96572900 | 0.15888900 |
| H | -1.92051800 | 1.21663400 | 1.76955600 |
| H | -3.13767000 | 0.24083600 | -1.61459100 |
| H | -3.53459600 | -1.30305000 | -0.82399700 |
| H | -4.45056300 | 1.29554300 | 0.23890600 |
| H | -4.03827600 | -0.06318000 | 1.30673100 |
| O | 0.88113400 | -0.58710300 | 1.06458700 |
| O | 1.54949000 | -1.67413100 | 1.32719200 |

trans-R5-TS

| | | | |
|---|---|---|---|
| C | 0.83677600 | 0.59769200 | -0.12303500 |
| C | 0.16293600 | 1.91324000 | -0.32576200 |
| H | -0.09255600 | 2.16358200 | -1.34862800 |
| H | 0.47917600 | 2.74104200 | 0.29594500 |
| C | 1.92465400 | 0.36541700 | 0.89683300 |
| C | 2.58292200 | -0.95519600 | 0.82000600 |
| C | 3.33986000 | 0.23186400 | 0.36265400 |
| H | 1.74382700 | 0.81219000 | 1.86889700 |
| H | 1.87921500 | -1.54204400 | -0.22855300 |
| H | 2.83056200 | -1.61451800 | 1.64123900 |
| H | 4.17596700 | 0.56833100 | 0.96479500 |
| H | 3.44355900 | 0.38971700 | -0.70646000 |
| O | 1.10088300 | -0.09517100 | -1.31074700 |
| O | 1.07393600 | -1.46609400 | -1.04113400 |
| C | -0.59289200 | 0.75661300 | 0.29673100 |

| | | | |
|---|---|---|---|
| H | -0.74727000 | 0.81499400 | 1.37191500 |
| C | -1.68716500 | 0.06443200 | -0.47179100 |
| C | -2.12534300 | -1.27044200 | 0.18406300 |
| C | -3.09694300 | 0.62891500 | -0.14947300 |
| H | -1.41442900 | 0.01209900 | -1.52634300 |
| C | -3.28295700 | -0.51314500 | 0.87895300 |
| H | -1.38550400 | -1.77092000 | 0.81023500 |
| H | -2.49207800 | -1.97623900 | -0.56252300 |
| H | -3.14662000 | 1.65736200 | 0.21323400 |
| H | -3.77315100 | 0.51757600 | -0.99875500 |
| H | -3.01408400 | -0.19721100 | 1.88864200 |
| H | -4.25625600 | -1.00218300 | 0.91634200 |

trans-R5-P

| | | | |
|---|---|---|---|
| C | 2.29067700 | 1.84025100 | 0.30223700 |
| C | 1.97862700 | 0.77183900 | -0.65804200 |
| C | 3.26265300 | 0.75045900 | 0.16259000 |
| H | 2.38058100 | 2.90924400 | 0.18865400 |
| H | 2.06880800 | 1.00746300 | -1.71640900 |
| H | 3.30948800 | 0.00725200 | 0.95178500 |
| H | 4.20496600 | 0.93835700 | -0.34281100 |
| C | 0.88051000 | -0.21387100 | -0.36515000 |
| C | -0.50774200 | 0.07169300 | -0.86847400 |
| C | 0.30291400 | -1.05548300 | -1.46285600 |
| H | -0.57739400 | 0.98128000 | -1.46032200 |
| H | -0.00341800 | -2.06071200 | -1.19777400 |
| H | 0.72282200 | -0.94853100 | -2.45478200 |
| C | -1.71701600 | -0.21413100 | -0.02700700 |
| C | -2.21385900 | 0.87604000 | 0.95118100 |
| C | -3.11371600 | -0.17591900 | -0.69485000 |
| H | -1.58749400 | -1.15587900 | 0.51200800 |
| C | -3.66119800 | 0.45221000 | 0.60863900 |
| H | -1.99079900 | 1.87122400 | 0.55690800 |
| H | -1.87872800 | 0.81876000 | 1.98663700 |
| H | -3.12618700 | 0.54906800 | -1.51318700 |
| H | -3.54487000 | -1.11498600 | -1.04334900 |
| H | -4.41734700 | 1.23205600 | 0.52146700 |
| H | -4.01976200 | -0.31644100 | 1.29587800 |
| O | 0.90187800 | -0.76639900 | 0.91794200 |
| O | 1.86692000 | -1.82623100 | 0.95096700 |
| H | 1.29784700 | -2.59564700 | 1.07443100 |

trans-R6-R

| | | | |
|---|---|---|---|
| C | -3.17892000 | -1.20018600 | 0.32045300 |
| C | -2.29317000 | -2.28396200 | -0.23273400 |

| | | | |
|---|---|---|---|
| C | -1.89868100 | -0.84089200 | -0.37861100 |
| H | -4.11074600 | -0.98934400 | -0.18625100 |
| H | -3.20642200 | -1.06927200 | 1.39494700 |
| H | -2.62471700 | -2.81322200 | -1.11601700 |
| H | -1.73085800 | -2.88462900 | 0.47147600 |
| H | -1.99737900 | -0.40762300 | -1.36710100 |
| C | -0.75808100 | -0.31418600 | 0.42898000 |
| C | 0.49421500 | 0.20587600 | -0.23958100 |
| C | -0.45144600 | 1.15426900 | 0.41698300 |
| H | -0.61924500 | -0.79791700 | 1.39259800 |
| H | 0.44976700 | 0.21782000 | -1.32657700 |
| H | -0.19020100 | 1.68420200 | 1.32484200 |
| C | 1.85340800 | -0.03115200 | 0.34987600 |
| C | 2.64662600 | -1.28533600 | -0.09090600 |
| C | 3.04637300 | 0.82811400 | -0.13588100 |
| H | 1.79607000 | 0.00152600 | 1.44341400 |
| C | 3.93477000 | -0.43246600 | -0.02045200 |
| H | 2.40232000 | -1.54401700 | -1.12449000 |
| H | 2.57885700 | -2.18090600 | 0.52702700 |
| H | 2.90603000 | 1.11604100 | -1.18094700 |
| H | 3.31492900 | 1.71085100 | 0.44457800 |
| H | 4.69569400 | -0.59262500 | -0.78346900 |
| H | 4.39864700 | -0.49914000 | 0.96536600 |
| O | -1.21175000 | 1.95461300 | -0.47697200 |
| O | -2.34241500 | 2.32635900 | 0.05345200 |

trans-R6-TS

| | | | |
|---|---|---|---|
| C | -0.68084900 | -0.44388300 | -0.45579500 |
| C | -0.05905900 | 0.94249800 | -0.66561600 |
| H | -0.37128900 | -1.18136700 | -1.19273400 |
| H | 0.60631000 | 1.09492600 | -1.50572200 |
| C | -2.08891600 | -0.44757300 | -0.03516300 |
| H | -2.24060400 | 0.89088400 | 0.29200600 |
| O | -0.90266900 | 2.04938900 | -0.57692900 |
| O | -1.76598300 | 1.92648600 | 0.49744500 |
| C | -3.18637600 | -1.27599300 | -0.57026000 |
| H | -4.14619800 | -0.81345800 | -0.76807200 |
| H | -2.92876900 | -2.08911400 | -1.24107100 |
| C | -2.77031400 | -1.38409100 | 0.89418100 |
| H | -2.21898300 | -2.26300900 | 1.21099600 |
| H | -3.46364700 | -0.99205000 | 1.62862700 |
| C | 1.74920800 | -0.46908300 | 0.55176600 |
| C | 2.43803700 | -1.01154200 | -0.72582700 |
| C | 2.96330600 | 0.47105000 | 0.74322500 |
| H | 1.77049200 | -1.25151300 | 1.31664500 |

| | | | |
|---|---|---|---|
| C | 3.77751400 | -0.48317100 | -0.15942100 |
| H | 2.14907000 | -0.44770100 | -1.61660700 |
| H | 2.34949200 | -2.07738100 | -0.93831300 |
| H | 2.78252200 | 1.42702000 | 0.24476900 |
| H | 3.31579900 | 0.65599600 | 1.75786600 |
| H | 4.48726500 | -0.04455800 | -0.85991400 |
| H | 4.28416900 | -1.24921200 | 0.43007500 |
| C | 0.36218500 | 0.11777200 | 0.49493400 |
| H | -0.05482900 | 0.43764300 | 1.44517500 |

trans-R6-P

| | | | |
|---|---|---|---|
| C | -3.37234100 | -1.18620400 | 0.23799300 |
| C | -1.92055000 | -1.32277200 | 0.07863900 |
| C | -2.68774800 | -0.95935100 | -1.11469600 |
| H | -3.98769600 | -2.07416500 | 0.33905200 |
| H | -3.74252100 | -0.31106400 | 0.76494300 |
| H | -2.62445300 | 0.05591500 | -1.49123100 |
| H | -2.85273600 | -1.72210100 | -1.86970700 |
| C | -0.77167700 | -0.74428400 | 0.77286100 |
| C | 0.35049400 | -0.09737000 | -0.00062300 |
| C | -0.55123500 | 0.76253500 | 0.82340400 |
| H | -0.49946900 | -1.23818600 | 1.70134500 |
| H | 0.15967900 | -0.02613200 | -1.06885200 |
| H | -0.16264800 | 1.19426400 | 1.74272800 |
| C | 1.78476200 | -0.26901900 | 0.39935100 |
| C | 2.61710900 | -1.39971700 | -0.25358000 |
| C | 2.82788400 | 0.73878800 | -0.14228100 |
| H | 1.86469800 | -0.33547600 | 1.49010200 |
| C | 3.82658500 | -0.43596300 | -0.26299700 |
| H | 2.26036800 | -1.59374600 | -1.26833200 |
| H | 2.70809700 | -2.34609700 | 0.27990300 |
| H | 2.52319200 | 1.10454600 | -1.12607600 |
| H | 3.09529300 | 1.58884900 | 0.48582900 |
| H | 4.48959800 | -0.45669300 | -1.12746900 |
| H | 4.42262500 | -0.54234400 | 0.64544600 |
| O | -1.52358200 | 1.52435000 | 0.19031900 |
| O | -0.85544500 | 2.57883000 | -0.51873700 |
| H | -1.24151600 | 3.35713100 | -0.09875800 |

trans-R7-R

| | | | |
|---|---|---|---|
| C | 3.50902400 | -0.74964900 | -0.22677500 |
| C | 2.71839000 | -2.03070100 | -0.23887700 |
| C | 2.19038800 | -0.82597900 | 0.48843500 |
| H | 4.41225100 | -0.71399700 | 0.36729700 |
| H | 3.54161200 | -0.16670100 | -1.13863300 |

| | | | |
|---|---|---|---|
| H | 3.08612900 | -2.86266000 | 0.34660300 |
| H | 2.22192600 | -2.30789300 | -1.16057700 |
| H | 2.22872300 | -0.86251700 | 1.57251600 |
| C | 1.01275800 | -0.09761100 | -0.06900500 |
| C | -0.32996000 | -0.11237100 | 0.62703000 |
| H | 0.95999600 | -0.05731200 | -1.15440600 |
| H | -0.36507600 | -0.62839800 | 1.58353900 |
| C | -1.60024100 | -0.18546600 | -0.17289300 |
| C | -2.22512600 | -1.57749000 | -0.43200600 |
| C | -2.93214200 | 0.23042700 | 0.49562500 |
| H | -1.48347700 | 0.35133900 | -1.11789500 |
| C | -3.61752500 | -0.92215100 | -0.27498400 |
| H | -2.01330300 | -2.25298200 | 0.40116900 |
| H | -1.98750200 | -2.08083400 | -1.36952700 |
| H | -2.90310400 | 0.02193000 | 1.56861100 |
| H | -3.28428600 | 1.24962500 | 0.33722300 |
| H | -4.39387100 | -1.48969600 | 0.23715200 |
| H | -4.00202400 | -0.57859600 | -1.23692300 |
| C | 0.50398500 | 1.12905500 | 0.61891400 |
| H | 0.99190000 | 1.49939900 | 1.51215300 |
| O | 0.00839900 | 2.18321200 | -0.19629200 |
| O | 0.94320000 | 3.03315200 | -0.50884300 |

trans-R7-TS

| | | | |
|---|---|---|---|
| C | 3.62138000 | -0.27708600 | -0.43202900 |
| C | 2.98440800 | -1.61142600 | -0.14701200 |
| C | 2.42576000 | -0.36175600 | 0.47339900 |
| H | 4.58608000 | -0.06863200 | 0.01073600 |
| H | 3.46797900 | 0.15515300 | -1.41279600 |
| H | 3.51836100 | -2.30590600 | 0.48770300 |
| H | 2.40413700 | -2.07069700 | -0.93786900 |
| H | 2.61203600 | -0.22742700 | 1.53444900 |
| C | 1.10918300 | 0.15785000 | -0.00296300 |
| C | -0.13289500 | -0.00075000 | 0.85615900 |
| H | 0.92411600 | 0.06431900 | -1.06944300 |
| H | -0.01316600 | -0.40624100 | 1.85844500 |
| C | -1.42897900 | -0.22742900 | 0.18280400 |
| C | -1.76866200 | -1.40457500 | -0.73712700 |
| C | -2.77510000 | -0.35923600 | 0.88815000 |
| H | -1.41366000 | 0.84459500 | -0.63065600 |
| C | -3.24655800 | -1.24370900 | -0.29474500 |
| H | -1.31845400 | -2.33108500 | -0.36446600 |
| H | -1.54237100 | -1.30756600 | -1.80066900 |
| H | -2.68010600 | -0.92738200 | 1.82059800 |
| H | -3.33241600 | 0.55606400 | 1.09394200 |

| | | | |
|---|---|---|---|
| H | -3.78750800 | -2.15549600 | -0.04565800 |
| H | -3.82371300 | -0.67055100 | -1.02057800 |
| C | 0.52174800 | 1.36511200 | 0.63726700 |
| H | 1.03953600 | 1.84317500 | 1.45923100 |
| O | -0.21897800 | 2.32915800 | -0.04657800 |
| O | -0.84384200 | 1.76905500 | -1.14421900 |

trans-R7-P

| | | | |
|---|---|---|---|
| C | 2.94263200 | -1.74576600 | -0.44719700 |
| C | 2.30004300 | -0.71661900 | 0.43906400 |
| C | 3.49396400 | -0.34544300 | -0.39419600 |
| H | 3.51631200 | -2.53711800 | 0.01650900 |
| H | 2.39833500 | -2.03986900 | -1.33595100 |
| H | 2.46823200 | -0.82639600 | 1.50573900 |
| H | 3.31494700 | 0.30178300 | -1.24376000 |
| H | 4.44100000 | -0.19069800 | 0.10538400 |
| C | 0.96294100 | -0.16676600 | 0.06910100 |
| C | -0.26206200 | -0.44756300 | 0.90495500 |
| C | 0.36471300 | 0.94717300 | 0.86104600 |
| H | 0.76625600 | -0.09299000 | -0.99799700 |
| H | -0.09423500 | -0.95401800 | 1.85208200 |
| H | 0.89434600 | 1.31113900 | 1.73821100 |
| C | -1.54692000 | -0.71088100 | 0.26190900 |
| C | -2.01072400 | -0.51734600 | -1.15518300 |
| C | -2.94372800 | -0.74073000 | 0.81964500 |
| C | -3.45851500 | -0.78343800 | -0.64785500 |
| H | -1.62207900 | -1.21845300 | -1.90110500 |
| H | -1.85692800 | 0.50238400 | -1.52810900 |
| H | -3.21192600 | -1.58843000 | 1.45783900 |
| H | -3.21843100 | 0.18174000 | 1.34767500 |
| H | -3.83214200 | -1.76580300 | -0.93499200 |
| H | -4.19799600 | -0.02882800 | -0.91205900 |
| O | -0.39583000 | 1.89864800 | 0.19515400 |
| O | 0.49717000 | 2.69433400 | -0.59911600 |
| H | 0.36954500 | 3.56596200 | -0.20550100 |

trans-R8-R

| | | | |
|---|---|---|---|
| C | -2.93682900 | -1.16751900 | -0.83341800 |
| C | -2.36565600 | 0.04612600 | -0.15788600 |
| C | -3.68779200 | -0.50783300 | 0.29272400 |
| H | -3.32142700 | -1.05826800 | -1.83840300 |
| H | -2.45966500 | -2.11848800 | -0.63043400 |
| H | -2.39347800 | 0.96975800 | -0.72400100 |
| H | -3.71429900 | -1.01851200 | 1.24739000 |
| H | -4.58368600 | 0.04826800 | 0.05127400 |

| | | | |
|---|---|---|---|
| C | -1.17614600 | -0.11132500 | 0.73216600 |
| C | 0.17648800 | 0.37164000 | 0.30406200 |
| C | -0.55063000 | 1.10277200 | 1.38264900 |
| H | -1.16182700 | -1.03523800 | 1.30458400 |
| H | -0.15698200 | 1.03060300 | 2.38837500 |
| H | -0.96788000 | 2.06573300 | 1.11462500 |
| C | 1.41817000 | -0.42926200 | 0.56006200 |
| C | 1.85797900 | -1.48859600 | -0.47847200 |
| C | 2.81258400 | 0.22644700 | 0.41314600 |
| H | 1.32323300 | -0.88775100 | 1.54920100 |
| C | 3.32365700 | -1.12339800 | -0.14435400 |
| H | 1.59250900 | -1.16469200 | -1.48686700 |
| H | 1.52910700 | -2.51719800 | -0.32880800 |
| H | 2.80114000 | 0.99328400 | -0.36342100 |
| H | 3.27993600 | 0.63216200 | 1.31022700 |
| H | 4.03850300 | -1.09376200 | -0.96564200 |
| H | 3.71778800 | -1.75821600 | 0.65153700 |
| O | 0.17697000 | 0.99744300 | -0.98527900 |
| O | 0.72879000 | 2.17730700 | -0.97768400 |

trans-R8-TS

| | | | |
|---|---|---|---|
| C | -0.18789000 | -0.44196300 | 0.34759800 |
| C | 1.22359200 | -0.12896700 | 0.72272700 |
| H | 1.32608700 | 0.62891600 | 1.49583400 |
| C | -1.37184700 | 0.32143000 | 0.91247000 |
| C | -1.61336400 | 1.69952800 | 0.23468600 |
| C | -2.69771400 | -0.16038900 | 0.31537000 |
| H | -1.31305800 | 0.32320600 | 2.00323400 |
| C | -2.87545100 | 1.13635900 | -0.47171500 |
| H | -0.80736500 | 2.01989500 | -0.42719600 |
| H | -1.83780600 | 2.49088500 | 0.94960700 |
| H | -2.28180300 | -1.11363700 | -0.51438000 |
| H | -3.48630400 | -0.60092500 | 0.92158400 |
| H | -2.75074900 | 1.01380000 | -1.54980600 |
| H | -3.80848800 | 1.67016100 | -0.28523100 |
| O | -0.38951500 | -0.73391200 | -1.00829300 |
| O | -1.39986900 | -1.68907900 | -1.09549600 |
| C | 0.59005400 | -1.43702800 | 1.15028900 |
| H | 0.90315000 | -2.32580100 | 0.61631700 |
| H | 0.32693700 | -1.57956900 | 2.19065800 |
| C | 2.30163100 | -0.10674500 | -0.31116700 |
| C | 2.89324300 | 1.21276700 | -0.71791500 |
| C | 3.70154000 | 0.24462100 | 0.10546400 |
| H | 2.19338600 | -0.84850500 | -1.09307300 |
| H | 2.51678700 | 2.09979700 | -0.22280600 |

| | | | |
|---|---|---|---|
| H | 3.16400600 | 1.35275400 | -1.75571800 |
| H | 4.52282000 | -0.27084900 | -0.37414600 |
| H | 3.86470500 | 0.48611900 | 1.14858600 |

trans-R8-P

| | | | |
|---|---|---|---|
| C | 2.92001400 | 1.16473200 | -0.84005600 |
| C | 2.34281000 | -0.07338500 | -0.21571300 |
| C | 3.69324300 | 0.42269900 | 0.21844400 |
| H | 3.26717200 | 1.10631800 | -1.86284100 |
| H | 2.47535500 | 2.11340900 | -0.56463500 |
| H | 2.32122200 | -0.96126400 | -0.83509700 |
| H | 3.76555500 | 0.87689400 | 1.19904200 |
| H | 4.56685500 | -0.13771600 | -0.08666000 |
| C | 1.19078800 | 0.05930900 | 0.72480600 |
| C | -0.19121600 | -0.38103800 | 0.32312300 |
| C | 0.56672500 | -1.16098700 | 1.35457200 |
| H | 1.21170800 | 0.95913100 | 1.33486100 |
| H | 0.22703100 | -1.11233800 | 2.38140100 |
| H | 0.94086100 | -2.12305100 | 1.02750100 |
| C | -1.38738100 | 0.46407000 | 0.68558100 |
| C | -1.76083600 | 1.61501000 | -0.29578800 |
| C | -2.77043200 | -0.09180900 | 0.45408000 |
| H | -1.25416800 | 0.82532200 | 1.71485300 |
| C | -3.24227100 | 1.14236300 | -0.26217300 |
| H | -1.29876500 | 1.46051000 | -1.27119400 |
| H | -1.56272500 | 2.62937400 | 0.04844400 |
| H | -3.31038100 | -0.88729000 | 0.95478300 |
| H | -3.73720800 | 1.00693100 | -1.22765000 |
| H | -3.88483400 | 1.78120900 | 0.35568700 |
| O | -0.24412100 | -0.93889200 | -0.95898500 |
| O | -1.00454800 | -2.14648700 | -0.91638100 |
| H | -1.90699100 | -1.82018600 | -1.04682800 |

trans-R9-R

| | | | |
|---|---|---|---|
| C | -2.93682900 | -1.16751900 | -0.83341800 |
| C | -2.36565600 | 0.04612600 | -0.15788600 |
| C | -3.68779200 | -0.50783300 | 0.29272400 |
| H | -3.32142700 | -1.05826800 | -1.83840300 |
| H | -2.45966500 | -2.11848800 | -0.63043400 |
| H | -2.39347800 | 0.96975800 | -0.72400100 |
| H | -3.71429900 | -1.01851200 | 1.24739000 |
| H | -4.58368600 | 0.04826800 | 0.05127400 |
| C | -1.17614600 | -0.11132500 | 0.73216600 |
| C | 0.17648800 | 0.37164000 | 0.30406200 |
| C | -0.55063000 | 1.10277200 | 1.38264900 |

| | | | |
|---|---|---|---|
| H | -1.16182700 | -1.03523800 | 1.30458400 |
| H | -0.15698200 | 1.03060300 | 2.38837500 |
| H | -0.96788000 | 2.06573300 | 1.11462500 |
| C | 1.41817000 | -0.42926200 | 0.56006200 |
| C | 1.85797900 | -1.48859600 | -0.47847200 |
| C | 2.81258400 | 0.22644700 | 0.41314600 |
| H | 1.32323300 | -0.88775100 | 1.54920100 |
| C | 3.32365700 | -1.12339800 | -0.14435400 |
| H | 1.59250900 | -1.16469200 | -1.48686700 |
| H | 1.52910700 | -2.51719800 | -0.32880800 |
| H | 2.80114000 | 0.99328400 | -0.36342100 |
| H | 3.27993600 | 0.63216200 | 1.31022700 |
| H | 4.03850300 | -1.09376200 | -0.96564200 |
| H | 3.71778800 | -1.75821600 | 0.65153700 |
| O | 0.17697000 | 0.99744300 | -0.98527900 |
| O | 0.72879000 | 2.17730700 | -0.97768400 |

trans-R9-TS

| | | | |
|---|---|---|---|
| C | -0.23131800 | 0.53402600 | 0.17002500 |
| C | 1.16406000 | 0.73060300 | -0.34062600 |
| H | 1.24493800 | 0.93489300 | -1.40492500 |
| C | -1.43995100 | 0.48935800 | -0.74724800 |
| C | -2.02842400 | -0.92059900 | -0.85155700 |
| C | -2.76577100 | 0.95042600 | -0.07814300 |
| H | -1.18949900 | 0.97058500 | -1.69549600 |
| C | -3.28670100 | -0.51151200 | -0.08892900 |
| H | -1.22604300 | -1.59741900 | -0.03588500 |
| H | -2.05245100 | -1.47707900 | -1.78613000 |
| H | -2.63640000 | 1.39336800 | 0.90997600 |
| H | -3.34973900 | 1.62229400 | -0.70673700 |
| H | -3.36379000 | -0.95647400 | 0.90542500 |
| H | -4.22476800 | -0.67930200 | -0.62037900 |
| O | -0.40495900 | -0.32604900 | 1.26317500 |
| O | -0.35121200 | -1.63585500 | 0.78823000 |
| C | 2.29253800 | -0.05957800 | 0.23321700 |
| C | 3.00942100 | -1.04900700 | -0.63673000 |
| C | 3.69505800 | 0.22796300 | -0.22739200 |
| H | 2.16425700 | -0.33797100 | 1.27241800 |
| H | 2.68001700 | -1.13605800 | -1.66483200 |
| H | 3.33700700 | -1.97593000 | -0.18609400 |
| H | 4.49483400 | 0.16898500 | 0.49882300 |
| H | 3.82325500 | 0.99410600 | -0.98226800 |
| C | 0.48616600 | 1.80066900 | 0.49336700 |
| H | 0.83349600 | 1.90713800 | 1.51387500 |
| H | 0.14377900 | 2.71000100 | 0.01508600 |

trans-R9-P

| | | | |
|---|---|---|---|
| C | -3.20930800 | -0.50841800 | -1.16409900 |
| C | -2.45896700 | 0.18829400 | -0.06529000 |
| C | -3.80500300 | -0.41846300 | 0.21613300 |
| H | -3.64272000 | 0.09633300 | -1.94925900 |
| H | -2.83581000 | -1.47231800 | -1.48750600 |
| H | -2.41125400 | 1.26841100 | -0.12290800 |
| H | -3.82909500 | -1.32189800 | 0.81310500 |
| H | -4.64446800 | 0.24690900 | 0.36749500 |
| C | -1.24496200 | -0.46218400 | 0.51307900 |
| C | 0.13358100 | 0.08281500 | 0.25956300 |
| C | -0.47538000 | 0.20630800 | 1.62643100 |
| H | -1.29527700 | -1.54732400 | 0.54929200 |
| H | -0.05819400 | -0.40241800 | 2.41962800 |
| H | -0.80016800 | 1.19896500 | 1.91298800 |
| C | 1.32968200 | -0.82914500 | 0.06096300 |
| C | 2.14652100 | -0.67172500 | -1.19266600 |
| C | 2.63711800 | -0.54228400 | 0.86553900 |
| H | 0.99966000 | -1.86618000 | 0.21502500 |
| C | 3.45405900 | -0.66521400 | -0.45371300 |
| H | 1.89385800 | -0.79506400 | -2.23792700 |
| H | 2.64017500 | 0.46700800 | 1.27491400 |
| H | 2.88166100 | -1.25773600 | 1.65038400 |
| H | 4.15448200 | 0.14568300 | -0.67544500 |
| H | 4.00782300 | -1.60860800 | -0.53209600 |
| O | 0.09998200 | 1.22992600 | -0.53719400 |
| O | 1.16216000 | 2.09688800 | -0.13120600 |
| H | 1.79815200 | 1.95382400 | -0.84612200 |

trans-R10-R

| | | | |
|---|---|---|---|
| C | -3.21029800 | -1.21210200 | 0.61651000 |
| C | -2.65974200 | -0.22997000 | -0.37896600 |
| C | -3.88968000 | 0.11646100 | 0.41073000 |
| H | -3.70530700 | -2.09986400 | 0.24585600 |
| H | -2.64933300 | -1.35245100 | 1.53237800 |
| H | -2.81261000 | -0.46894300 | -1.42716400 |
| H | -3.78218700 | 0.86142600 | 1.18927700 |
| H | -4.84368900 | 0.12668800 | -0.09931800 |
| C | -1.37481100 | 0.47183900 | -0.08352600 |
| C | -0.13396000 | 0.09814900 | -0.84319900 |
| C | -0.78857600 | 1.43316200 | -1.08416500 |
| H | -1.22756800 | 0.73800700 | 0.95898700 |
| H | -0.28858800 | 2.31321000 | -0.70151900 |
| H | -1.31939000 | 1.56796800 | -2.01845100 |

| | | | |
|---|---|---|---|
| C | 1.20330000 | -0.01201600 | -0.17022900 |
| C | 1.61491800 | -1.37929600 | 0.42063300 |
| C | 2.47495400 | -0.01649500 | -1.04875100 |
| C | 2.98586600 | -1.26778600 | -0.29186700 |
| H | 0.97887900 | -2.18112600 | 0.04298200 |
| H | 1.63486800 | -1.41180800 | 1.50971400 |
| H | 2.23139900 | -0.19831500 | -2.09641600 |
| H | 3.08347500 | 0.88368500 | -0.96662600 |
| H | 3.26969400 | -2.11642100 | -0.91277400 |
| H | 3.79447700 | -1.03407700 | 0.39816200 |
| H | -0.26588300 | -0.64879400 | -1.62004300 |
| O | 1.23345300 | 1.04570800 | 0.82917100 |
| O | 2.33778900 | 1.08043300 | 1.50607100 |

trans-R10-TS

| | | | |
|---|---|---|---|
| C | -0.82106000 | -0.61802600 | -1.34575600 |
| C | 0.00386600 | -0.79499500 | -0.08221600 |
| C | -1.15408500 | 0.12703400 | -0.10683600 |
| H | -1.47322500 | -1.42582000 | -1.65908500 |
| H | -0.09193400 | -1.71710700 | 0.48405300 |
| H | -0.51889900 | 1.32075800 | -0.11841800 |
| C | 1.38520100 | -0.16709800 | -0.05471600 |
| C | 2.11143200 | -0.01583900 | 1.29785700 |
| C | 2.60536100 | -0.92438200 | -0.60354900 |
| C | 3.43728300 | -0.35318900 | 0.57164200 |
| H | 1.81175400 | -0.80048100 | 1.99540800 |
| H | 2.00712000 | 0.96024800 | 1.76968800 |
| H | 2.45636500 | -2.00245000 | -0.52262400 |
| H | 2.89921300 | -0.66764100 | -1.62147400 |
| H | 4.11980600 | -1.03698500 | 1.07405800 |
| H | 3.97564700 | 0.54766700 | 0.28017500 |
| O | 1.33931200 | 1.06944400 | -0.76153000 |
| O | 0.48406500 | 1.94507500 | -0.10616000 |
| H | -0.33374500 | -0.07599800 | -2.14987200 |
| C | -2.38585800 | 0.08641800 | 0.69997100 |
| C | -3.69162200 | 0.51644900 | 0.08510600 |
| C | -3.46733800 | -0.93156800 | 0.39089500 |
| H | -2.24934300 | 0.31576700 | 1.75058400 |
| H | -3.65413200 | 0.81549200 | -0.95482400 |
| H | -4.38424900 | 1.07261500 | 0.70216300 |
| H | -3.98405000 | -1.36514600 | 1.23675300 |
| H | -3.30643200 | -1.60590800 | -0.43945700 |

trans-R10-P

| | | | |
|---|---|---|---|
| C | 1.02230500 | -1.47997300 | 1.11691100 |
| C | -0.01297900 | -1.35203500 | -0.00331900 |

| | | | |
|---|---|---|---|
| C | 1.40782600 | -1.07806000 | -0.24439400 |
| H | 1.25393600 | -2.48367600 | 1.46001700 |
| H | -0.40915300 | -2.29124500 | -0.38538300 |
| C | -1.06937000 | -0.27015000 | 0.01348900 |
| C | -1.74224300 | 0.05369100 | -1.34431300 |
| C | -2.45362200 | -0.64996500 | 0.59686100 |
| C | -3.13430000 | -0.09697100 | -0.68091600 |
| H | -1.52688300 | -0.71263300 | -2.09102500 |
| H | -1.49851900 | 1.03090900 | -1.76336900 |
| H | -2.55948000 | -1.72895900 | 0.72081500 |
| H | -2.70064200 | -0.15108600 | 1.53302700 |
| H | -3.82908800 | -0.77008100 | -1.18219800 |
| H | -3.62093600 | 0.86148000 | -0.50722500 |
| H | 1.00023200 | -0.72257800 | 1.89608600 |
| C | 2.17994400 | 0.03189800 | -0.78563600 |
| C | 2.62407000 | 1.16266600 | 0.12674600 |
| C | 3.62907000 | 0.17832000 | -0.37452700 |
| H | 1.93835900 | 0.31674100 | -1.80326400 |
| H | 2.31100800 | 1.10153600 | 1.16012700 |
| H | 2.62512500 | 2.15952600 | -0.29509900 |
| H | 4.33522000 | 0.49783700 | -1.12933200 |
| H | 4.00175400 | -0.55923300 | 0.32481800 |
| O | -0.45928900 | 0.84400800 | 0.65438900 |
| O | -1.41777900 | 1.89093700 | 0.76044100 |
| H | -1.00859400 | 2.57538800 | 0.21795600 |

trans-R11-R

| | | | |
|---|---|---|---|
| C | -3.21029800 | -1.21210200 | 0.61651000 |
| C | -2.65974200 | -0.22997000 | -0.37896600 |
| C | -3.88968000 | 0.11646100 | 0.41073000 |
| H | -3.70530700 | -2.09986400 | 0.24585600 |
| H | -2.64933300 | -1.35245100 | 1.53237800 |
| H | -2.81261000 | -0.46894300 | -1.42716400 |
| H | -3.78218700 | 0.86142600 | 1.18927700 |
| H | -4.84368900 | 0.12668800 | -0.09931800 |
| C | -1.37481100 | 0.47183900 | -0.08352600 |
| C | -0.13396000 | 0.09814900 | -0.84319900 |
| C | -0.78857600 | 1.43316200 | -1.08416500 |
| H | -1.22756800 | 0.73800700 | 0.95898700 |
| H | -0.28858800 | 2.31321000 | -0.70151900 |
| H | -1.31939000 | 1.56796800 | -2.01845100 |
| C | 1.20330000 | -0.01201600 | -0.17022900 |
| C | 1.61491800 | -1.37929600 | 0.42063300 |
| C | 2.47495400 | -0.01649500 | -1.04875100 |

| | | | |
|---|---|---|---|
| C | 2.98586600 | -1.26778600 | -0.29186700 |
| H | 0.97887900 | -2.18112600 | 0.04298200 |
| H | 1.63486800 | -1.41180800 | 1.50971400 |
| H | 2.23139900 | -0.19831500 | -2.09641600 |
| H | 3.08347500 | 0.88368500 | -0.96662600 |
| H | 3.26969400 | -2.11642100 | -0.91277400 |
| H | 3.79447700 | -1.03407700 | 0.39816200 |
| H | -0.26588300 | -0.64879400 | -1.62004300 |
| O | 1.23345300 | 1.04570800 | 0.82917100 |
| O | 2.33778900 | 1.08043300 | 1.50607100 |

trans-R11-TS

| | | | |
|---|---|---|---|
| C | 3.15854100 | -1.26942500 | -0.41202700 |
| C | 2.50078300 | -0.25235400 | 0.48148100 |
| C | 3.76063700 | 0.10047100 | -0.25807600 |
| H | 3.66564700 | -2.10535100 | 0.05095600 |
| H | 2.66082000 | -1.49788600 | -1.34644900 |
| H | 2.59612600 | -0.41354800 | 1.55042500 |
| H | 3.66720800 | 0.78742100 | -1.08982200 |
| H | 4.67757600 | 0.19327700 | 0.30828100 |
| C | 1.20618600 | 0.35627300 | 0.05318900 |
| C | -0.09887200 | -0.01177200 | 0.73451900 |
| C | 0.50807800 | 1.31838300 | 0.93961200 |
| H | 1.10430400 | 0.52172800 | -1.01910200 |
| H | -0.37537600 | 2.12374200 | 0.22078200 |
| H | 0.88317100 | 1.72416100 | 1.87106200 |
| C | -1.34990000 | -0.05710500 | -0.14077900 |
| C | -1.62925600 | -1.41443400 | -0.80070200 |
| C | -2.65492700 | -0.19208600 | 0.66842800 |
| C | -2.62068100 | -1.70908700 | 0.35441200 |
| H | -0.77508900 | -2.07740600 | -0.93928500 |
| H | -2.14672800 | -1.26654300 | -1.74969800 |
| H | -2.63108400 | 0.12529700 | 1.71052700 |
| H | -3.46346500 | 0.31859100 | 0.14388500 |
| H | -2.15206300 | -2.28237500 | 1.15523300 |
| H | -3.56370500 | -2.18206400 | 0.08665900 |
| H | -0.09502400 | -0.72640600 | 1.55420500 |
| O | -1.28419800 | 0.98437600 | -1.09399300 |
| O | -1.30778400 | 2.20864700 | -0.43450800 |

trans-R11-P

| | | | |
|---|---|---|---|
| C | 3.19734100 | 1.13215200 | 0.67957100 |
| C | 2.71202800 | 0.18409500 | -0.37994900 |
| C | 3.91350800 | -0.17308200 | 0.44670800 |
| H | 3.68864000 | 2.04480000 | 0.36928100 |
| H | 2.59415500 | 1.22095000 | 1.57504200 |

| | | | |
|---|---|---|---|
| H | 2.90026700 | 0.46400600 | -1.41118700 |
| H | 3.78962000 | -0.95232400 | 1.18844900 |
| H | 4.88787100 | -0.14359900 | -0.02251700 |
| C | 1.42643500 | -0.55409000 | -0.16177000 |
| C | 0.17747500 | -0.08360900 | -0.90243700 |
| C | 0.81985500 | -1.36511100 | -1.23008600 |
| H | 1.27205600 | -0.90801400 | 0.85688400 |
| H | 0.34973100 | 0.74682000 | -1.58579600 |
| H | 0.42642500 | -2.36953500 | -1.21586400 |
| C | -1.16280700 | 0.00826200 | -0.21385100 |
| C | -1.43148200 | 1.29967800 | 0.59752600 |
| C | -2.39848200 | 0.31631100 | -1.09644000 |
| C | -2.78846800 | 1.46502800 | -0.13212900 |
| H | -0.70443900 | 2.07882600 | 0.36137100 |
| H | -1.47341600 | 1.15690300 | 1.67657800 |
| H | -2.10971000 | 0.65667700 | -2.09214200 |
| H | -3.11167800 | -0.50239300 | -1.19878000 |
| H | -2.96703200 | 2.43475300 | -0.59545200 |
| H | -3.63335600 | 1.20747500 | 0.50465600 |
| O | -1.26446700 | -1.19118100 | 0.54717700 |
| O | -2.45509600 | -1.13688500 | 1.32472900 |
| H | -2.94505400 | -1.88861900 | 0.97136400 |

trans-R12-R

| | | | |
|---|---|---|---|
| C | -3.21029800 | -1.21210200 | 0.61651000 |
| C | -2.65974200 | -0.22997000 | -0.37896600 |
| C | -3.88968000 | 0.11646100 | 0.41073000 |
| H | -3.70530700 | -2.09986400 | 0.24585600 |
| H | -2.64933300 | -1.35245100 | 1.53237800 |
| H | -2.81261000 | -0.46894300 | -1.42716400 |
| H | -3.78218700 | 0.86142600 | 1.18927700 |
| H | -4.84368900 | 0.12668800 | -0.09931800 |
| C | -1.37481100 | 0.47183900 | -0.08352600 |
| C | -0.13396000 | 0.09814900 | -0.84319900 |
| C | -0.78857600 | 1.43316200 | -1.08416500 |
| H | -1.22756800 | 0.73800700 | 0.95898700 |
| H | -0.28858800 | 2.31321000 | -0.70151900 |
| H | -1.31939000 | 1.56796800 | -2.01845100 |
| C | 1.20330000 | -0.01201600 | -0.17022900 |
| C | 1.61491800 | -1.37929600 | 0.42063300 |
| C | 2.47495400 | -0.01649500 | -1.04875100 |
| C | 2.98586600 | -1.26778600 | -0.29186700 |
| H | 0.97887900 | -2.18112600 | 0.04298200 |
| H | 1.63486800 | -1.41180800 | 1.50971400 |

| | | | |
|---|---|---|---|
| H | 2.23139900 | -0.19831500 | -2.09641600 |
| H | 3.08347500 | 0.88368500 | -0.96662600 |
| H | 3.26969400 | -2.11642100 | -0.91277400 |
| H | 3.79447700 | -1.03407700 | 0.39816200 |
| H | -0.26588300 | -0.64879400 | -1.62004300 |
| O | 1.23345300 | 1.04570800 | 0.82917100 |
| O | 2.33778900 | 1.08043300 | 1.50607100 |

trans-R12-TS

| | | | |
|---|---|---|---|
| C | -1.16577800 | -0.05774700 | 0.20891600 |
| C | -1.78501600 | -0.60177400 | -1.10287800 |
| C | -3.07099400 | -0.72815100 | -0.28473600 |
| C | -2.24730100 | -0.86091700 | 0.98151600 |
| H | -1.37082500 | -1.57817000 | -1.36908000 |
| H | -1.77894600 | 0.06439200 | -1.96546100 |
| H | -3.98021300 | -1.26087900 | -0.55165900 |
| H | -3.30015600 | 0.56698800 | -0.22193200 |
| H | -1.89808600 | -1.88800300 | 1.13231600 |
| H | -2.62197000 | -0.44148200 | 1.91591800 |
| O | -1.47572200 | 1.33953700 | 0.36516300 |
| O | -2.72197200 | 1.64981200 | -0.19512300 |
| C | 0.28532500 | -0.18469500 | 0.53978400 |
| C | 1.05475800 | -1.41931500 | 0.15907000 |
| H | 0.56114500 | 0.27596600 | 1.48346900 |
| H | 0.54428700 | -2.19504800 | -0.39804800 |
| H | 1.78294500 | -1.78977300 | 0.86903800 |
| C | 2.58811000 | 0.65221100 | -0.35158700 |
| C | 3.49859000 | 0.38923800 | 0.81310700 |
| C | 3.91892900 | -0.00917500 | -0.57605700 |
| H | 2.51612900 | 1.69126200 | -0.65370900 |
| H | 3.24356700 | -0.39920700 | 1.50985500 |
| H | 3.98516800 | 1.24231500 | 1.26640300 |
| H | 4.70703200 | 0.55509300 | -1.05642000 |
| H | 3.90074300 | -1.06619900 | -0.81188800 |
| C | 1.31901900 | -0.11782000 | -0.54631500 |
| H | 0.91688300 | -0.06317500 | -1.55404800 |

trans-R12-P

| | | | |
|---|---|---|---|
| C | 3.16864600 | 0.91992900 | 0.92949700 |
| C | 2.60370200 | 0.27289800 | -0.30383700 |
| C | 3.83703900 | -0.29446700 | 0.33904000 |
| H | 3.66794000 | 1.87457000 | 0.82944100 |
| H | 2.61484800 | 0.78986800 | 1.85130600 |
| H | 2.75048900 | 0.80744700 | -1.23767600 |
| H | 3.72963000 | -1.23351200 | 0.86764300 |
| H | 4.78777100 | -0.15967100 | -0.15948300 |

| | | | |
|---|---|---|---|
| C | 1.31411300 | -0.47498900 | -0.21259500 |
| C | 0.07194800 | 0.11360800 | -0.81132600 |
| C | 0.70596100 | -1.09419600 | -1.44685800 |
| H | 1.17020500 | -1.03527600 | 0.70637300 |
| H | 0.19849900 | 1.05881300 | -1.33212500 |
| H | 0.20191700 | -2.04445600 | -1.33223900 |
| H | 1.22688100 | -0.95210800 | -2.38590100 |
| C | -1.25736400 | 0.03850000 | -0.10288000 |
| C | -1.50629600 | 1.25527700 | 0.83149900 |
| C | -2.45223400 | 0.48260300 | -0.99433400 |
| C | -2.41191000 | 1.78273400 | -0.24385000 |
| H | -0.61842300 | 1.83585400 | 1.09989100 |
| H | -2.03569300 | 0.96438400 | 1.74566200 |
| H | -2.26794200 | 0.49200100 | -2.07269900 |
| H | -3.35782800 | -0.10302500 | -0.79445400 |
| H | -3.00362200 | 2.68308700 | -0.32030700 |
| O | -1.31397900 | -1.25113300 | 0.48117900 |
| O | -2.50416900 | -1.36316200 | 1.24918100 |
| H | -2.96110900 | -2.07501000 | 0.78555600 |

trans-R13-R

| | | | |
|---|---|---|---|
| C | -2.94222900 | -1.50229600 | 0.23105600 |
| C | -2.61339000 | -0.17064700 | -0.38019500 |
| C | -3.92182300 | -0.36233400 | 0.33344400 |
| H | -3.18351900 | -2.32558500 | -0.42778600 |
| H | -2.39963000 | -1.78211600 | 1.12575200 |
| H | -2.65873600 | -0.11399100 | -1.46330400 |
| H | -4.03440800 | 0.11962700 | 1.29685900 |
| H | -4.82776000 | -0.41472600 | -0.25564500 |
| C | -1.55269900 | 0.67954400 | 0.23839700 |
| C | -0.22926300 | 0.85030100 | -0.45352800 |
| C | -1.19460600 | 2.00554700 | -0.37591500 |
| H | -1.51557900 | 0.63125300 | 1.32416300 |
| H | -0.13881700 | 0.36206100 | -1.41835400 |
| H | -0.94282700 | 2.82732400 | 0.28369400 |
| H | -1.71744700 | 2.29143000 | -1.28012200 |
| C | 1.03444100 | 0.90176000 | 0.35213900 |
| C | 1.79279800 | -0.40677900 | 0.65232700 |
| C | 2.34149400 | 1.45205900 | -0.27768900 |
| H | 0.84677600 | 1.40336900 | 1.30724500 |
| C | 3.11676000 | 0.35378900 | 0.49197600 |
| H | 1.58663300 | -0.97591700 | 1.55875700 |
| H | 2.35655700 | 1.27246200 | -1.35405600 |
| H | 2.59764200 | 2.49198000 | -0.08035700 |

| | | | |
|---|---|---|---|
| H | 3.91185200 | -0.18749900 | -0.01925100 |
| H | 3.47712900 | 0.70570400 | 1.45939800 |
| O | 1.59065500 | -1.30034100 | -0.46833400 |
| O | 2.36599900 | -2.33853900 | -0.39780000 |

trans-R13-TS

| | | | |
|---|---|---|---|
| C | 0.00537100 | -0.53328400 | 0.07499900 |
| C | -0.86251700 | -1.49306700 | -0.64908500 |
| H | 0.10200400 | 0.73098200 | -0.44234600 |
| H | -1.34272200 | -1.18671400 | -1.57216300 |
| H | -0.58362100 | -2.54188000 | -0.61959200 |
| C | 1.35194800 | -0.76298800 | 0.67251200 |
| C | 2.39491200 | -1.43048800 | -0.26065000 |
| C | 2.22849000 | 0.54220300 | 0.58132800 |
| H | 1.26039900 | -1.16302400 | 1.68569900 |
| C | 2.93190000 | -0.03303600 | -0.65705300 |
| H | 1.98786500 | -2.04521700 | -1.06329600 |
| H | 3.12510500 | -2.01082100 | 0.30451100 |
| H | 2.88090400 | 0.67083300 | 1.44507000 |
| H | 2.45623100 | 0.33794600 | -1.56403500 |
| H | 4.01031300 | 0.10410600 | -0.72119000 |
| O | 1.50867700 | 1.74435200 | 0.49789500 |
| O | 0.68589400 | 1.71737200 | -0.62049400 |
| C | -2.43505500 | 0.17338600 | 0.64365500 |
| C | -2.82831900 | 0.90625000 | -0.60413600 |
| C | -3.74964600 | -0.13000700 | -0.01899100 |
| H | -2.48197300 | 0.75903700 | 1.55525400 |
| H | -2.31886900 | 0.67218100 | -1.53045600 |
| H | -3.09738200 | 1.94847500 | -0.50062500 |
| H | -4.66193900 | 0.20225400 | 0.45820400 |
| H | -3.82257200 | -1.07729400 | -0.53940200 |
| C | -1.32845500 | -0.84018700 | 0.64491800 |
| H | -1.32208200 | -1.48734500 | 1.52016400 |

trans-R13-P

| | | | |
|---|---|---|---|
| C | 2.93241500 | 1.47941300 | 0.34320800 |
| C | 2.51114400 | 0.23740100 | -0.38732700 |
| C | 3.92951000 | 0.37872600 | 0.08583800 |
| H | 3.03021800 | 2.39833000 | -0.21933700 |
| H | 2.57327000 | 1.60386500 | 1.35757500 |
| H | 2.33517700 | 0.34048900 | -1.45275200 |
| H | 4.23271700 | -0.23114900 | 0.92811400 |
| H | 4.70369100 | 0.55447700 | -0.64921300 |
| C | 1.60086900 | -0.73866700 | 0.29241100 |
| C | 0.25026300 | -0.98625100 | -0.22592200 |
| C | 1.24961600 | -2.04898900 | -0.41527700 |

| | | | |
|---|---|---|---|
| H | 1.73527600 | -0.79404300 | 1.37374600 |
| H | 1.16599400 | -2.95815100 | 0.17498200 |
| H | 1.68211200 | -2.19080700 | -1.40152600 |
| C | -1.07404800 | -0.99470900 | 0.41911800 |
| C | -1.80691900 | 0.35029700 | 0.64057200 |
| C | -2.31981000 | -1.49149200 | -0.36012600 |
| H | -1.01467100 | -1.50921600 | 1.39026900 |
| C | -3.13341700 | -0.36027700 | 0.31514800 |
| H | -1.69920700 | 0.86170000 | 1.60109200 |
| H | -2.19566600 | -1.31582700 | -1.42866600 |
| H | -2.63724900 | -2.51954500 | -0.19058500 |
| H | -3.83062700 | 0.21043100 | -0.29605100 |
| H | -3.63168700 | -0.69886100 | 1.22506500 |
| O | -1.40192500 | 1.21014700 | -0.40797100 |
| O | -2.29786400 | 2.32078800 | -0.39658000 |
| H | -1.68878300 | 3.04811700 | -0.22216000 |

trans-R14-R

| | | | |
|---|---|---|---|
| C | -2.94222900 | -1.50229600 | 0.23105600 |
| C | -2.61339000 | -0.17064700 | -0.38019500 |
| C | -3.92182300 | -0.36233400 | 0.33344400 |
| H | -3.18351900 | -2.32558500 | -0.42778600 |
| H | -2.39963000 | -1.78211600 | 1.12575200 |
| H | -2.65873600 | -0.11399100 | -1.46330400 |
| H | -4.03440800 | 0.11962700 | 1.29685900 |
| H | -4.82776000 | -0.41472600 | -0.25564500 |
| C | -1.55269900 | 0.67954400 | 0.23839700 |
| C | -0.22926300 | 0.85030100 | -0.45352800 |
| C | -1.19460600 | 2.00554700 | -0.37591500 |
| H | -1.51557900 | 0.63125300 | 1.32416300 |
| H | -0.13881700 | 0.36206100 | -1.41835400 |
| H | -0.94282700 | 2.82732400 | 0.28369400 |
| H | -1.71744700 | 2.29143000 | -1.28012200 |
| C | 1.03444100 | 0.90176000 | 0.35213900 |
| C | 1.79279800 | -0.40677900 | 0.65232700 |
| C | 2.34149400 | 1.45205900 | -0.27768900 |
| H | 0.84677600 | 1.40336900 | 1.30724500 |
| C | 3.11676000 | 0.35378900 | 0.49197600 |
| H | 1.58663300 | -0.97591700 | 1.55875700 |
| H | 2.35655700 | 1.27246200 | -1.35405600 |
| H | 2.59764200 | 2.49198000 | -0.08035700 |
| H | 3.91185200 | -0.18749900 | -0.01925100 |
| H | 3.47712900 | 0.70570400 | 1.45939800 |
| O | 1.59065500 | -1.30034100 | -0.46833400 |

| | | | |
|---|---|---|---|
| O | 2.36599900 | -2.33853900 | -0.39780000 |

trans-R14-TS

| | | | |
|---|---|---|---|
| C | 1.98623300 | 0.21499600 | 0.98508000 |
| C | 3.21163200 | -0.53727500 | 0.42544200 |
| C | 2.27141400 | -0.94394600 | -0.70975200 |
| C | 1.08332900 | -0.80678300 | 0.23151700 |
| H | 1.85626500 | 0.39037300 | 2.05120900 |
| H | 3.48145100 | -1.38248500 | 1.06412400 |
| H | 4.09248000 | 0.05731100 | 0.18650300 |
| H | 2.42705100 | -1.69239700 | -1.48382900 |
| H | 2.20607900 | 0.25654700 | -1.24707500 |
| H | 1.00131500 | -1.70837000 | 0.85419300 |
| O | 1.82763100 | 1.49523200 | 0.37829600 |
| O | 2.24269000 | 1.45665900 | -0.96278300 |
| C | -0.25927800 | -0.38214100 | -0.27727100 |
| C | -1.25465700 | -1.42781500 | -0.70831000 |
| H | -0.25631800 | 0.54232300 | -0.84621300 |
| H | -0.97420100 | -2.46736000 | -0.58838600 |
| H | -1.85818800 | -1.22605200 | -1.58390900 |
| C | -2.57533700 | 0.40485000 | 0.62574100 |
| C | -3.21125600 | 0.97930000 | -0.60669600 |
| C | -3.99523000 | 0.05031700 | 0.28048200 |
| H | -2.44754400 | 1.10592600 | 1.44349700 |
| H | -2.89776600 | 0.61151600 | -1.57537300 |
| H | -3.46347400 | 2.03099500 | -0.59156600 |
| H | -4.79630500 | 0.46429800 | 0.87826200 |
| H | -4.16719100 | -0.95394000 | -0.08711800 |
| C | -1.49507600 | -0.62927100 | 0.54273000 |
| H | -1.33687500 | -1.17720400 | 1.46780400 |

trans-R14-P

| | | | |
|---|---|---|---|
| C | -2.80386100 | 1.54137700 | 0.58782100 |
| C | -2.68713800 | 0.05281800 | 0.41723400 |
| C | -3.80021200 | 0.79751600 | -0.26326000 |
| H | -3.13775600 | 1.92797900 | 1.54159500 |
| H | -2.06796600 | 2.15283900 | 0.07968400 |
| H | -2.96640700 | -0.55392200 | 1.27318300 |
| H | -3.73041200 | 0.91561500 | -1.33751600 |
| H | -4.80666600 | 0.68130000 | 0.11629100 |
| C | -1.56529700 | -0.50248900 | -0.39744800 |
| C | -0.41275000 | -1.19019000 | 0.27893200 |
| C | -1.39487300 | -1.99080400 | -0.53653100 |
| H | -1.30463600 | 0.09317400 | -1.26886300 |
| H | -0.48757100 | -1.29345800 | 1.35805800 |
| H | -1.05429200 | -2.38266100 | -1.48727500 |

| | | | |
|---|---|---|---|
| H | -2.09759300 | -2.62310300 | -0.00805400 |
| C | 0.98247500 | -0.96550200 | -0.23662300 |
| C | 1.76254200 | 0.26401800 | 0.28943000 |
| C | 2.12596600 | -1.79396900 | 0.28492700 |
| H | 0.96795800 | -0.93255400 | -1.33739900 |
| C | 3.02666000 | -0.60750800 | 0.47628000 |
| H | 1.35928800 | 0.65060000 | 1.23107700 |
| H | 2.29045600 | -2.86183700 | 0.31833900 |
| H | 3.56483900 | -0.50904500 | 1.42148200 |
| H | 3.73273700 | -0.44615600 | -0.34817200 |
| O | 1.81735600 | 1.28428000 | -0.67849200 |
| O | 2.71050800 | 2.28083100 | -0.18385200 |
| H | 2.11403500 | 3.02874300 | -0.05826100 |

trans-R15-R

| | | | |
|---|---|---|---|
| C | -4.30713700 | -0.29375200 | -0.57124500 |
| C | -3.10522900 | 0.00777700 | 0.27787500 |
| C | -3.58050800 | -1.41034800 | 0.13223700 |
| H | -5.28396800 | -0.01042000 | -0.20251600 |
| H | -4.18603700 | -0.21302700 | -1.64442400 |
| H | -3.29943200 | 0.48491900 | 1.23362700 |
| H | -2.97454400 | -2.07443500 | -0.47201300 |
| H | -4.06644800 | -1.88139400 | 0.97634100 |
| C | -1.82734400 | 0.42592100 | -0.37308400 |
| C | -1.32557000 | 1.83465500 | -0.20701200 |
| C | -0.60691700 | 0.69155400 | 0.46323200 |
| H | -1.64239800 | -0.03659100 | -1.33951800 |
| H | -1.91387500 | 2.50075000 | 0.41192800 |
| H | -0.83415300 | 2.30845200 | -1.04811700 |
| H | -0.74903700 | 0.60250900 | 1.53809000 |
| C | 0.74559900 | 0.27154000 | -0.02591600 |
| C | 2.00403600 | 0.87231300 | 0.65745900 |
| C | 1.26862500 | -1.13525100 | 0.37168700 |
| H | 0.80928100 | 0.40130700 | -1.11007600 |
| C | 2.65279900 | -0.51626800 | 0.58790400 |
| H | 1.79608200 | 1.14699300 | 1.69393100 |
| H | 2.54442000 | 1.68034800 | 0.16509600 |
| H | 0.83911300 | -1.46526200 | 1.31973100 |
| H | 1.20821000 | -1.94123500 | -0.35989100 |
| H | 3.30191500 | -0.85796100 | 1.39322200 |
| O | 3.38908600 | -0.67283200 | -0.65067000 |
| O | 4.47850700 | 0.03110800 | -0.65360900 |

trans-R15-TS

| | | | |
|---|---|---|---|
| C | -0.96999000 | 0.41868800 | 0.05996300 |

| | | | |
|---|---|---|---|
| C | -2.11017900 | 1.07570700 | 0.83112500 |
| C | -1.77320800 | 0.60134300 | -1.23606500 |
| H | -1.33951900 | -0.82017900 | 0.24264000 |
| C | -3.01002900 | 0.55642100 | -0.31458100 |
| H | -2.03367400 | 2.16754100 | 0.79064300 |
| H | -2.33309100 | 0.74468800 | 1.84615200 |
| H | -1.63425200 | 1.60130500 | -1.65763000 |
| H | -1.69488700 | -0.15797600 | -2.01361700 |
| H | -3.93275400 | 1.06948900 | -0.57566700 |
| O | -3.40309700 | -0.78416900 | -0.01688900 |
| O | -2.27795200 | -1.62080200 | 0.03475600 |
| C | 0.48738000 | 0.59588200 | 0.25783000 |
| C | 1.19150700 | -0.13040700 | 1.37401200 |
| H | 0.85753900 | 1.59678700 | 0.03813300 |
| H | 0.59766400 | -0.80937400 | 1.97349900 |
| H | 1.96073100 | 0.40006600 | 1.92038300 |
| C | 1.45019200 | -0.51909200 | -0.05521400 |
| H | 0.98626100 | -1.45181000 | -0.36302600 |
| C | 2.75833800 | -0.23421800 | -0.72644600 |
| C | 3.72192600 | 0.78007300 | -0.17881900 |
| C | 4.04665200 | -0.68705400 | -0.09890300 |
| H | 2.71595900 | -0.28958300 | -1.80898000 |
| H | 3.47351700 | 1.29376100 | 0.74129900 |
| H | 4.26931600 | 1.38295200 | -0.89093500 |
| H | 4.82862200 | -1.07551800 | -0.73745600 |
| H | 3.97141900 | -1.15644900 | 0.87421500 |

trans-R15-P

| | | | |
|---|---|---|---|
| C | 3.97445100 | -0.98268200 | -0.65640300 |
| C | 3.27804000 | -1.56249500 | 0.54634500 |
| C | 3.06693900 | -0.12131400 | 0.17791500 |
| H | 5.04168700 | -0.81539800 | -0.59602900 |
| H | 3.61487500 | -1.27512000 | -1.63515000 |
| H | 3.87448800 | -1.78896500 | 1.42007700 |
| H | 2.45291100 | -2.24174400 | 0.37046700 |
| H | 3.55239000 | 0.61473800 | 0.81150200 |
| C | 1.75104800 | 0.31170900 | -0.37609200 |
| C | 0.80518000 | 1.13765900 | 0.49731800 |
| C | 1.50221500 | 1.75070400 | -0.69690000 |
| H | 1.26508800 | -0.41572700 | -1.02042300 |
| H | 1.20898900 | 1.39152400 | 1.47283300 |
| H | 0.88392600 | 1.98442700 | -1.55512200 |
| H | 2.30316700 | 2.45370900 | -0.50351700 |
| C | -0.62884600 | 0.89176800 | 0.45142800 |
| C | -1.54921000 | 0.23147900 | 1.44596000 |

| | | | |
|---|---|---|---|
| C | -1.54225400 | 0.60412100 | -0.71232800 |
| C | -2.62089400 | 0.24623300 | 0.33762000 |
| H | -1.78671100 | 0.75805000 | 2.37331000 |
| H | -1.25660800 | -0.79938300 | 1.68865000 |
| H | -1.77445000 | 1.41686600 | -1.40517800 |
| H | -1.26448700 | -0.28685600 | -1.29301900 |
| H | -3.38162100 | 1.02178900 | 0.45991600 |
| O | -3.22744000 | -1.01837700 | 0.21062500 |
| O | -4.02802400 | -0.97427500 | -0.96946800 |
| H | -4.90994200 | -1.11978000 | -0.60675300 |

trans-R16-R

| | | | |
|---|---|---|---|
| C | -0.61003300 | -1.50908900 | 0.65564600 |
| H | -0.82137700 | -2.34107700 | -0.00427000 |
| H | -0.24170300 | -1.78774200 | 1.63558600 |
| C | 0.95701000 | 0.55885800 | 0.79607100 |
| C | 2.43122600 | 0.21780700 | 0.46475100 |
| C | 1.23782900 | 1.94751600 | 0.15804400 |
| H | 0.71784100 | 0.55117300 | 1.86211200 |
| C | 2.45374800 | 1.33152100 | -0.58290400 |
| H | 3.09332400 | 0.40807500 | 1.30958000 |
| H | 0.43711700 | 2.35612500 | -0.45807400 |
| H | 1.54120900 | 2.68771300 | 0.89912700 |
| H | 2.16602100 | 0.93791900 | -1.55702300 |
| H | 3.36667200 | 1.91822100 | -0.67130600 |
| O | 2.75917500 | -1.13174700 | 0.08678800 |
| O | 2.32581100 | -1.46205300 | -1.09173500 |
| C | -1.48161600 | -0.28717200 | 0.54030400 |
| H | -1.67581700 | 0.25354900 | 1.46345300 |
| C | -2.61215500 | -0.26512800 | -0.43576900 |
| C | -4.02727400 | -0.25203400 | 0.06753100 |
| C | -3.44353000 | 0.97868900 | -0.57662900 |
| H | -2.43032200 | -0.82854500 | -1.34570800 |
| H | -4.16951300 | -0.23774200 | 1.14110800 |
| H | -4.77558000 | -0.80773300 | -0.48167500 |
| H | -3.79774200 | 1.25470000 | -1.56096100 |
| H | -3.19649900 | 1.81345300 | 0.06812500 |
| C | -0.06332700 | -0.24873400 | 0.04232500 |
| H | 0.05521400 | -0.26108800 | -1.03672100 |

trans-R16-TS

| | | | |
|---|---|---|---|
| C | 0.09147900 | -0.58220100 | 0.59747800 |
| C | -0.80106500 | -1.50111800 | 1.34434700 |
| H | 0.44433000 | -0.98573500 | -0.66018800 |
| H | -1.14328200 | -2.40896300 | 0.85889400 |

| | | | |
|---|---|---|---|
| H | -0.65859900 | -1.58367100 | 2.41726300 |
| C | 1.31707600 | 0.11572600 | 1.08430600 |
| C | 2.46249100 | 0.03135600 | 0.00463000 |
| C | 1.33795600 | 1.64971100 | 0.85152000 |
| H | 1.60962900 | -0.24830000 | 2.07181000 |
| C | 2.07720800 | 1.43223800 | -0.49100000 |
| H | 3.45347200 | -0.02076000 | 0.45589600 |
| H | 0.36459400 | 2.13773400 | 0.80275000 |
| H | 1.96099900 | 2.15586700 | 1.58991800 |
| H | 1.37686800 | 1.35537600 | -1.32219000 |
| H | 2.88961700 | 2.10949100 | -0.75010400 |
| O | 2.40279900 | -1.07624900 | -0.85386200 |
| O | 1.19621100 | -1.06740000 | -1.54107200 |
| C | -1.32808700 | -0.19274300 | 0.76644900 |
| H | -1.51724200 | 0.61189000 | 1.47646100 |
| C | -2.27810600 | -0.22833400 | -0.38832000 |
| C | -3.70977900 | 0.17462400 | -0.18382300 |
| C | -2.77773600 | 1.06778700 | -0.96067700 |
| H | -2.09177700 | -1.02937600 | -1.09671700 |
| H | -3.99763900 | 0.49258800 | 0.81069200 |
| H | -4.47657700 | -0.35871500 | -0.72947700 |
| H | -2.91048500 | 1.13801500 | -2.03189400 |
| H | -2.44461900 | 1.98147000 | -0.48309900 |

trans-R16-P

| | | | |
|---|---|---|---|
| C | 3.44259300 | 0.93004700 | 0.42706200 |
| C | 2.47589800 | 0.07314600 | -0.34307000 |
| C | 3.95497200 | -0.11703500 | -0.52639800 |
| H | 3.54787600 | 1.96992100 | 0.14675800 |
| H | 3.53884100 | 0.73224600 | 1.48769500 |
| H | 1.94985000 | 0.55820500 | -1.15854100 |
| H | 4.39369000 | -1.01155700 | -0.10168600 |
| H | 4.40529700 | 0.21400400 | -1.45270900 |
| C | 1.68024500 | -0.96873800 | 0.38218300 |
| C | 0.26195600 | -0.74124500 | 0.69428800 |
| C | 0.56907800 | -1.73584400 | -0.33871200 |
| H | 2.26040700 | -1.55469800 | 1.09431900 |
| H | 0.45691500 | -2.78979900 | -0.10586900 |
| H | 0.36379000 | -1.48315200 | -1.37669200 |
| C | -0.55475900 | 0.48570900 | 0.68943600 |
| C | -0.65520700 | 1.37929400 | -0.59337000 |
| C | -2.09822400 | 0.38188700 | 0.65471200 |
| H | -0.28131900 | 1.12413000 | 1.53463000 |
| C | -2.13364300 | 1.64249400 | -0.22644600 |
| H | -0.57341300 | 0.77465300 | -1.49724200 |

| | | | |
|---|---|---|---|
| H | 0.02334200 | 2.22961200 | -0.65206600 |
| H | -2.64514300 | 0.37688600 | 1.60143300 |
| H | -2.87347900 | 1.63641800 | -1.02517200 |
| H | -2.24954700 | 2.54229700 | 0.37962000 |
| O | -2.41818300 | -0.77777800 | -0.09067700 |
| O | -3.81017300 | -0.70083500 | -0.39376600 |
| H | -4.14771100 | -1.46854900 | 0.08295700 |

trans-R17-R

| | | | |
|---|---|---|---|
| C | -0.61003300 | -1.50908900 | 0.65564600 |
| H | -0.82137700 | -2.34107700 | -0.00427000 |
| H | -0.24170300 | -1.78774200 | 1.63558600 |
| C | 0.95701000 | 0.55885800 | 0.79607100 |
| C | 2.43122600 | 0.21780700 | 0.46475100 |
| C | 1.23782900 | 1.94751600 | 0.15804400 |
| H | 0.71784100 | 0.55117300 | 1.86211200 |
| C | 2.45374800 | 1.33152100 | -0.58290400 |
| H | 3.09332400 | 0.40807500 | 1.30958000 |
| H | 0.43711700 | 2.35612500 | -0.45807400 |
| H | 1.54120900 | 2.68771300 | 0.89912700 |
| H | 2.16602100 | 0.93791900 | -1.55702300 |
| H | 3.36667200 | 1.91822100 | -0.67130600 |
| O | 2.75917500 | -1.13174700 | 0.08678800 |
| O | 2.32581100 | -1.46205300 | -1.09173500 |
| C | -1.48161600 | -0.28717200 | 0.54030400 |
| H | -1.67581700 | 0.25354900 | 1.46345300 |
| C | -2.61215500 | -0.26512800 | -0.43576900 |
| C | -4.02727400 | -0.25203400 | 0.06753100 |
| C | -3.44353000 | 0.97868900 | -0.57662900 |
| H | -2.43032200 | -0.82854500 | -1.34570800 |
| H | -4.16951300 | -0.23774200 | 1.14110800 |
| H | -4.77558000 | -0.80773300 | -0.48167500 |
| H | -3.79774200 | 1.25470000 | -1.56096100 |
| H | -3.19649900 | 1.81345300 | 0.06812500 |
| C | -0.06332700 | -0.24873400 | 0.04232500 |
| H | 0.05521400 | -0.26108800 | -1.03672100 |

trans-R17-TS

| | | | |
|---|---|---|---|
| C | -1.54122200 | -1.10012900 | 0.85269100 |
| C | -2.93446800 | -0.51012500 | 1.07383500 |
| C | -0.97531100 | 0.30463600 | 0.70401900 |
| H | -1.71951700 | -1.35242400 | -0.42770200 |
| C | -2.41400900 | 0.67589900 | 0.23512300 |
| H | -3.10080800 | -0.21561200 | 2.11338800 |
| H | -3.79760400 | -1.05061500 | 0.68733800 |

| | | | |
|---|---|---|---|
| H | -0.82007900 | 0.74680000 | 1.69791300 |
| H | -2.79804500 | 1.68563300 | 0.36566700 |
| O | -2.51478500 | 0.42024300 | -1.16388700 |
| O | -2.31354900 | -0.94411300 | -1.42870600 |
| H | -1.08165200 | -1.95687700 | 1.34109600 |
| C | 0.19441900 | 0.57326800 | -0.19127200 |
| C | 0.98743000 | 1.84247500 | -0.01659600 |
| H | 0.06918900 | 0.21762500 | -1.20912600 |
| H | 0.68731200 | 2.51888100 | 0.77485900 |
| H | 1.36881200 | 2.32480200 | -0.90777100 |
| C | 1.58995700 | 0.51893500 | 0.36673500 |
| H | 1.66675500 | 0.32051100 | 1.43321200 |
| C | 2.69707700 | -0.06297400 | -0.44973300 |
| C | 4.08735500 | -0.11721300 | 0.11700700 |
| C | 3.29183800 | -1.38501800 | -0.05623000 |
| H | 2.61777700 | 0.13095700 | -1.51481000 |
| H | 4.22512900 | 0.25594700 | 1.12440100 |
| H | 4.92287500 | 0.05460700 | -0.54842300 |
| H | 3.58843900 | -2.07033000 | -0.83897800 |
| H | 2.89969100 | -1.85746600 | 0.83621700 |

trans-R17-P

| | | | |
|---|---|---|---|
| C | 3.86379800 | 0.99216100 | -0.00288800 |
| C | 3.02750600 | -0.18789000 | -0.40937500 |
| C | 4.30989800 | -0.40474600 | 0.34272200 |
| H | 4.40981700 | 1.52630000 | -0.76907100 |
| H | 3.48505600 | 1.61181900 | 0.80069900 |
| H | 3.03942000 | -0.44580600 | -1.46392400 |
| H | 4.22865700 | -0.71693900 | 1.37658300 |
| H | 5.15768700 | -0.81487500 | -0.18976700 |
| C | 1.72192500 | -0.43548600 | 0.27273100 |
| C | 0.43188300 | -0.20383700 | -0.46185000 |
| C | 0.87298300 | -1.60838000 | -0.13666800 |
| H | 1.71254800 | -0.18428000 | 1.33046900 |
| H | 0.52888900 | 0.10857500 | -1.49804100 |
| H | 0.31834400 | -2.13741500 | 0.62886500 |
| H | 1.24041600 | -2.22684800 | -0.94621400 |
| C | -0.75321200 | 0.37510000 | 0.26191300 |
| C | -1.07656700 | 1.83832900 | 0.12201000 |
| C | -2.16688400 | 0.10828800 | -0.31113100 |
| H | -0.73305000 | 0.05575700 | 1.31577500 |
| C | -2.54084700 | 1.59530300 | -0.10494200 |
| H | -0.49089300 | 2.72973800 | 0.29795600 |
| H | -2.15002200 | -0.19299300 | -1.36339600 |
| H | -3.02976100 | 2.10580600 | -0.93752500 |

| | | | |
|---|---|---|---|
| H | -3.15891500 | 1.71798500 | 0.79344600 |
| O | -2.85671600 | -0.84277300 | 0.46316400 |
| O | -4.19904800 | -0.87865400 | -0.01918700 |
| H | -4.25497600 | -1.77846000 | -0.36280800 |
